# Searching for visual features that explain response variance of face neurons in inferior temporal cortex

**DOI:** 10.1371/journal.pone.0201192

**Published:** 2018-09-20

**Authors:** Takashi Owaki, Michel Vidal-Naquet, Yunjun Nam, Go Uchida, Takayuki Sato, Hideyuki Câteau, Shimon Ullman, Manabu Tanifuji

**Affiliations:** 1 Toyota Central R&D Labs., Inc., Nagakute, Aichi, Japan; 2 Laboratory for Integrative Neural Systems, Center for Brain Science, Wako-shi, Saitama, Japan; 3 BTCC, RIKEN Brain Science Institute, Wako-shi, Saitama, Japan; 4 University of Tsukuba, Ten-nodai, Tsukuba, Ibaraki, Japan; 5 Department of Computer Science and Applied Mathematics, The Weizmann Institute of Science, Rehovot, Israel; 6 Department of Complexity Science and Engineering, Graduate School of Frontier Sciences, The University of Tokyo, Kashiwa, Chiba, Japan; 7 Department of Life Science and Medical Bio-Science, Faculty of Science and Engineering, Waseda University, Shinjuku, Tokyo, Japan; University of Toyama, JAPAN

## Abstract

Despite a large body of research on response properties of neurons in the inferior temporal (IT) cortex, studies to date have not yet produced quantitative feature descriptions that can predict responses to arbitrary objects. This deficit in the research prevents a thorough understanding of object representation in the IT cortex. Here we propose a fragment-based approach for finding quantitative feature descriptions of face neurons in the IT cortex. The development of the proposed method was driven by the assumption that it is possible to recover features from a set of natural image fragments if the set is sufficiently large. To find the feature from the set, we compared object responses predicted from each fragment and responses of neurons to these objects, and search for the fragment that revealed the highest correlation with neural object responses. Prediction of object responses of each fragment was made by normalizing Euclidian distance between the fragment and each object to 0 to 1 such that the smaller distance gives the higher value. The distance was calculated at the space where images were transformed to a local orientation space by a Gabor filter and a local max operation. The method allowed us to find features with a correlation coefficient between predicted and neural responses of 0.68 on average (number of object stimuli, 104) from among 560,000 feature candidates, reliably explaining differential responses among faces as well as a general preference for faces over to non-face objects. Furthermore, predicted responses of the resulting features to novel object images were significantly correlated with neural responses to these images. Identification of features comprising specific, moderately complex combinations of local orientations and colors enabled us to predict responses to upright and inverted faces, which provided a possible mechanism of face inversion effects. (292/300).

## Introduction

We recognize objects invariantly across large variations of view, position, photometric conditions, and a shape deformation. This behavioral property is not trivial, because there is an enormous number of objects belonging to the same category, and typically larger differences in retinal images arise from variations of an object than from different objects in the same category.

There is an accumulated body of evidence that objects are represented in the inferior temporal (IT) cortex as combinations of neurons, each encoding a visual feature less complex than the complete object [1–4]. One hypothetical framework for invariant object recognition is that various appearances of an object are represented separately from other objects in the visual feature space in the IT cortex [5]. In support of this thesis, Hung and colleagues recorded neuronal responses to objects of various sizes and positions in monkey IT cortex, and found that these objects were well-separated in the neural response space [6]. Other studies suggest that view-invariant representation of faces is achieved by the population responses of neurons in the most anterior part of the IT cortex [7]. However, we still do not understand the general property of visual features that is critical to making object representation invariant. Although an attempt has been made to address this question in terms of position invariance, our understanding is still very limited [8].

Quantitative description of the features of IT neurons is essential for developing a fundamental understanding of invariant object representation. A previous approach involved simplification of the best object stimuli to determine the simplest and most effective visual feature that can activate individual neurons [1,2]. More recently, the recorded responses of IT neurons to a parameterized artificial stimulus set were analyzed to explore the critical parameters explaining responses to the stimuli [4]. Although these studies provided insight into the visual features encoded by IT neurons, they did not sufficiently address object representation in the visual feature space. Description of visual features was qualitative in the former approach, and it is difficult to place real world objects in their parameterized space in the latter approach.

In this paper, we proposed a fragment-based approach to identify quantitatively described features of IT neurons. In this approach, we searched for the features in the subset of regions in natural images, which we considered to be a reasonable strategy for the following reasons. First, because the IT cortex is essentially dedicated to object vision in natural scenes, it makes sense to search for features in natural images, even though such images comprise only a small subset of possible images. Second, these features typically consisting of intermediate complexity can be generalized across object categories if the subset includes a sufficiently large number of elements [2,3]. Finally, this hypothesis is consistent with previous qualitative studies showing that IT neurons encode moderately complex visual features of object images [1–3,9]. Based on these considerations, we prepared a natural image fragment set including a huge number of image fragments where we search for the features of neurons. With this approach, we found features that explained up to 67% of the variance (45% on average; number of object stimuli, 104) in the object responses of neurons within a face selective region in the anterior IT cortex. Furthermore, predicted responses of the resulting features to novel object images were significantly correlated with neural responses to these images: the correlation coefficient was 0.59 ± 0.06 on average (number of object stimuli, 500) when we searched for the best feature, and was 0.53 ± 0.07 when we calculated for novel 500 objects with the best feature. To the best of our knowledge, no previous studies have involved quantitative searching for visual features that explain object responses for IT neurons. The performance of our method is remarkably high in comparison to the reasonable approaches used in a study in earlier visual area [10].

## Materials and methods

### The physiological dataset

We developed the fragment-based approach using three physiological datasets. Two of them were obtained from two hemispheres of adult male macaque monkeys (*Macaca mulatta*) in the previous study (H1, and H2) [11]. One dataset (H3) was a new dataset obtained from a hemisphere of another adult male macaque monkey (*Macaca mulatta*) in a similar way. The datasets comprised columnar responses to object images from 39 (H1), 36 (H2) and 119 (H3) sites. The columnar response of a site was calculated by averaging fifteen (H1 and H2) or eight (H3) multi-unit (MU) activities recorded along the axis perpendicular to the cortical surface. In the recordings from H1 and H2, we used electrode bundles made of three tungsten microelectrodes (shaft diameter, 150 μm; impedance, 1 MΩ; #UEWLEJTMNN1E, FHC) [12]. The shafts of the electrodes were pasted together with glue to set the tip-to-tip distance of electrodes to 150 μm. The electrode bundle was penetrated perpendicularly to the cortex surface, and three MU activities were recorded simultaneously at the tips. We recorded from 5 depths (every 300 μm step from depth 0 to 1200 μm; here, the depth 0 is defined as the depth where we encountered the first MU activities when penetrating the electrode bundle), so that altogether we recorded 15 MU activities for each penetration. In H3, we used a commercially available electrode array (A8×8-5mm200-200-413, Neuronexus Technologies, Inc.). The electrode array consists of 8 shanks and 8 electrical contacts each. The spacing between shanks and between electrode contacts was 0.2 mm. We used MU activities recorded from 8 contacts of each shank to calculate a columnar response. We took average of MUs based on our study showing that average of local MU activities captures the response property common across neurons in a functional column [12] (see also [13]). During recordings, stimulus images were presented to the animals and differences between mean firing rates before and during stimulus presentation were calculated to obtain responses to the stimulus images [11]. The stimulus images in H1 and H2 comprised 104 object images in six object categories: front view faces (eight humans and eight monkeys); scrambled faces (four humans and four monkeys); monkey hands (*n* = 16); animal bodies (16 monkeys and 16 other species); foods and vegetables (*n* = 16); and artificial objects (*n* = 16). The stimulus images in H3 comprised 1,000 object images in seven categories: view-controlled faces (231 humans and 70 monkeys); random-view faces (one human and 158 monkeys); pictorial plane rotation faces (28 humans and 35 monkeys); size changed faces (4 humans and 5 monkeys); scrambled faces (3 humans and 5 monkeys); pixel shuffled faces (35 monkeys); and randomly chosen 425 non-face objects. The size of stimulus images for physiological recordings was 400 × 400 pixels (20 × 20 deg. in visual angle). Objects were placed at the center of the stimulus images and their size was about 10–15 deg. in visual angle. Background of objects was filled with intermediate gray (see S1 File (104 stimuli for H1 and H2) and S2 File (1,000 stimuli for H3) files for the stimulus images).

In the previous study [11], we found that these recording sites were clustered on the cortex with respect to object response selectivity. Based on the most preferred object category, we defined clusters as “face domain” and “monkey-body domain”. We also identified “anti-face domains” as the cluster with the least responses to face category. In the present study, we focused on the recording sites in face domains unless otherwise noted. The numbers of the recording sites in face domains were 16, 19, and 16 sites in hemispheres H1, H2, and H3, respectively. The face domains potentially correspond to AL face patch in a previous study according to locations relative to the sulci and in the anterior-posterior coordinate axis [14].

Both the physiological recordings in the previous study and those for the new dataset were approved by the Experimental Animal Committee of the RIKEN Institute that is authorized to evaluate the ethics on non-human primate studies, and followed the guidelines of the RIKEN Institute and the National Institutes of Health, USA. Before the physiological recordings, titanium post for the head fixation and ring-shaped recording chamber (inner diameter, 18.0 mm) were attached on the skull. The chamber was placed just above the target recording area, that is, the anterior and dorsal part of IT cortex (TEad) which is located 15.0–20.0 mm anterior to the ear bar position below the superior temporal sulcus (STS) and just above the anterior middle temporal sulcus (AMTS) [11]. The bone and dura inside the chamber was removed, and the exposed cortex was covered with a transparent artificial dura made of silicone rubber (Arieli et al., 2002). The chamber was then filled with 25 mg/ml agarose (Nacalai Tesque, Agarose-HGS) and covered with a transparent plastic coverslip. During recordings, the transparent plastic coverslip was replaced with the one with a small hole where a recording electrode was inserted. During the above surgical procedure and physiological recordings for the new dataset, we anesthetized the animals with ketamine (5mg/kg body weight, i.m.), droperidol (0.25 mg/kg body weight, i.m.), and artificially ventilated with a mixture of N_2_O, O_2_, and isoflurane (70% N_2_O, 30% O_2_, isoflurane up to 0.5%). Fentanyl citrate (0.83 g/kg/h) was infused intravenously and continuously throughout the surgery and recordings to remove pain. EEG, ECG, expired CO_2_ concentration, and rectal temperature were monitored throughout the experiments. Body temperature was maintained at 37.6°C, and expired CO_2_ concentration between 4.0 and 5.0%. In physiological recordings, we additionally gave intravenous injection of vecuronium bromide (0.067 mg/kg/h) to paralyze the monkeys. In the surgical procedure to place the chamber in the previous study [11], we anesthetized the monkeys with ketamine (5mg/kg body weight, i.m.), droperidol (0.25 mg/kg body weight, i.m.) and intraperitoneal injection of pentobarbital sodium 35 mg/kg at the beginning. We maintained deep anesthesia by supplemental intravenous injections of pentobarbital sodium 5–10 mg. The condition of anesthesia in the physiological recordings was the same as described above for the new dataset. Please also refer to the previous study for the protocol of the physiological recordings [11].

Daily care of the monkeys was handled by the Division of Research Resource Center at RIKEN Brain Science Institute. The monkeys were from different origins and thus housed in individual cages (700 mm width, 1536 mm height and 750 mm depth) with other animals visible in the room. The room light is controlled in a cycle of a 12-hr on and 12-hr off condition. The monkeys were fed with food pellets (PS-A, Oriental Yeast Co., Ltd., Tokyo, Japan) and water ad libitum, and fruits (apple, 1/4, banana, 1/2 and one piece of potato) every day. The general health condition was daily monitored, and body weight of each monkey was recorded once a month. In-house veterinary doctor monitored and kept the monkeys in a good health condition. At the end of the entire experiments, the monkeys were sacrificed with intramuscular injection of ketamine hydrochloride (10 mg/kg), and intraperitoneal administration of pentobarbitone (50–70 mg/kg).

### The size adjustment of stimulus images for feature identification

In the fragment based approach, we resized the stimulus images from 400 × 400 to 200 × 200 pixels using bicubic interpolation. The 200 × 200 pixel images were then embedded into slightly larger gray background images where the gray level was adjusted to the gray level surrounding the objects. The purpose of this is to apply Gabor filtering and local max operation to entire 200 × 200 pixels of these images. The size of background images was made large enough to make it possible.

### Natural image fragment sets (standard set)

**Fig 1:**
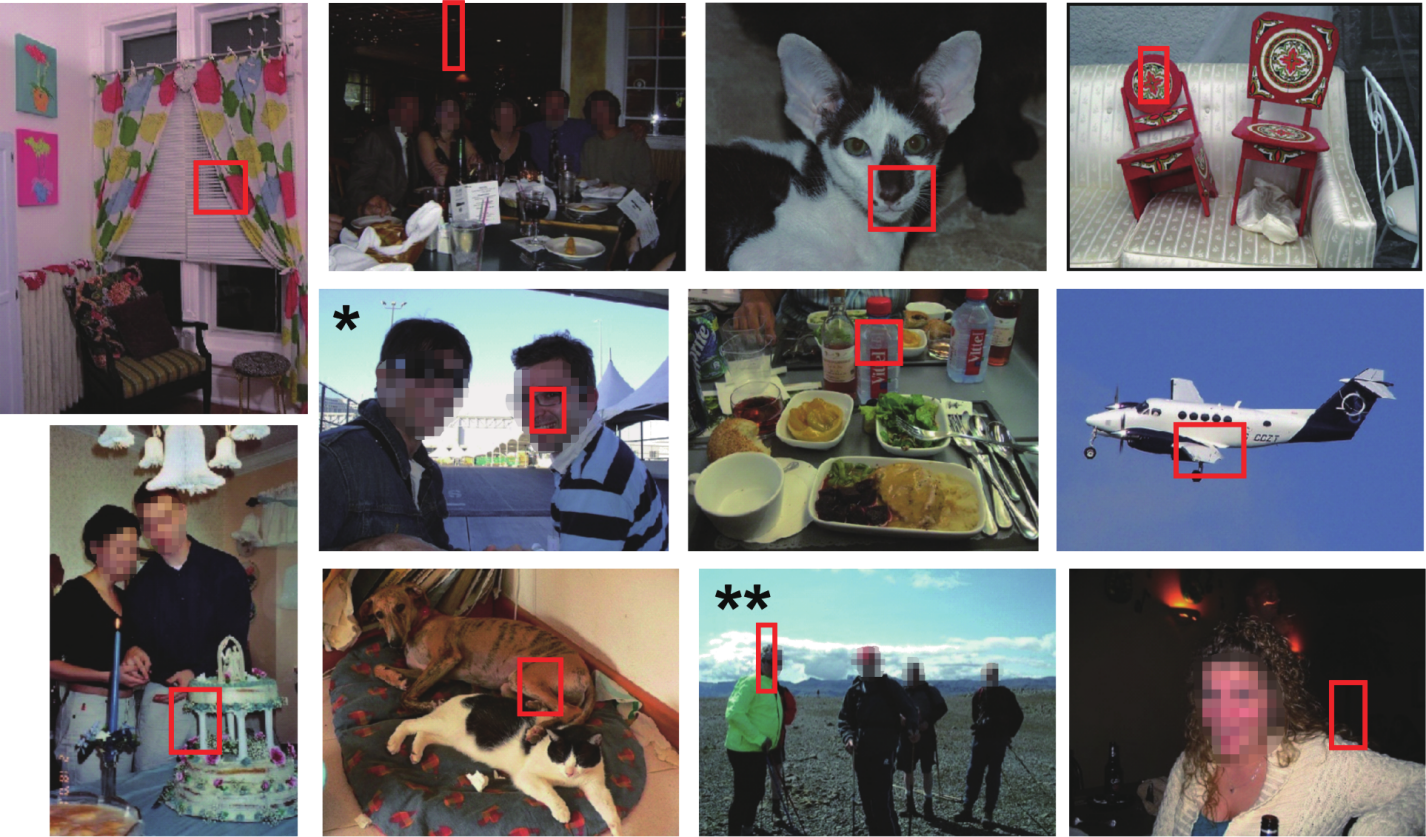
Representative natural images from which fragments were extracted. Red rectangles indicate image regions in which the best features were identified. Many other candidate fragments were cut out from each image but are not explicitly indicated (See Materials and Methods). Images with single and double asterisks include fragments, where the best features of multiple recording sites were identified. These fragments include part of faces.

We searched for features of neurons from the natural image fragment set (standard set) comprising image fragments extracted from 7,753 natural images taken from the VOC2010 dataset [15] (Fig 1). The stimulus images were not included in the standard set.

As well as the stimulus images, we applied Gabor filtering and local max operation to individual fragments on the process of the fragment-based approach. To avoid errors associated with discontinuity at the edge of each fragment, we first applied the above filtering and max operation to original 7,753 natural images (Band 1), and then cut out the fragments instead of applying these operations after cutting out pixel space fragments (see below; see also S1 Fig). To distinguish these fragments from the ones at pixel space, we use a term, ‘feature candidates’ or simply ‘features’ for the fragments cut out after Gabor filtering and local max operation. The feature candidates were in the shapes of squares (8 × 8, 12 × 12, 16 × 16, and 20 × 20 pixels for images) and rectangles with height-to-width ratios of 1:4 (2 × 8, 3 × 12, 4 × 16, 5 × 20), 1:2 (4 × 8, 6 × 12, 8 × 16, 10 × 20), 3:4 (6 × 8, 9 × 12, 12 × 16, and 15 × 20), 4:1 (8 × 2, 12 × 3, 16 × 4, and 20 × 5), 2:1 (8 × 4, 12 × 6, 16 × 8, and 20 × 10), and 4:3 (8 × 6, 12 × 9, 16 × 12, and 20 × 15), resulting in a total of 28 feature types by shape and size. The features were cut out from the natural images without gap among them to collect as many as possible features. Please also keep in mind that there is neither partial overlap among features nor inclusion of a feature in another. (see S1 Fig for representative natural images in which fragments are indicated). Altogether, there were 560,000 feature candidates resulting from 28 types and 20,000 for each.

### Outline of the fragment-based approach

**Fig 2:**
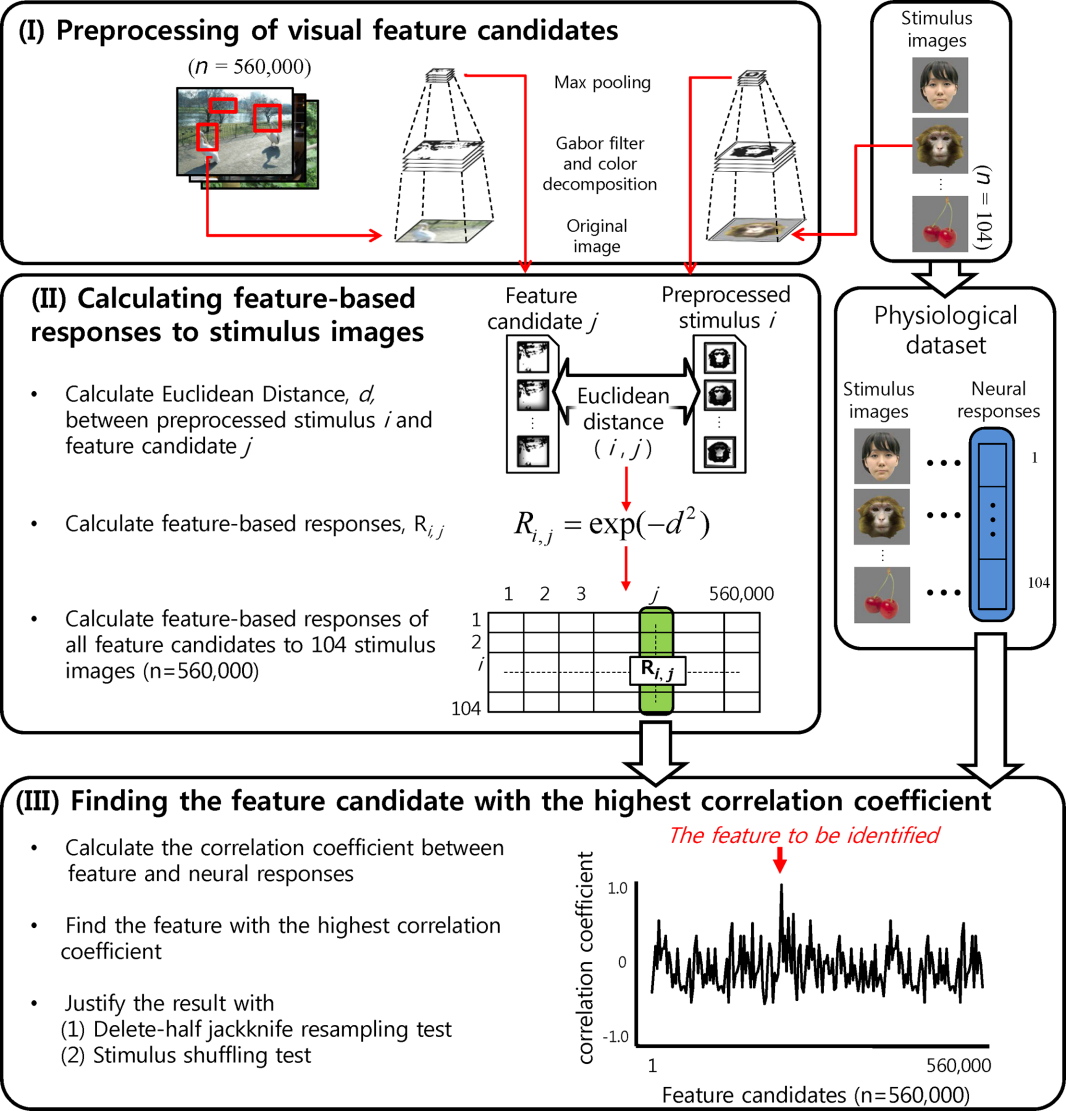
Outline of procedure for searching for visual features that explain neural responses. The physiological dataset comprises neural responses of each site to stimulus images, which are used to construct a neural response vector (right blue-shaded vertical array). Similarities between preprocessed feature candidates and stimulus images provide a table of feature-based responses in which each vertical column corresponds to a feature-based response vector (left green-shaded vertical array). We calculate the correlation coefficients between the neural response vector and all feature-based response vectors to determine the feature with the maximum correlation coefficient (lower panel). Please note that the natural images for extracting feature candidates and the stimulus images for neural recordings are completely independent sets.

Our method involves the following three steps (Fig 2). Step I is the preprocessing of natural images to prepare feature candidates and of stimulus images. The preprocessing is essentially to convert pixel-based images into representations based on local orientations and colors. In Step II, we calculated the similarity between feature candidates and the preprocessed stimulus images. We use a term, ‘feature-based response’ for the score of similarity between a feature candidate and a stimulus image, and define a feature-based response vector of each feature as the set of feature-based responses for all stimulus images (Fig 2, vertical array colored green in the left). In contrast, ‘neural response vector’ defined for each recording site indicates a vector where each element represents the neural response to a stimulus image (Fig 2, vertical array colored blue). Finally, in Step III, we selected the feature with the maximum correlation coefficient (c.c.) between the feature-based response vectors and the neural response vector [16]. We justified the result using delete-half jackknife resampling [17] and stimulus-shuffling test. Details of each step are given below.

### Preprocessing of visual feature candidates (step I)

**Fig 3:**
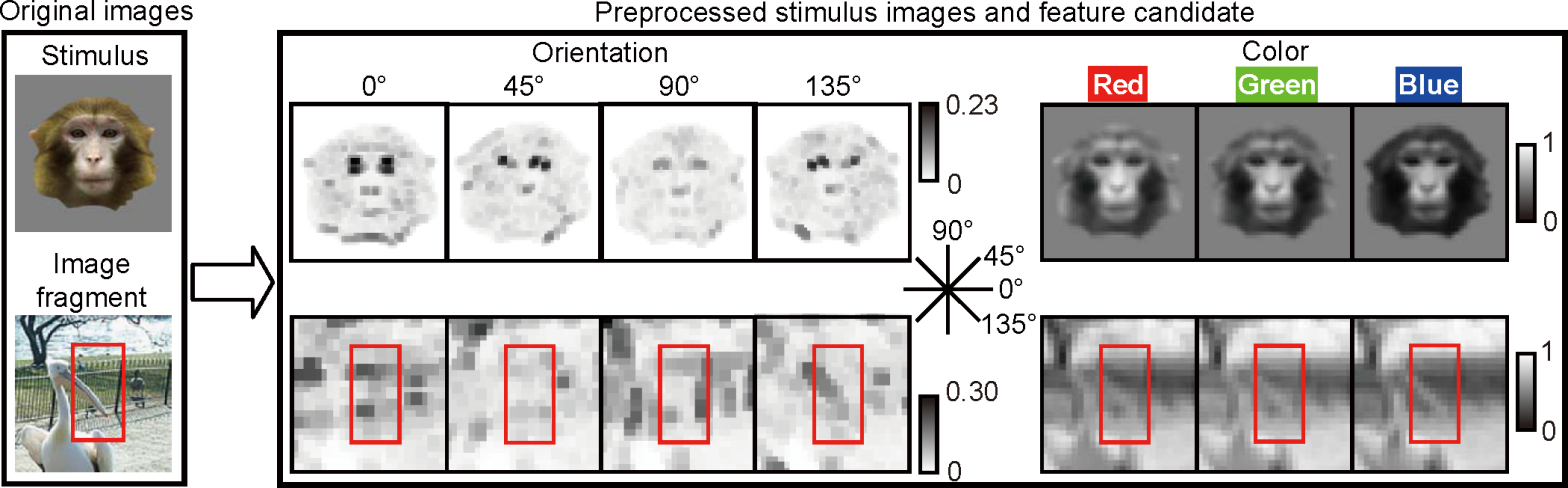
**Preprocessing of a stimulus image (top) and an image fragment (bottom).** Left panel, a representative stimulus image and a fragment. Right panel, a preprocessed image and a fragment with four orientations and three color matrices (Band 1). Magnitudes of elements in each matrix are given in a gray scale (see Materials and Methods). Please note that the stimulus images were also preprocessed with other scale bands as well.

Visual stimuli are preprocessed by neurons in the primary visual cortex which extract local edges and color with some position invariance from visual stimuli [18]. These preprocessing can be emulated by Gabor function and local MAX operation. Therefore, we preprocessed both the stimulus images and the natural images in terms of orientation and color (Fig 3). We used the Cortical Network Simulator (CNS) for these calculations [19].

To preprocess images with respect to orientation, images were converted to gray scale and then convolved using the Gabor function *F*(*x*,*y*):

$$F(x,y)=exp\;\left(-\frac{x_{0}^{2}+0.3^{2}y_{0}^{2}}{2\sigma^{2}}\right)\;\cos\;\left(\frac{2\pi }{\lambda }x_{0}\right),$$ (1)

where

$$\left(\begin{array}{c} x_{0}\\ y_{0} \end{array}\right)=\left(\begin{array}{cc} \cos\theta & \sin\theta \\ -\sin\theta & \cos\theta \end{array}\right)\;\left(\begin{array}{c} x\\ y \end{array}\right).$$

and *θ* represents the orientation channel (*θ* = 0,45,90,135 deg.). The convolved images were fed to a spatially local max operation following a previous study [20]. There were eight spatial scales coded as Band *b* (*b* = 1,2,⋯,8) for Gabor filtering and the local max operation. The Gabor function parameters (unit, pixels) to the respective Band 1–8 images were 7 × 7, 11 × 11, 15 × 15, 19 × 19, 23 × 23, 27 × 27, 31 × 31, 35 × 35 for filter size, 2.8, 4.5, 6.3, 8.2, 10.2, 12.3, 14.6, 17.0 for *σ*, and 3.5, 5.6, 7.9, 10.3, 12.7, 15.4, 18.2, 21.2 for *λ*.

These parameters were chosen to take into account the property of neurons in primary visual cortex. For example, filter size, 7 × 7, with *σ* = 2.8 and *λ* = 3.5 corresponds to filter size, 0.7 × 0.7 deg., with *σ* = 0.28 deg. and *λ* = 0.35 deg. in visual angle. These parameters in visual angle are within an observed range of neurons in monkey visual cortex. After the Gabor function to each pixel, we applied the local max operation. The window sizes of local max operation over position for Bands 1–8 were 8 × 8, 10 × 10, 12 × 12, 14 × 14, 16 × 16, 18 × 18, 20 × 20, and 22 × 22, in pixels, respectively. The strides for the max operation were 4, 5, 6, 7, 8, 9, 10, and 11 pixels for Bands 1–8, respectively.

To preprocess images with respect to color, we applied local spatial averaging to each color component (R, G, or B with pixel values ranging from 0 to 255) of the images to be preprocessed. The local averaging operation was conducted independently at the eight spatial scales (Bands 1–8). We set the window size and overlap between windows of the local averaging operation to be the same as the corresponding values used in the local max operation in the orientation channels.

The natural images were preprocessed only in the Band 1 spatial scale and then natural image fragments were cut out as described above. On the other hand, the stimulus images were preprocessed independently at eight spatial scales coded as Band *b* (*b* = 1,2,⋯,8), resulting in eight preprocessed images, which we refer to as the Band *b* images. We used these band images to take into account scale invariance of IT neurons as described in the following section (see Eq (2)). We defined α as a parameter indicating the relative weight between orientation and color channels and the scale-range, *S*_*k*_, as the set of consecutive scale bands (Bands 1 to 8), where *k* specifies a combination. The scale-ranges included combinations of one to eight band(s). There are 8 scale-ranges with one band: Band 1, Band 2,…, and Band 8). The number of scale-ranges with two consecutive scale bands is 7: Bands 1 and 2, Bands 2 and 3,…, and Bands 7 and 8. Similarly, the numbers of scale-ranges with three, four, five, six, seven, and eight are 6, 5, 4, 3, 2, and 1, respectively. The total number of possible consecutive combinations is 36 (8+7+6+5+4+3+2+1). The number of elements (bands) in each set ranged from one to eight. We assigned a range *S*_*k*_ to each preprocessed fragment and calculate the responses of a preprocessed fragment to stimulus images using the scale bands specified by *S*_*k*_.

Altogether, each preprocessed fragment was characterized by a region in a natural image, a relative weight between orientation and color channels (*α*), and a scale-range (*S*_*k*_) assigned to the fragment.

### Calculating responses of features to stimulus images (step II)

We calculated an estimated response to the *j*-th preprocessed stimulus image based on the *i*-th feature candidate (feature-based response, $R_{i,j}^{\alpha ,S_{k}}$) using a radial basis function as follows:

$$R_{i,j}^{\alpha ,S_{k}}=\underset{b\in S_{k}}{\max}\underset{x_{b},y_{b}}{\max}\exp\;\left[-\{\alpha {\left|d_{i,j,b}^{Ori.}(x_{b},y_{b})\right|}^{2}+(1-\alpha ){\left|d_{i,j,b}^{Col.}(x_{b},y_{b})\right|}^{2}\}\right]$$ (2)

where $d_{i,j,b}^{Ori.}$ and $d_{i,j,b}^{Col.}$ represent vectors whose elements are the Euclidean distances between the feature candidate and the preprocessed stimulus image in terms of the four orientations and three color matrices, respectively. The relative weight between the orientation and color channels, *α*, takes eleven possible values (*α* = 0,0.1,0.2,0.3,0.4,0.5,0.6,0.7,0.8,0.9,1.0). We define *b* as an element in the set *S*_*k*_ that specifies one of the eight scale bands (*b* = 1,⋯,8). The coordinates of the center of the feature candidate in the preprocessed stimulus image are indicated by *x*_*b*_ and *y*_*b*_. To account for the position invariance of IT cells [21,22], we conducted the max operation for *x*_*b*_, *y*_*b*_, for each band in the set *S*_*k*_. Then, to account for scale invariance [23], we applied the max operation to the consecutive bands defined by *S*_*k*_. The calculation of feature-based responses was also conducted using CNS [19].

Please note that we omitted presenting three color matrices in figures in case the relative weight, *α*, equal to one since they do not contribute to the calculation of the feature-based response (Eq (2)). There were no cases that the best feature with *α* = 0.

### Searching for features with the highest correlation coefficient (step III)

**Fig 4:**
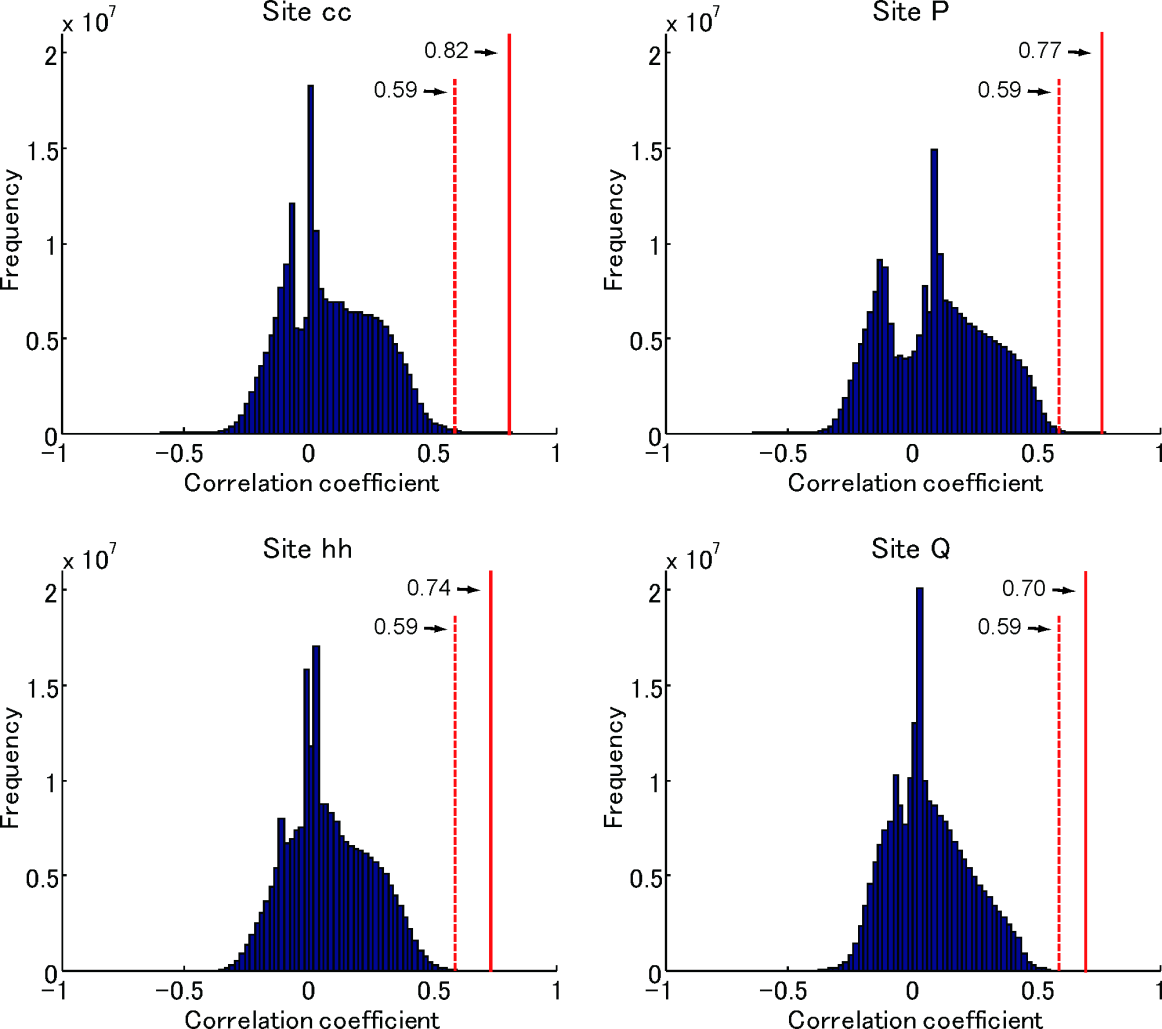
Frequency distribution of feature candidates for determining the value of c.c. in four representative sites. The red line and number with an arrow in each panel indicates the c.c. with the best feature candidate. The red dotted line and number with an arrow in each panel indicates the correlation coefficient that gives significant correlation coefficient (*p* = 0.01).

For each feature candidate, we calculated responses to all stimulus images and constructed a “feature-based response vector.” Then, for each recording site, we calculated the c.c. between the neural response vector and all feature-based response vectors. As feature candidates essentially comprise a random collection of natural images, only a small fraction of feature candidates revealed significant correlation between neural and feature-based response vectors. For example, among 221,760,000 feature candidates (560,000 fragments × 11 relative weights between orientation and color × 36 consecutive scale bands), only 479,792 (0.0021%), 427,762 (0.0019%), 82,809 (0.00037%), and 36,032 (0.00016%) feature candidates had significant correlations with the neural response vector for sites cc, P, hh, and Q, respectively (Fig 4; *p* < 0.01, Bonferroni-corrected). We made the quantitative calculation for 16 (H1), 19 (H2), and 16 (H3), and the median values of the number of feature candidates with significant correlation was 2,112 (IQR 418–34,849), 3287 (IQR76.5–4842.5), and 6.64 × 10^7^(IQR 5.08 × 10^7^–6.81 × 10^7^), respectively. In the case of H3, the number of fragment with significant correlation was larger than H1 and H2. This is because the value of correlation coefficient with *p* < 0.01 depends on the number of stimuli.

Since our interest was on the features with the highest correlation with the neural response vector, among those feature candidates with significant correlation, we selected top hundred feature-candidates for further analyses.

First, to confirm that the obtained high correlation coefficient was not due to outliers of vector elements, we employed delete-half jackknife resampling [17] and calculated Pearson’s correlation coefficient between resampled neural and feature-based response vectors for each feature candidate. Delete-half jackknife resampling is a method for obtaining the sampling distribution of an estimator (Pearson’s correlation coefficient) by taking half of a sample (here, pairs of neural and feature-based response vectors) and calculating the estimate iteratively.

We statistically evaluated significant difference of the mean correlation coefficient between feature candidates (Welch's t-test, *p* < 0.01) using the distribution of correlation coefficient of resampled response vectors (although the normality of the distribution did not meet the criteria of Kolmogolov-Smirnov test, the large number of samples (> = 200) justified the use of t-test). The number of iteration was increased until when we found the single best feature with the mean correlation coefficient significantly higher than those of the others. We evaluated significant differences every 200 iterations of resampling. For the sites where the number of the best features with no significant difference did not decrease for additional 1,000 iterations, we quit resampling and assigned all of these features as the best. The total number of iterations was different from site to site and the range was from 400 to 7,200.

In practice, to save calculation time, we divided the stimulus images into ten segments based on response magnitude and sampled a half number of images from each segment (stratified resampling) instead of employing entirely random resampling. We compared the distribution of correlation coefficients between the random and stratified resampling for some of the recording sites (sites cc, hh, and P) with 10,000 iterations and the difference in the mean correlation coefficient of the best feature was smaller than 1%: the mean values of the correlation coefficients obtained using random and stratified resampling, respectively, were 0.813 and 0.816 (site cc), 0.733 and 0.738 (site hh), and 0.767 and 0.769 (site P). Then, we compared the distributions of c.c.’s for feature candidates and the best feature, namely, the feature candidate with the maximum mean c.c.. Multiple feature candidates tended to have distribution of c.c. similar to each other; therefore, we analyzed all feature candidates for which the mean c.c. is not significantly different from that of the candidate with the maximum value (Welch's t-test, *p* < 0.01).

Second, to examine whether our search method extracted features specific to neural response vectors regardless of the high degree of freedom in our search, we generated artificial response vectors by shuffling stimulus images against neural responses (*n* = 100) and statistically evaluated whether we could find features as good as those for neural response vectors (stimulus-shuffling test). The null hypothesis of this test was that the distribution of the correlation coefficient of the best feature for the neural response vector is the same as the distribution of the correlation coefficients of the best features for the artificial response vectors.

### Additional sets for feature candidates

**Fig 5:**
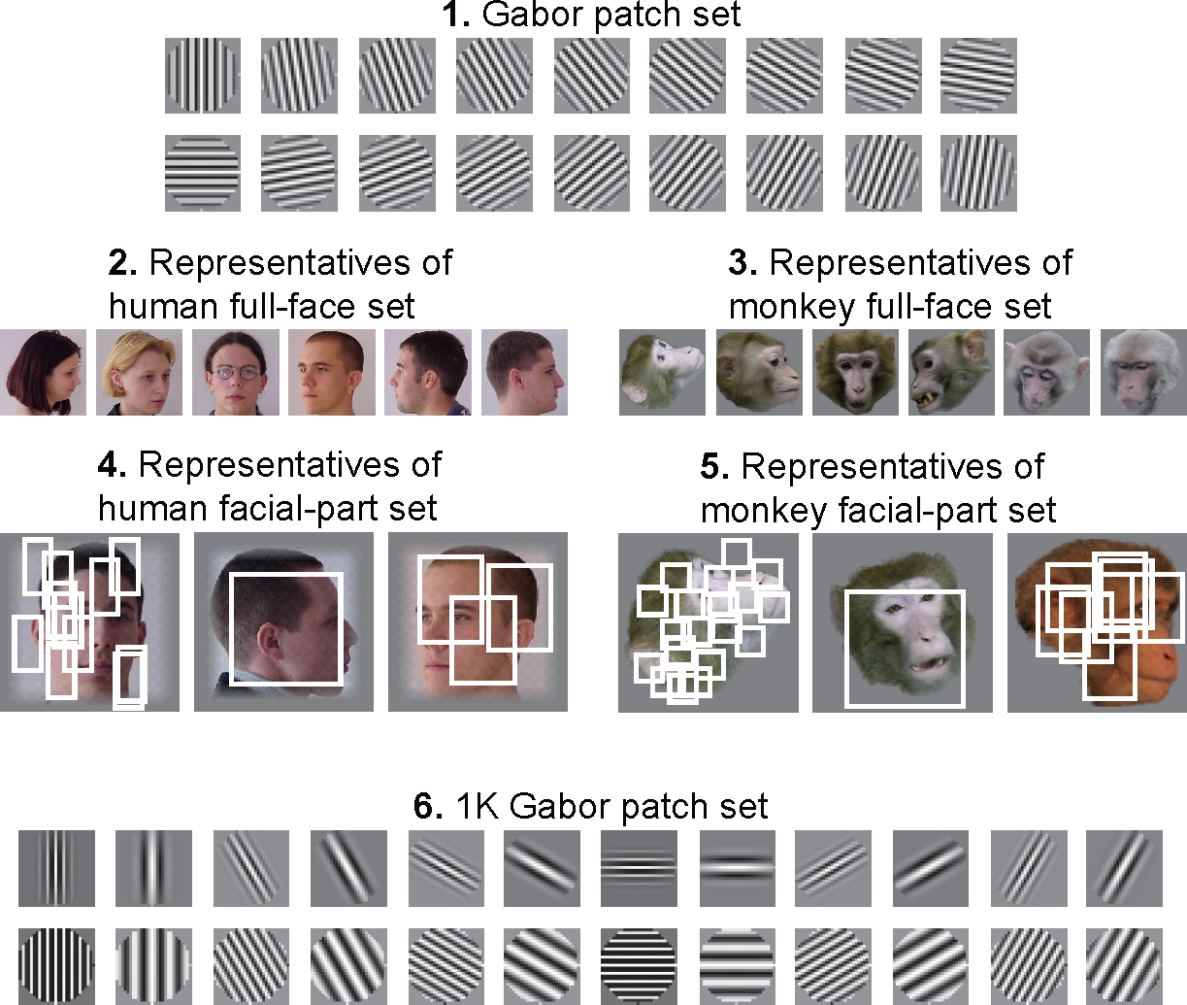
Representative fragments in five additional feature candidate sets. (1) Gabor patch set (*n* = 18) (2) human full-face set (*n* = 570) (3) monkey full-face set (*n* = 764) (4) human facial-part set (*n* = 56,000) (5) monkey facial-part set (*n* = 56,000) (6) 1K Gabor patch set (*n* = 1,080) White rectangles in (4) and (5) show regions chosen as feature candidates. To increase the variety of feature candidates, we allowed partial overlapping of feature candidates in the facial-part sets.

To address fragment set dependency on feature identification, we prepared five additional sets for feature candidates (Fig 5).

#### Gabor patch set

The Gabor patch set comprised feature candidates based on 18 Gabor patch source images, as defined by Eq (1), produced at a 28 × 28 pixel filter size with *α* = 2.8, *λ* = 3.5, and *θ* = 0,10,20,⋯,170 deg. (Fig 5, panel 1). The top left squares (2 × 2) of the Band 1 images (3 × 3) of the 18 Gabor patches were used as the feature candidates.

#### Full-face sets

The human and monkey full-face sets comprised feature candidates whose source images were human (*n* = 570) and monkey (*n* = 764) whole faces taken respectively from the CVL Face [24] and PrimFace databases (Fig 5, panels 2 and 3). The face images were cropped into square regions containing only heads and resized to 200 × 200 pixels, as was done in the facial-part set construction, with the entire Band 1 images, which were in the shape of 46 × 46 pixel squares, used as feature candidates.

#### Facial-part sets

Feature candidates of human and monkey facial-part sets were extracted from source images of 570 human faces taken from the CVL Face Database and of 764 monkey faces taken from the PrimFace database, respectively. The face images were cropped into square regions containing only heads and resized to 200 × 200 pixels using bicubic interpolation. As in the standard set, we extracted features from the overall Band 1 face images and produced 28 feature size-and-shape types. To increase feature variety, we allowed partial overlap among features (Fig 5, panels 4 and 5). For each set, 2,000 features of each type were produced, resulting in a total of 56,000 features, each for the human and monkey facial-part sets.

#### 1K Gabor patch set

The 1K Gabor patch set comprised feature candidates based on 1,080 Gabor patch source images, as defined by Eq (1), produced at a 28 × 28 pixel filter size with *σ* = 0.8,1.8,2.8,3.8,4.8, *λ* = 1.5,2.5,3.5,4.5,5.5,6.5, and *θ* = 0,5,10,⋯,175 deg. (Fig 5, panel 6). The top left squares (2 × 2) of the Band 1 images (3 × 3) of the 1,080 Gabor patches were used as the feature candidates.

## Results

**Fig 6:**
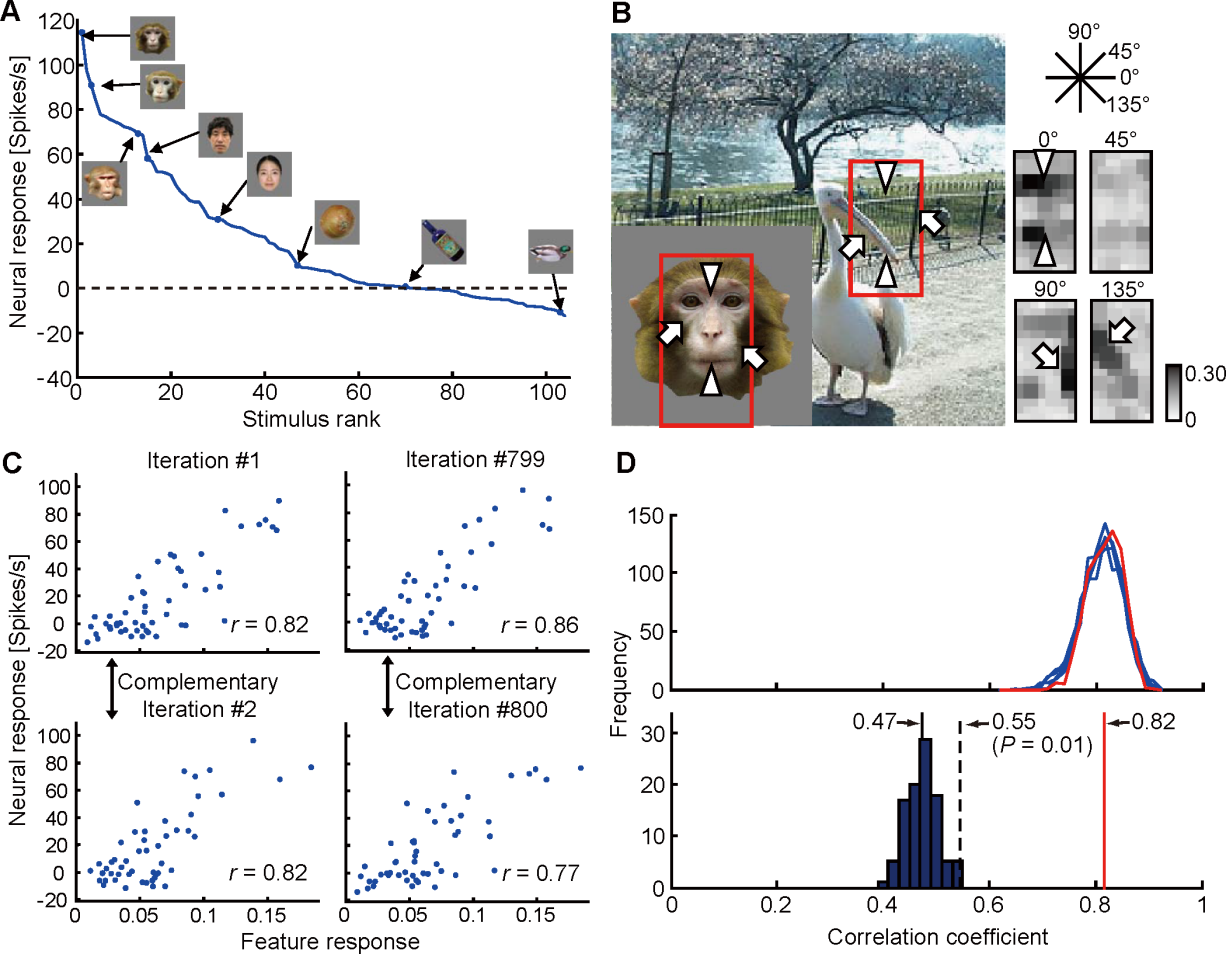
Identification and verification of the best feature for site cc (H1). (**A**) The object response tuning curve (Insets, representative objects). The site preferred monkey to human faces on top of a general preference for faces over non-face objects. Horizontal axis shows stimulus images ranked in order of stimulus-evoked responses. Vertical axis shows stimulus-evoked responses of the site. The horizontal broken line indicates no evoked responses. (**B**) The best feature for site cc (right) and a natural image from which the feature was extracted (left). The feature was extracted from the outlined region in the natural image and was characterized by four edges (arrows and arrow heads) and no color information (*α* = 1). Please note that the arrows and arrow heads in the left and right panels point to corresponding spatial positions. The left inset shows the face stimulus that evoked the strongest neural response. The red contour indicates the region for which the feature gave the maximum response within the face stimulus. Gabor filter angles are given in the upper right corner. (**C**) Scattergrams between neural and feature-based responses for subsampled stimulus images (52 among 104 objects). Upper and lower panels are complementary stimulus sets. Data points represent different stimuli. There was a significant correlation between neural and feature-based responses for all four iterations (inset, c.c., *r*; t-test for Pearson's c.c., *p* < 0.0001). (**D**) (Upper panel) distributions of c.c. for 800 iterations of the delete-half jackknife resampling. The distributions of the top five features among 560,000 candidates are given as representatives. The distribution of the best feature (red) has a mean value statistically significantly different from the mean values of the other distributions (blue) (mean ± s.d. of the best, 0.82 ± 0.03; Welch's t-test, *p* = 0.0034). (Lower panel) Results of the stimulus-shuffling test showing distribution of c.c.’s of the best features identified for artificial response vectors generated by shuffling stimulus images against neural responses (mean ± s.d., 0.47 ± 0.03; *n* = 100). The broken line indicates the value of c.c. with *p* = 0.01 (c.c., 0.55). The red line indicates the mean c.c. of the best feature for the neural response vectors.

**Fig 7:**
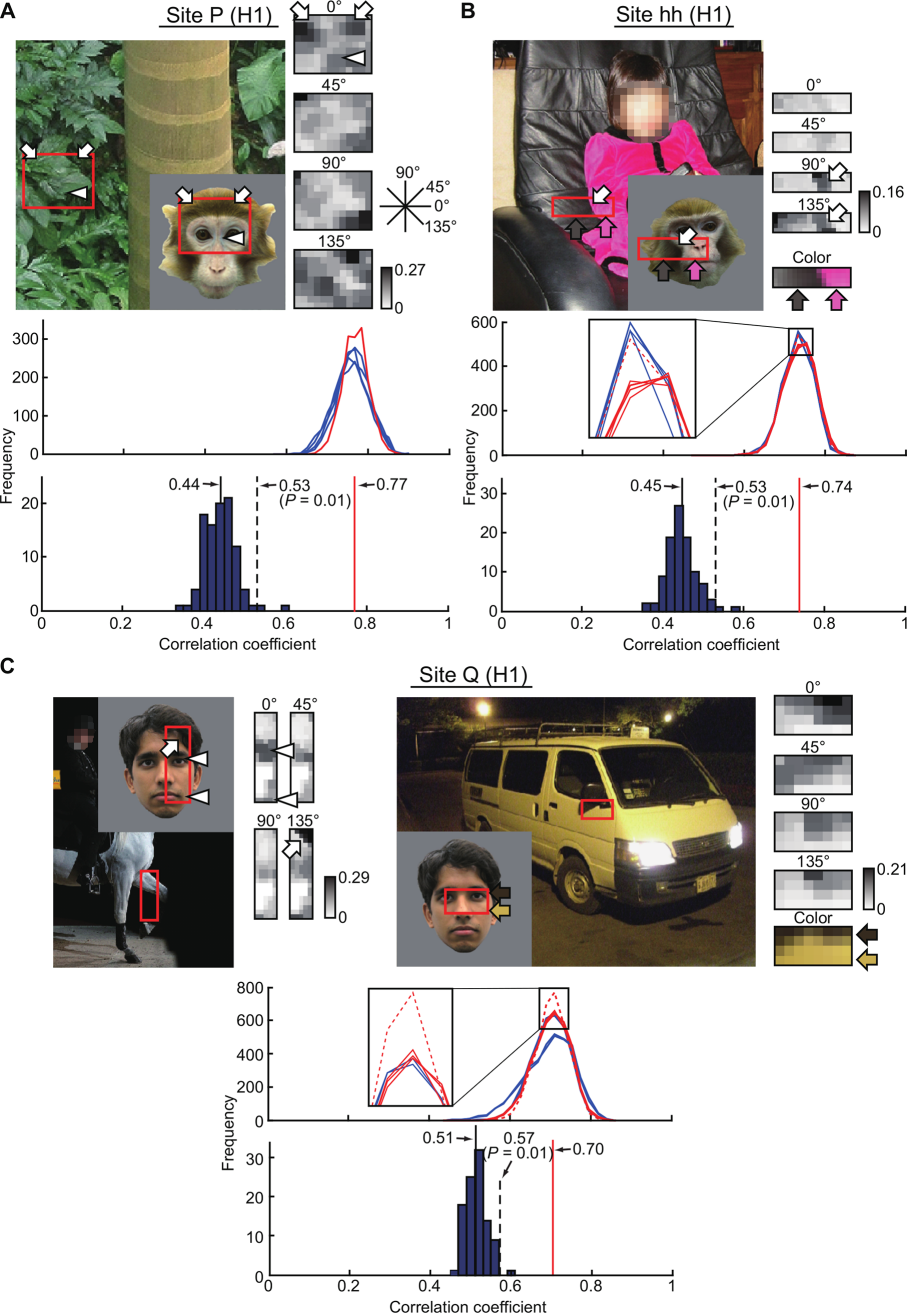
The best features for three other representative sites: P, hh, and Q (H1). Conventions are the same as in Fig 6B and 6D. (**A**) The best feature for site P (mean ± s.d., 0.77 ± 0.03). The mean value of c.c. of the best feature is significantly different from the other feature candidates (Welch's t-test, *p* = 0.0068). The c.c. of the best feature for artificial response vectors is 0.44 ± 0.03 (mean ± s.d.; *n* = 100). (**B**) Identified features for site hh. Seven features are not significantly different in terms of mean c.c. value from the best (distributions in red; mean ± s.d., 0.74 ± 0.03). These were derived from the same region of a natural image (upper left) and are characterized by local orientations (upper right four matrices from the top) and color (fifth matrix from the top in upper right). While those in red solid lines differ over a consecutive scale range, the feature indicated with red broken line differs in terms of color channel contribution (with *α* = 0.5 for the indicated feature versus 0.6 for the others). The distributions of four features that are significantly different from the best are shown in blue (Welch's t-test, *p* = 0.0004). Some of the distributions shown in red overlap and are indistinguishable in this panel. The c.c. of the best feature for artificial response vectors is 0.45 ± 0.03 (mean ± s.d.; *n* = 100). (**C**) Identified features for site Q. The best feature originated from a natural image in the upper left and represents a combination of local orientations (mean ± s.d., 0.70 ± 0.04; red broken line). The other three features do not differ significantly in terms of mean c.c. value from the best (red solid line). They originated from a natural image different from the best and are composed of orientation and color channel components (*α* = 0.8) (upper right). The c.c. distributions of the identified features differ significantly from the distributions of the others (blue) (Welch's t-test, *p* = 0.0022). The c.c. of the best feature for artificial response vectors is 0.51 ± 0.02 (mean ± s.d.; *n* = 100).

We summarize the step-by-step results of the fragment-based approach for representative sites, cc, P, hh, and Q (Figs 6 and 7). The neural response vector of site cc was characterized by a response tuning curve in Fig 6A, and the feature with the highest c.c. with the neural response vector is shown in Fig 6B. The scattergrams (Fig 6C) revealed a close correlation between neural and feature-based responses for subsampled stimulus sets. In particular, the correlation coefficients were almost the same for the subsampled sets where no overlapping samples across the sets (complementary sets) (iterations #1 and #2, and iterations #799 and #800). The distributions of c.c. of all subsampled stimulus sets for top five feature candidates revealed a significant correlation between the neural and feature-based response vectors regardless of sampled stimuli (800 iterations; *p* < 0.0001), meaning that correlation between neural and feature-based responses was not due to outliers within the stimulus images (Fig 6D, upper panel). The mean c.c. of the best feature (0.82) was statistically significantly different from that for all other feature candidates. Similarly, we identified one unique feature in site P (Fig 7A). In site hh (H1), however, seven features did not significantly differ from the best in terms of mean c.c. values (Fig 7B). These features differed in terms of scale-ranges (inset, solid red lines) or *α* values (inset, the broken red line), but originated from the same region of a natural image. Furthermore, features with different scale-ranges shared a common scale band. Therefore, these features are structurally similar. In fact, correlation of neural response vectors between the best feature and seven features was 0.998 ± 0.002 (mean ± SD). For simplicity, we regarded the features originating from the same region of a natural image as one feature. Based on this, we identified one feature as site cc (H1), hh (H1), or P (H1) for 18 out of 35 sites (H1 and H2). There were also sites where features originated from different regions of natural images had no significant differences from the best (Fig 7C; site Q). In site Q, for example, we found the best feature (upper left) whose mean c.c. value was 0.70 (the distribution of c.c. was indicated by the broken red line). There were three other features whose mean c.c. values (solid red lines) were not significantly different from the value for the best feature. These three features originated from the same region of a natural image (upper right) with different scale ranges; by definition, three features are considered as a feature. The correlation of neural response vectors among three features was 0.999, while correlation of neural response vectors between the best feature in Fig 7C left and the feature in Fig 7C right with maximum c.c. was 0.716. Therefore, we identified two features (Fig 7C, left and right) in site Q. For 17 sites among 35 sites (H1 and H2), we identified multiple features derived from different natural images as site Q (H1). Even though the regions where features are derived from were different, these features may share the same structure. Alternatively, they may represent subsets of neurons within a recording site representing different features or multiple feature representation by a single cell, as suggested by a previous study [12]. Since we do not have unambiguous ways to quantify similarity among features, it remains for a future study to address which is the case.

### Specificity of the search method to neural response vectors

The stimulus-shuffling test revealed that none of the best features obtained for artificial response vectors reached the c.c. of the best feature for the neural response vectors (Figs 6D and 7, bottom of each panel), indicating that our search method is specific to response vectors derived from neurons. In 32 of 35 sites, the c.c. of the best feature was significantly larger than that for the artificial response vector (Z-test, *p* < 0.01).

### Generalization to the novel stimulus images

**Fig 8:**
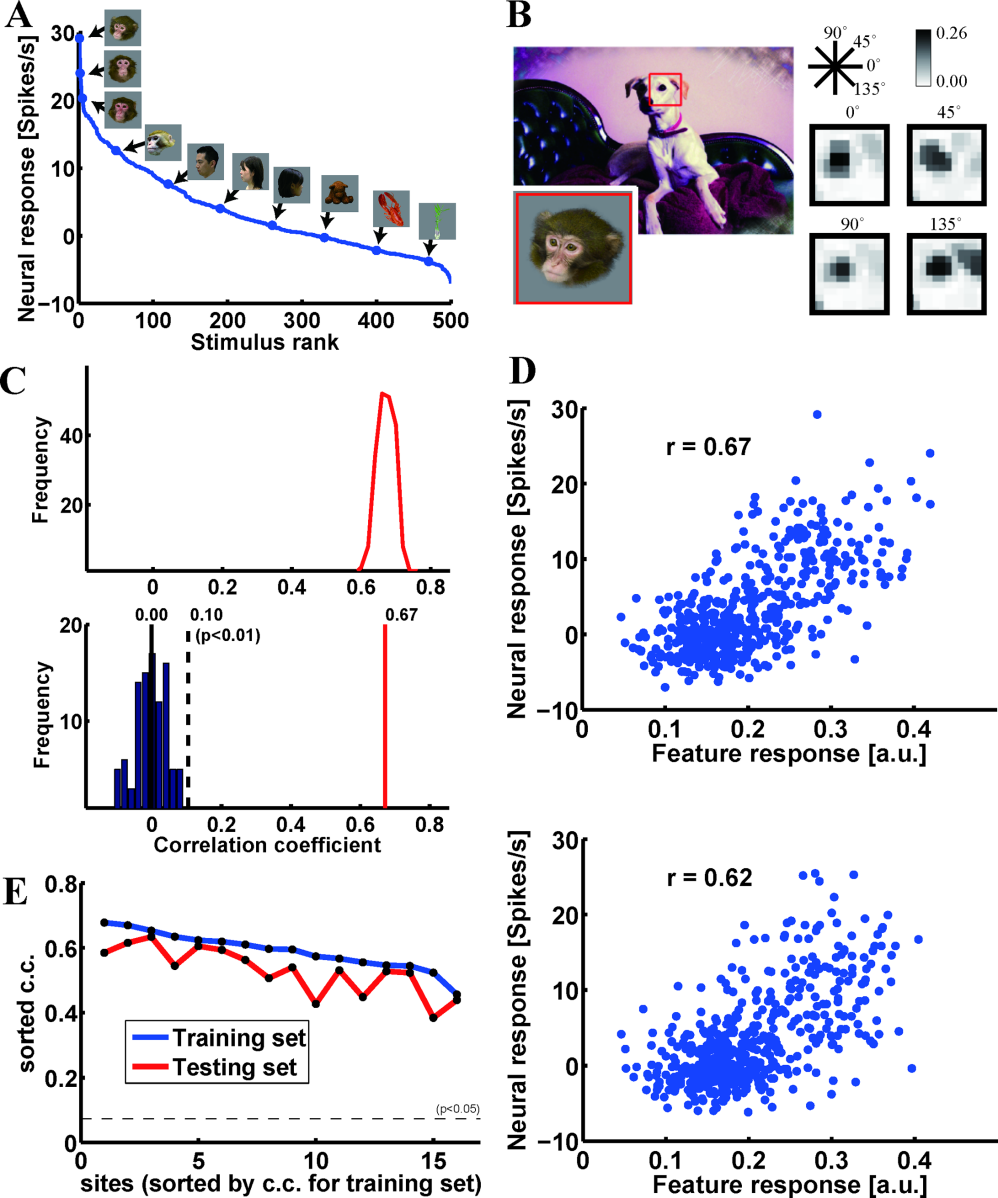
Identification and verification of the best feature for a representative site from which responses to 1,000 stimulus images were recorded. (**A**) The object response tuning curve shows characteristics of the neural response vector of site M. (**B**) The best feature for site M (H3). The image fragment from which the best feature was derived is outlined in red. The feature was matched to the entire stimulus image, where the upper-left part of the feature was matched to complex shapes around the eyes of the monkey, and the upper-right part of the feature was matched to the edge between the monkey face and background at the same area in the stimulus image. The best feature does not require color channels (*α* = 1.0). Conventions are the same as in Fig 6B. (**C**) (Upper panel) Distribution of correlation coefficients for 200 delete-half jackknife iterations for the best feature (mean ± s.d., 0.67 ± 0.03). Please note that the standard deviation of the distribution is smaller than in the cases shown in Figs 6 and 7 because of the larger number of stimulus images. **(**Lower panel) The distribution of the correlation coefficient for 100 artificial response vectors generated by shuffling stimulus images against neural responses (mean ± s.d., 0.00 ± 0.05). The red line indicates the average of the correlation coefficients of the 200 delete-half jackknife iterations for the best feature. (D) Scattergrams between the neural and feature-based responses for the training (upper) and test (lower) set. (E) The c.c. value for the training and test set for all examined sites (*n* = 16).

The scattergrams (Fig 6C) show that the best feature candidate for a half of 104 stimuli was also good for no-overlapping other half of stimuli. However, considering feature complexity, the number of stimuli might not be enough to evaluate generalization performance. To further examine generalization performance, we examined the physiological dataset obtained from H3, where neural responses were recorded for 1,000 object stimuli. We used randomly chosen 500 of them (training set) to search for the best feature as in Figs 6 and 7 (Fig 8). In the representative site M from H3, the c.c. between neural and feature-based response vectors for the best feature was 0.67, while c.c. for the best feature obtained with artificial response vectors was 0.00 ± 0.05. This number is even smaller than what we obtained in Figs 6 and 7. This is due to increasing the number of stimuli. Please note that we did not find such big changes in c.c. between neural response and feature-based response vectors. With this feature, the c.c. between fragment and neural response vectors for unused 500 stimuli (test set) was 0.62. The value is not largely different from c.c. value for the training set, suggesting that performance we obtained for the training set can be generalized to novel stimuli. The scattergrams between neural and feature-based response vectors are given in D (Fig 8). Although we did not apply delete-half jackknife resampling for the test set, distributions suggest that it is less possible that the observed c.c. was due to outliers within the stimulus images. Fig 8E revealed c.c. of training and test sets for examined 16 sites. The mean c.c. and standard deviation across 16 sites was 0.59 ± 0.06 for the training set and 0.53 ± 0.07 for the test set, providing consistent results with site M.

### Potential biases owing to the sources of feature candidates

**Fig 9:**
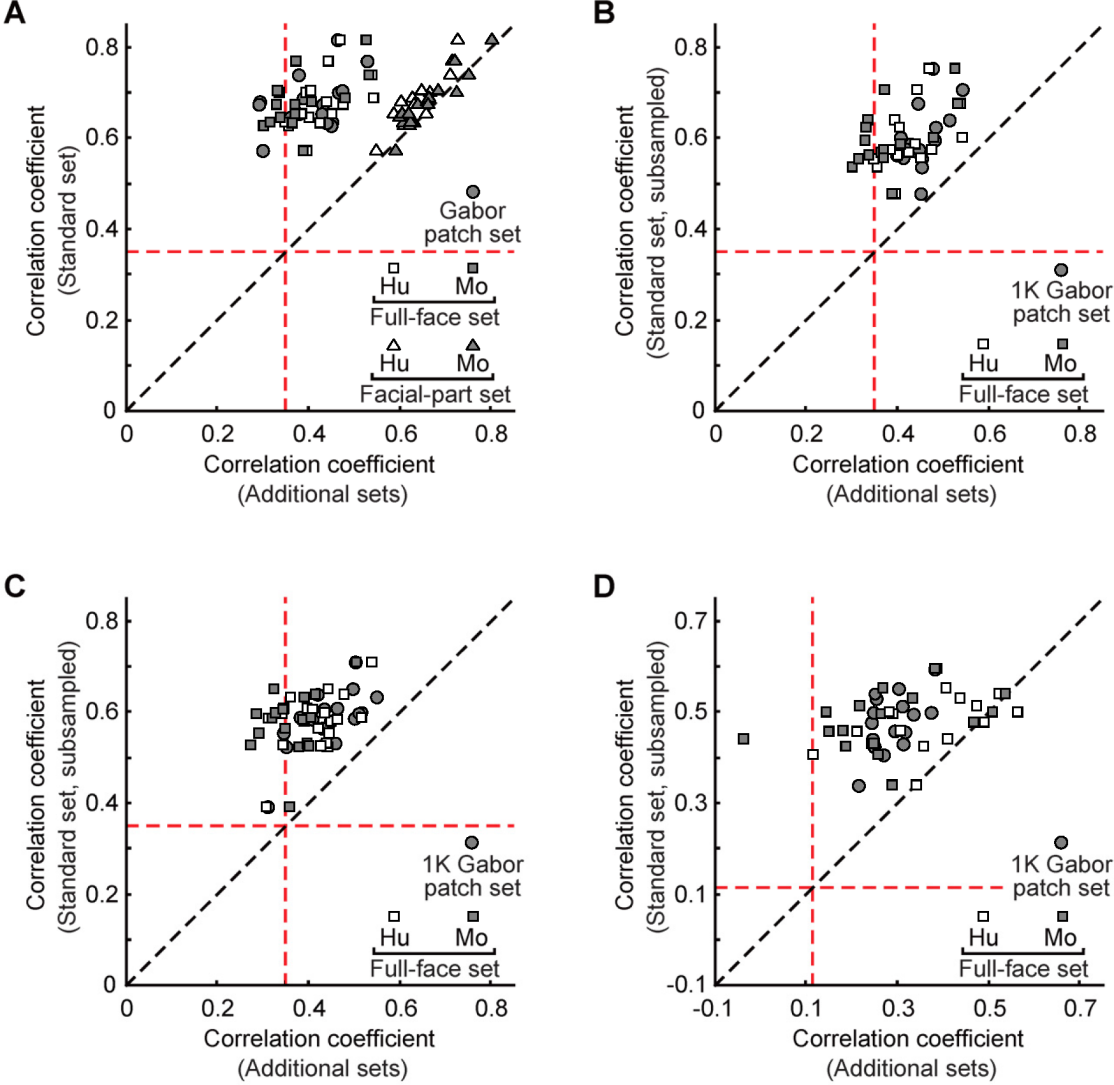
Comparison between c.c. values for the best features extracted from the standard set and those extracted from the other sets. Different symbols represent different fragment sets, while different points with the same symbol represent different sites (*n* = 16 for H1 and H3; *n* = 19 for H2). The red lines parallel to the horizontal and vertical axes indicate the correlation coefficient that gives significant correlation coefficient (c.c. = 0.35 for A-C, 0.12 for D; *p* = 0.01). (**A**) The result from H1. The number of feature candidates are 560000, 18, 570, and 764 for the standard set, Gabor patch set, human full-face set, and monkey full-face set, respectively (**B-D**) The number of feature candidates are 560, 1080, 570, and 764 for the standard set, 1K Gabor patch set, human full-face set, and monkey full-face set, respectively. For the standard set, feature candidates (*n* = 560) were randomly subsampled from the standard set consisting of 560,000 candidates. The c.c. value for the best fragment (vertical axis) gives average of 10 different subsampled sets. The result from H1, H2, and H3 are given in B, C, and D, respectively. (C, D) The results from H2 (C) and H3 (D). Please note that in H3 (D), the neural response vectors are divided into training and test sets, and the result from the test set is given as in Fig 8.

To test potential biases owing to feature candidate sets, instead of extractions from natural images, we examined for 16 recording sites (H1) preprocessed Gabor patches, full-faces, and facial parts as feature candidates (Fig 5). To increase feature variety, we allowed facial-part feature candidates to overlap. As shown in Fig 9A, the c.c. for the best feature was far below those derived from the standard set for all 16 sites when the Gabor patch (indicated by circles in the figure) or full-face set (squares) were used as the source of features (left to the line of equality in the figure). Unlike the best features extracted from the standard set, the best Gabor patch and full-face features did not even show significant correlation between neural and feature-based response vectors for some sites (t-test for Pearson’s c.c., *p* > 0.05). This result with Gabor patch and full-face sets may be due to imbalance in the number of feature candidates among the sets. To test this possibility, we made an additional analysis where the numbers of candidates were set to 560, 1080, 570, and 764 for the standard set (subsampled), 1K Gabor patch set, human full-face set, and monkey full-face set, respectively (Fig 9B–9D). In H1 (Fig 9B) and H2 (Fig 9C), we found that the best features obtained from both 1K Gabor patches and facial parts had still lower scores than those of the best feature derived from the standard set (symbols representing individual sites were located left side of the orthogonal line). In the case of H3, we divided neural response vectors into training and test sets as in Fig 8. The correlation coefficient for the test set revealed the same tendency as in H1 and H2 except for a small number of sites (Fig 9D).

These results reveal that the simplest and most complex features—Gabor patches and faces, respectively—cannot be explanatory features for individual sites in the face domains.

On the other hand, the best features extracted from the facial parts (facial-part set) produced c.c.’s nearly identical to those from the standard set (triangle symbols in the figure). However, they were never better than those from the standard set. Furthermore, while the standard set included faces, the identified features from the standard originated from faces for only three out of 35 sites (Fig 1). Thus, using the correlation between neural and feature-based response vectors as the criterion to evaluate features allows good features to be selected from non-face images, suggesting that a sufficiently large set of natural image fragments can provide a library of features that can be used in the representation of various object categories.

### Characterization of features represented by columns in the face domains

**Fig 10:**
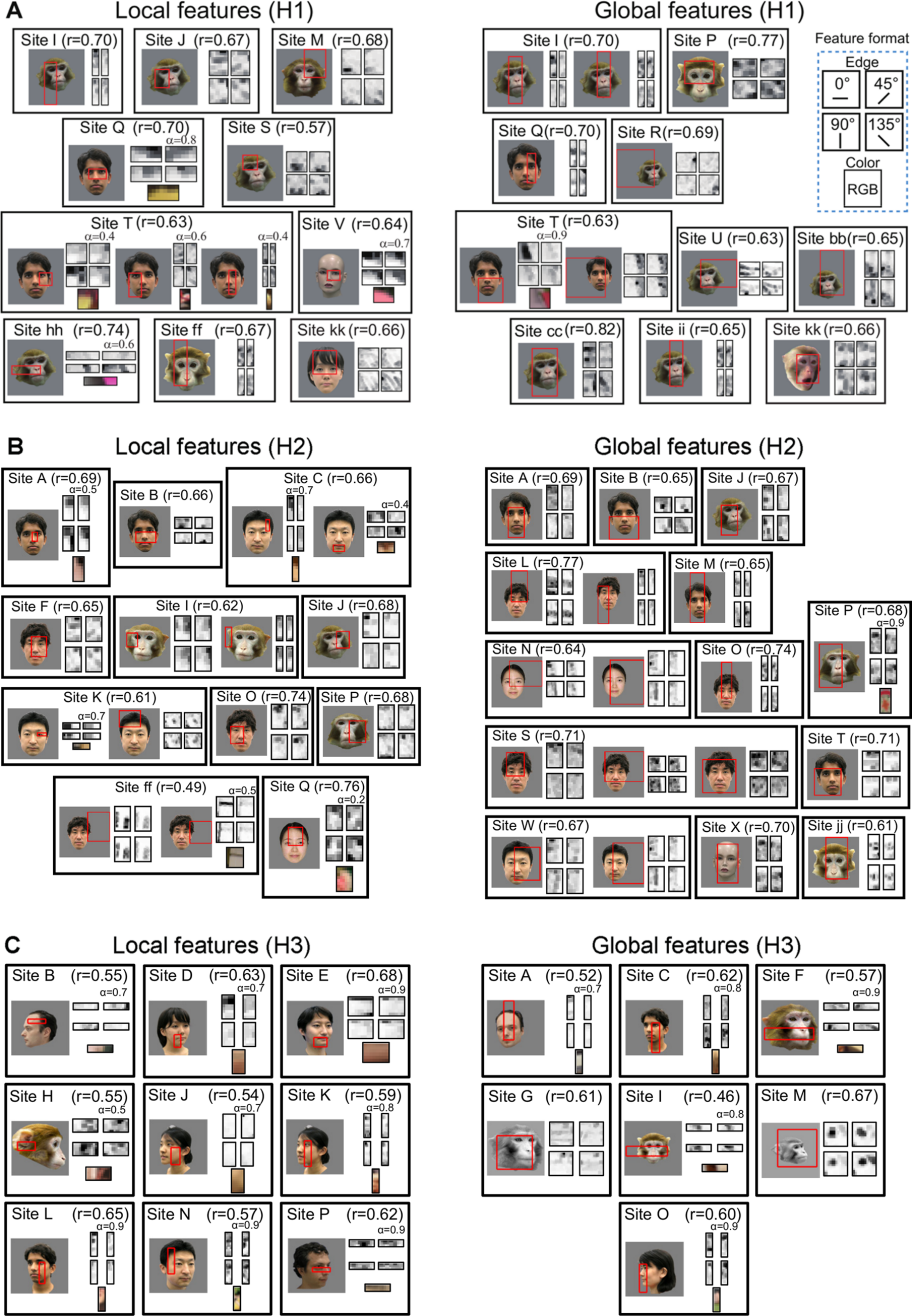
Columns in the face domains detect global and local structures of faces. The best fragments and the region within the best face stimulus where the fragments had the best match (red rectangles) are indicated for 16 sites in H1 (A), 19 sites in H2 (B) and 16 sites in H3 (C). The format of the feature representation is given in upper right (box with broken line in blue). In cases in which color information is critical, the relative weight between orientation and color channels, *α*, is indicated. Multiple fragments are represented in some sites such as Q in which multiple fragments do not significantly differ. Fragments detecting a local part and those detecting the global structure are grouped by eye and shown separately in left and right. The sites where both fragments with a local part and the global structure were detected were shown in both left and right.

**Fig 11:**
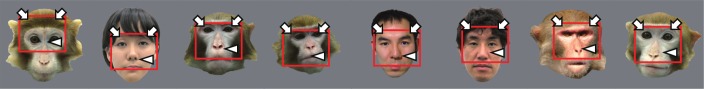
Site P detects a top-heavy configuration of faces typically consisting of two eyes and a nose below. The pictures represent eight faces that evoked the best (left) to eighth best (right) neural responses in site P. The red contour indicates the region for which the feature gave the maximum response, and arrows and an arrowhead point critical points of the feature (see Fig 7A).

The feature identified by the fragment-based approach revealed unique aspects of features of columns in the face domains. Generally, the combinations of local orientation and colors were relatively complex and differed among sites (Fig 10A). Inspection of the locations in the stimulus images at which the identified feature provides maximum similarity revealed that some of the best features detected local part of faces, including sites M, S, V, and hh, while other features captured global structures of faces such as sites cc, P, and ii. The global structures comprise a facial configuration and local features of faces. In site cc, for example, the identified feature contains long and short horizontal orientations (arrow heads) derived from horizontal fence bars (Fig 6B). The long and short horizontal orientations are well matched to horizontal dark regions derived from the two eyes and mouth (arrow heads) in a monkey face (Inset to the left). In addition, the feature of site cc also contains two oblique orientations (arrows) derived from a pelican beak and a vertical fence bar in the natural image (Fig 6B). These local features cause this site to respond differently to monkey and human faces (Fig 6A). The identified feature in site P contains three clusters of local horizontal orientations arranged in a top-heavy configuration. In the best face stimulus, the feature detects structure of left and right side of the hair line covering the head and nose line (Fig 7A). Interestingly, inspection of top eight face stimuli revealed that the feature tended to detect the two eyes and nose in a face (Fig 11). Similar to site cc, the feature of site P contains vertical and 45 degree orientations at the upper left and lower right, respectively, causing the site to respond to faces with particular sets of facial lines (Fig 7A). Finally, in site Q (Fig 7C), the feature in the left represents a specific hair line (arrow) in addition to an eye and mouth representation (arrow heads). Altogether, the features that captured global structures of faces concurrently represented feature elements arranged in facial configuration and those differentiating faces. In the same way, we visualized the fragments and features obtained from H2 and H3 according to their coverage in Fig 10B and 10C, respectively.

**Fig 12:**
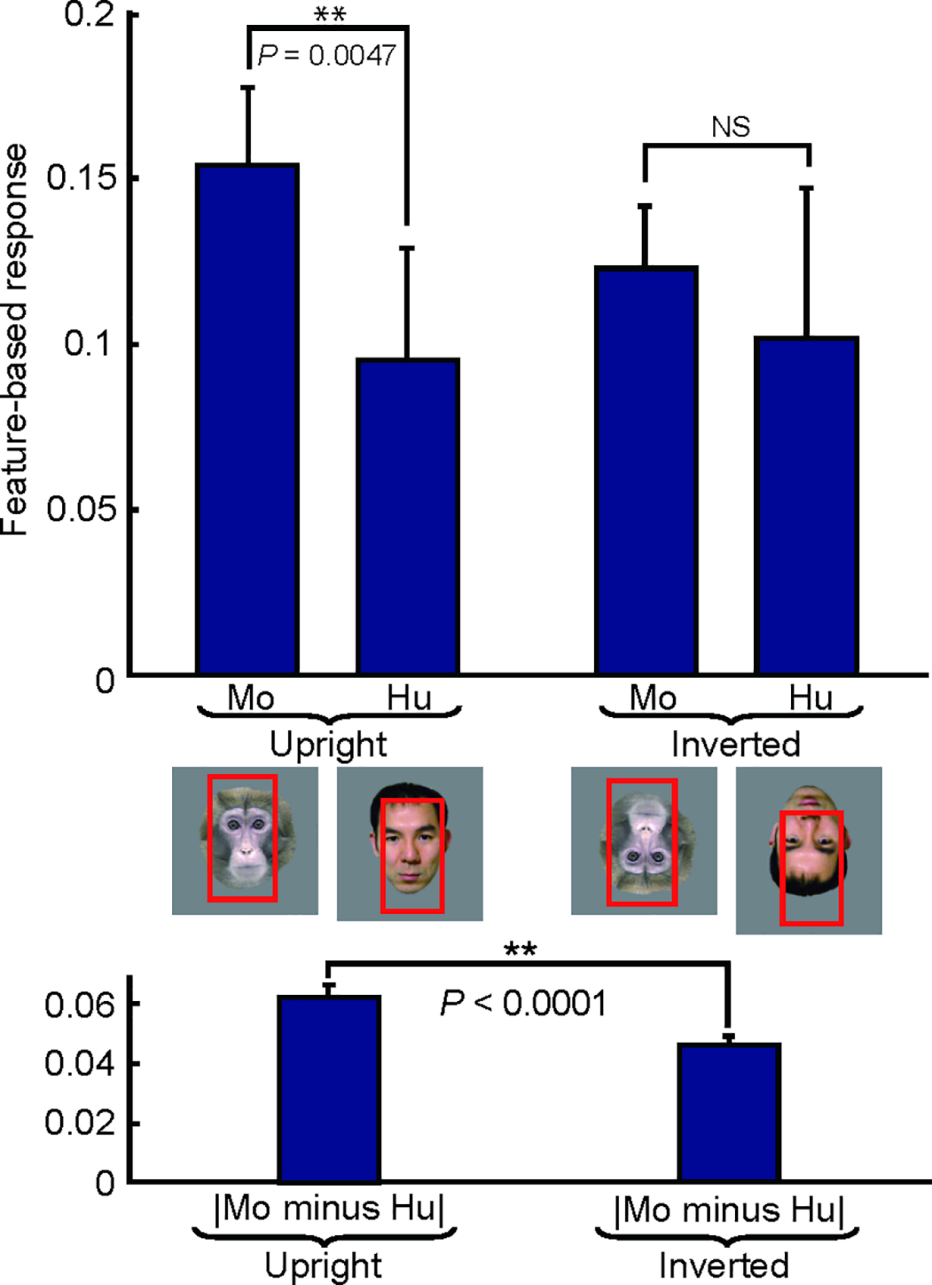
Predicted responses to upright and inverted faces provide a possible explanation of inversion effects. Upper panel, mean feature-based responses to eight monkey (Mo) and eight human (Hu) faces in upright and inverted conditions for site cc (H1). Error bars indicate standard deviation. Responsive regions in the representative faces are given below in red. Lower panel, average of absolute differences between responses to monkey and human faces in upright (left) and inverted conditions (right). Error bars indicate the standard error of the mean. The numbers indicate p-values (Wilcoxon signed-rank test).

These unique arrangements of local elements in these features may provide a neural basis for face inversion effects in which recognition of faces with subtle differences is lost or degraded when they are inverted [25,26]. To test this possibility, we calculated feature-based responses to upright and inverted faces in 19 sites for which the upright face responses differ significantly between humans and monkey faces (Wilcoxon signed-rank test, *p* < 0.05). We found that the absolute difference in feature-based response between monkey and human faces in the upright condition was statistically significantly larger than those in the inverted condition in 12 of the 19 sites (63.2%, single-sided Wilcoxon signed-rank test, *p* < 0.05) (see Fig 12 for a representative site). Although there could be multiple mechanisms for the face inversion effect, the above quantitative description and response estimation results suggest one possible mechanism. Consistent with this, a recent physiological study examined the responses of face neurons to upright and inverted faces and found that neuronal information about the fine category (facial identity and expression) decreased when faces were inverted [27].

## Discussion

In the present study, we showed that features explaining the response variance of face neurons can be recovered from the set of natural image fragments if the set is sufficiently large. Although the combinatorial number of pixel configurations in the size of the fragments is huge, the combinations in the natural image fragments are of limited complexity. Thus, our search procedure uses the fact that image fragments coming from natural configurations cover only a small part of this large space.

A unique feature of our method is that we can mathematically calculate responses to arbitrary objects according to Eq (2). Correlation coefficient between responses predicted from the recovered features and the object responses of individual sites was very high. For example, the identified feature for site M in H3 explains 38% of the variance in object responses (with a c.c. value of 0.62 in the test set). On average, c.c. values between neural and feature-based response vectors was 0.53 for H3 for the test set.

In the present study, we focused on the features of functional columns. This is because previous studies have suggested that IT cortex is organized in functional columns [12,13]. For example, Sato and colleagues [12] densely recorded object responses of neurons from a putative columnar region and found that object response tuning curves of individual neurons are scattered around the vicinity of a common response tuning curve. They also found that the common response tuning curve of a columnar region is largely different from that of nearby columnar regions. In the present study, we took average of MU responses as the common response tuning curve, and developed the fragment-based approach for the averaged response tuning curves. However, in principle, the method will be applicable to tuning curves of individual neurons. Fragment-based approach for individual neurons as well as columns would enable us to address how responses of individual neurons are related to the columnar response. We identified multiple features that were not visually similar to each other in some sites, such as site Q (Figs 7C and 10). This may indicate subsets of neurons within a recording site representing different features, or multiple feature representation by a single cell, as suggested by a previous study [12]. Fragment-based approach for individual neurons within a column and comparison with the feature of the column remain for future investigations.

Our procedure was corroborated using two statistical tests: delete-half jackknife resampling and stimulus-shuffling. Delete-half jackknife resampling confirmed that the high c.c. values were not biased to partial neural properties or feature-based response vectors (Fig 6C). The stimulus-shuffling test confirmed the specificity of our method to neural response vectors, as the c.c. values of the best features for artificial response vectors were very low and had no significant correlation (Figs 6D and 7). A more conclusive test would be to examine whether identified features activate neurons. Doing so, however, would require reconverting the identified features in preprocessed form back to pixel-based images before showing them to monkeys. As a preprocessed fragment will not produce a unique pixel-based image, such conversion is not mathematically simple. Thus, we are limited to developing a strategy to find features that mimic the response of the recording site reasonably well.

**Fig 13:**
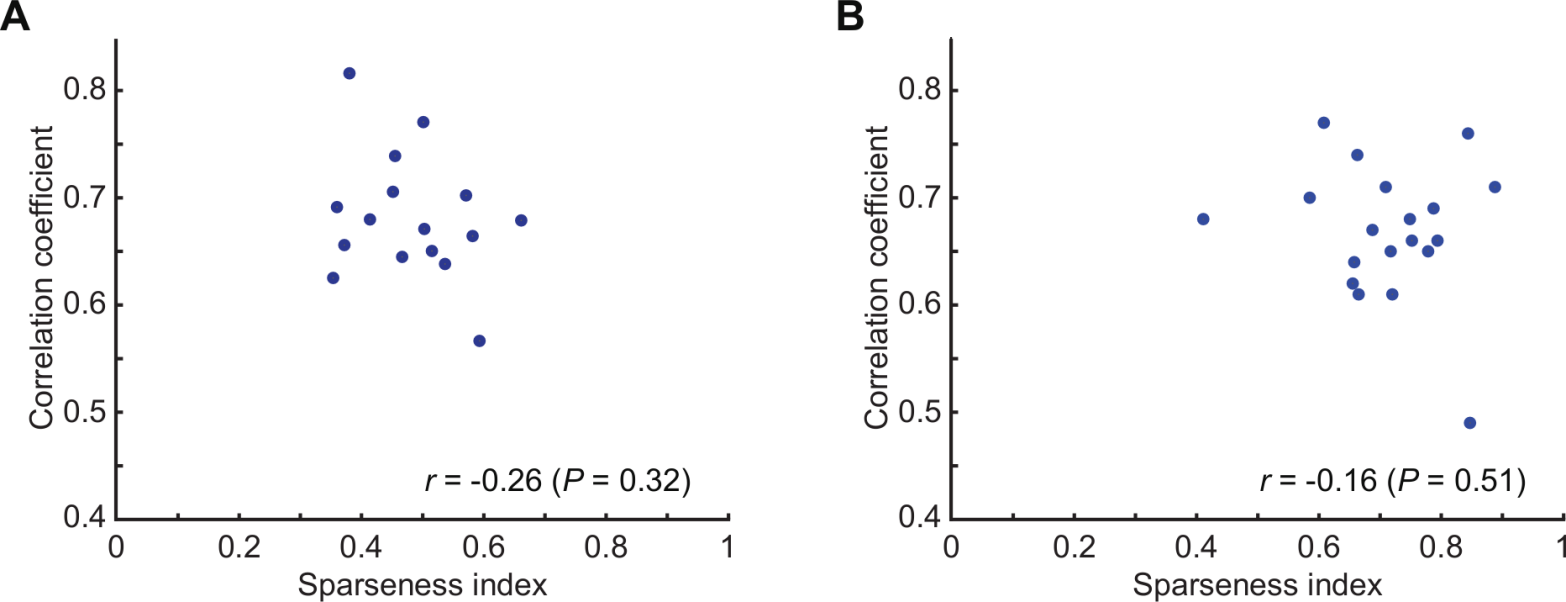
**Scattergram between the sparseness index and c.c. of the best feature for 16 sites in H1 (A) and 19 sites in H2 (B).** Different points represent different sites.

We investigated whether correlation between feature-based and neural response vectors can be affected by the difference in the shapes of neural response tuning curves, for example, sharpness of the tuning curves. To quantify shapes of the tuning curves, we used the sparseness index [28]. The response sparseness is defined as

$$a={\left(\sum_{i=1,n}r_{i}/n\right)}^{2}/\sum_{i=1,n}\left(r_{i}^{2}/n\right)$$

where *r*_*i*_ the firing rate to *i*-th stimulus in the set of *n* stimuli. With regard to the spontaneous firing rate, the negative responses were clipped to 0 [28]. We examined for 16 sites in H1 and 19 sites in H2 where the number of the stimuli were the same. There was no significant correlation between the sparseness indices and c.c. for the best features (correlation coefficient, -0.263 (*p* = 0.324) for H1, and correlation coefficient, -0.162 (*p* = 0.507) for H2) (Fig 13). The results showed that our approach successfully extracts features regardless of whether neurons are broadly or sharply tuned to object stimuli.

Our method seems to be the only one to date that provides features that explain the variance of natural object responses of IT neurons in a reasonably satisfactory manner. Brincat and Connor modeled critical features of IT neurons as linear and nonlinear combinations of curvatures [4]. Their model explained 49% (average c.c. = 0.7) of the variance of responses to artificial stimuli composed of curvatures. Because their model is specific to curvatures, we cannot address how well it explains responses to natural images. With respect to natural images, David and colleagues proposed a model for V4 neurons in terms of the spectral receptive field [10]. This model closely explains previous findings that V4 neurons respond to angled or curved contour features. However, the average c.c. between their model-predicted responses and actual neural responses to natural images was 0.32, meaning that only 10% of the response variance was explained. In general, finding features that explain large variances of neural response to natural images remain difficult and, to our knowledge, there had been no attempt to explain the natural image responses of IT cells prior to this study. Our research reveals that searching for features among natural image fragments is an effective method for overcoming this difficulty.

Our method shares some common aspects with several appearance-based object recognition computer vision algorithms, including SIFT [29], HOG [30], and HMAX [20]. In a manner similar to the preprocessing used in our search method, these algorithms extract local gradient or orientation information with small position invariance at the first stage. In general, low-level feature processing is an essential step in object recognition, and modifying our preprocessing so that no orientation channels are used (*α* = 0) and the color channels were gray-scaled reduces the c.c. between neural and best feature-based response vectors to 0.50 ± 0.06 (mean ± s.d. across 16 sites in H1).

Prior implementation of a fragment-based scheme [31] demonstrated that object recognition requires image fragments of intermediate complexity. In this study, we showed that the best fragments are not as simple as Gabor patches or as complex as entire faces (Fig 9). Previous physiological studies also suggested that IT cells encode features with intermediate complexity [1–4,9,21]. These findings corroborate the results from this study suggesting that features with intermediate complexity are essential in object representation. Considering the complexity of identified features, the number of identified features is still small to draw a picture of general property of features represented by IT columns. Nevertheless, it is noteworthy that completely identical features (Fig 1, natural images with asterisks) could be identified in other sites and in two hemispheres even though there were 560,000 possible candidates: a natural image fragment containing a single asterisk gave the best feature for sites J (H1) and F (H2), while a natural image fragment containing double asterisks gave the best feature for sites ii and ff in H1 and site O in H2 (Fig 1).

The central issue of object vision is where and how objects are represented invariantly. IT cortex is the most plausible cortical area to have the invariant representation. For example, the receptive field of IT neurons is relatively large [21,22]. Since the fragment based approach takes the large receptive field into account (Eq (2)), identified features as well as neurons provide a substrate for position invariance. Previous studies have shown that object images are represented by combinations of neurons in IT cortex and combinations are essential for invariant object representation [2,3,5,6,14]. Therefore, combinations of the features identified for face neurons could explain view invariant face representation. Interaction across cortices may also be involved in the mechanisms of invariant object recognition. For example, previous studies suggested involvement of feedback interaction with earlier visual cortices in figure-ground segregation [32,33]. The remaining problems for future studies are (1) to what extent the combinations of features with the specific arrangement of local orientations and colors explain invariant face representation and (2) in what degree cortico-cortical interaction is required. The present study provides a good platform to address these questions because our fragment-based approach provides features of neurons in a mathematically manipulable form.

**Fig 14:**
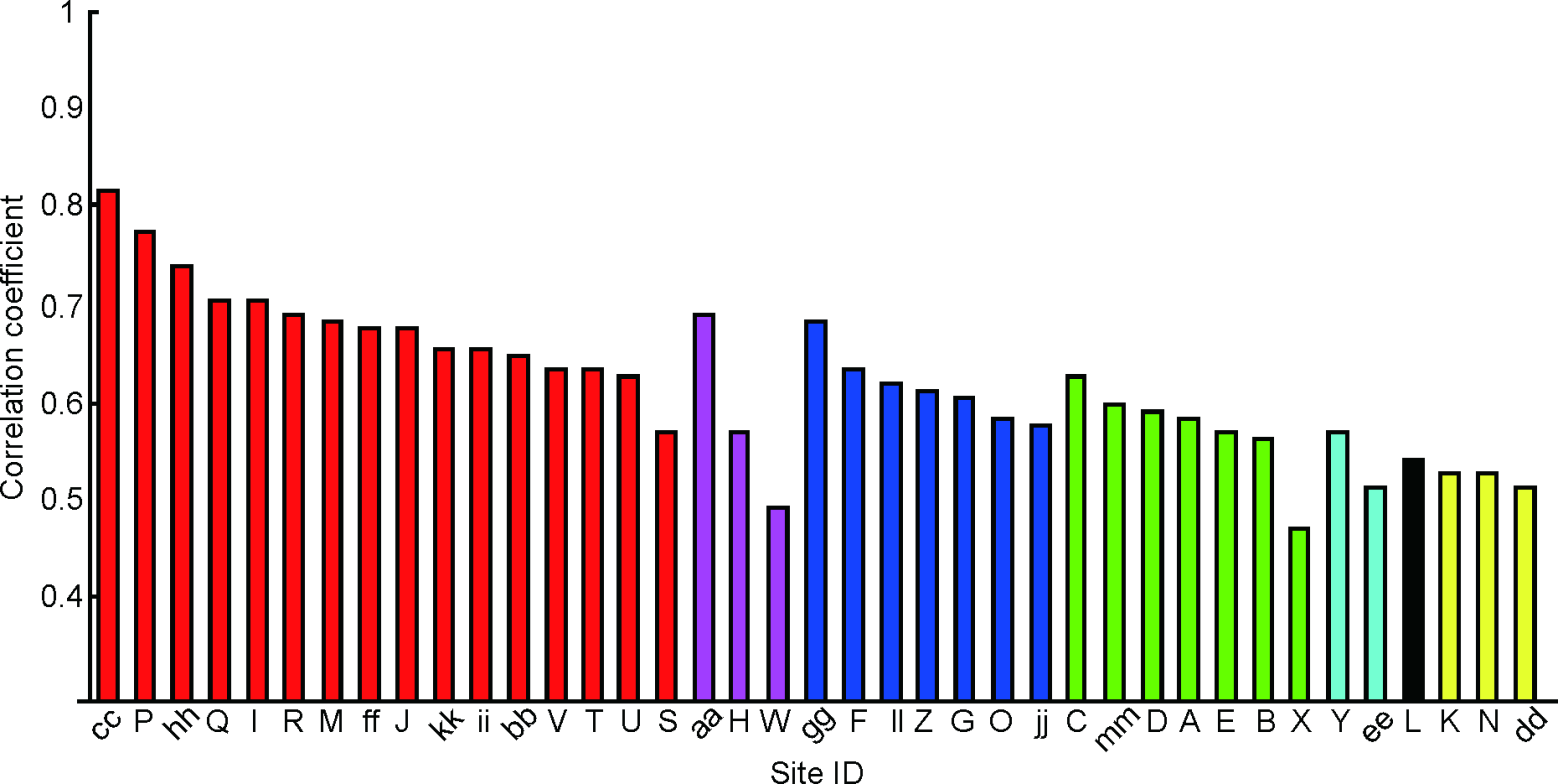
The mean c.c. value of the best features for all recording sites in H1 that include sites responding to non-face images. The color indicates different sub-regions of IT cortex defined by similarity in object responses, such as red for face domain, blue for monkey-body domain, and green for anti-face domain [11]. Particular object categories could not be identified for the other domains [11].

One obvious question is whether we can extend our fragment-based approach to neurons responding to non-face objects rather than faces. A preliminary analysis revealed that there was significant correlation between neural and feature-based response vectors as well as the sites in the face domains (Fig 14). However, the values of c.c. for the sites outside of the face domains were not as high as those in the face domain so that we may take additional factors into account. Further analyses of these sites remain for future investigations.

Deep convolutional neural networks (DCNNs) have recently attracted interest owing to their significantly high performance in invariant object categorization (for example, see [34]). DCNNs are hierarchical neural networks in which each stage comprises linear and nonlinear transforming layers that are similar to the simple and complex cells in V1. The hierarchical structure of DCNN is analogous to the primate ventral visual pathway and, in fact, the object responses of the highest object representation layer of a DCNN have been compared to IT neural responses by Yamins and colleagues, who found that the object responses of the DCNN layer explained 49% of the variance of IT neuron responses [35]. DCNNs may share common principles with the ventral visual pathway in terms of invariant object recognition. However, visual feature extraction from objects through the network hierarchy remains poorly characterized for both systems, and our method should be usable for the characterization of units in the DCNN object representation layer. This potential application, together with the ability of our method to extract features from IT neurons using large datasets, may serve as a stepping stone to addressing the common principles of invariant object recognition.

## Supporting information

S1 Fig **Representative images (A and B) of the standard set showing how feature candidates were cut out from the natural images.** A natural image (left) was preprocessed to represent the image in a local orientation and color space consisting of 4 orientations and 3 colors (right). As indicated by a grid, feature candidates were cut out from the preprocessed image throughout 4 orientations and 3 colors. Seventy feature candidates of 16 × 8 type were cut out in A, and thirty feature candidates of 4 × 16 type and thirty-five feature candidates of 12 × 16 were cut out in B. As indicated in red boxes, we showed natural image fragments in the main text that correspond to the feature candidates in the right.
(PDF)

S1 File 104 stimulus images for H1 and H2. (ZIP)

S2 File 1,000 stimulus images for H3. (ZIP)
