# Supplementary material for: Searching for visual features that explain response variance of face neurons in inferior temporal cortex
Source: PLoS One. 2018 Sep 20;13(9):e0201192. doi: 10.1371/journal.pone.0201192 (PMC6147465; doi:10.1371/journal.pone.0201192)

Representative natural images in a pixel space

Images transformed to a local orientation and color space

A

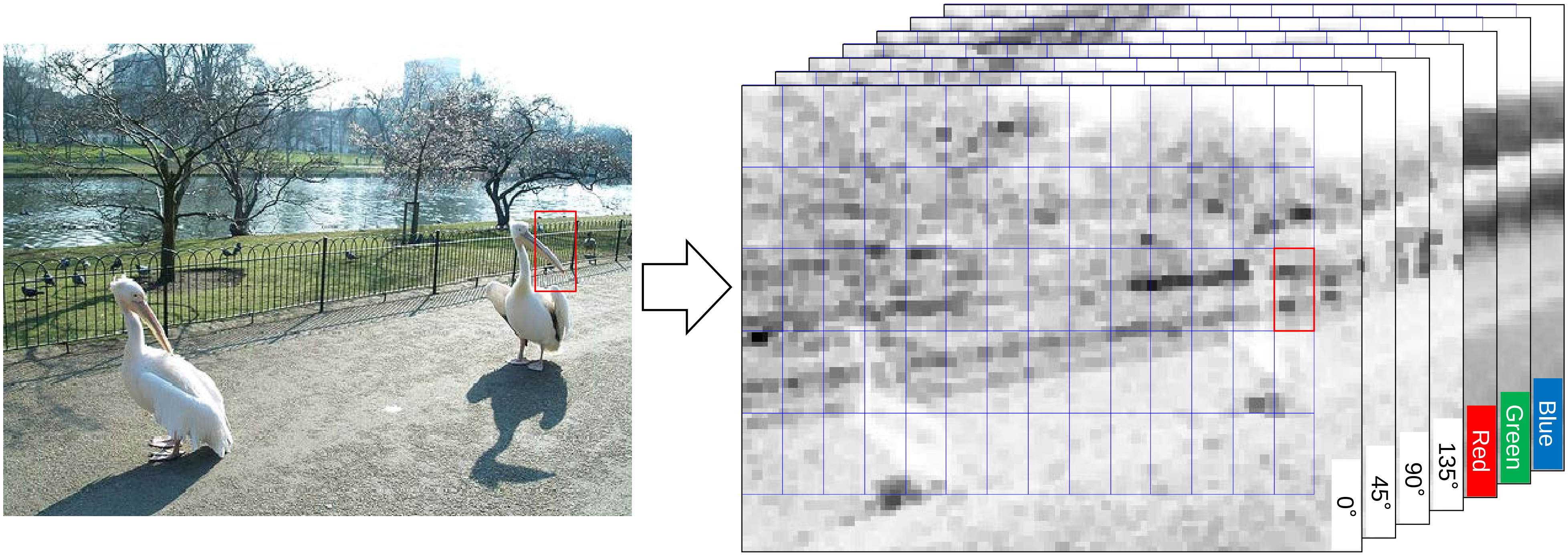

B

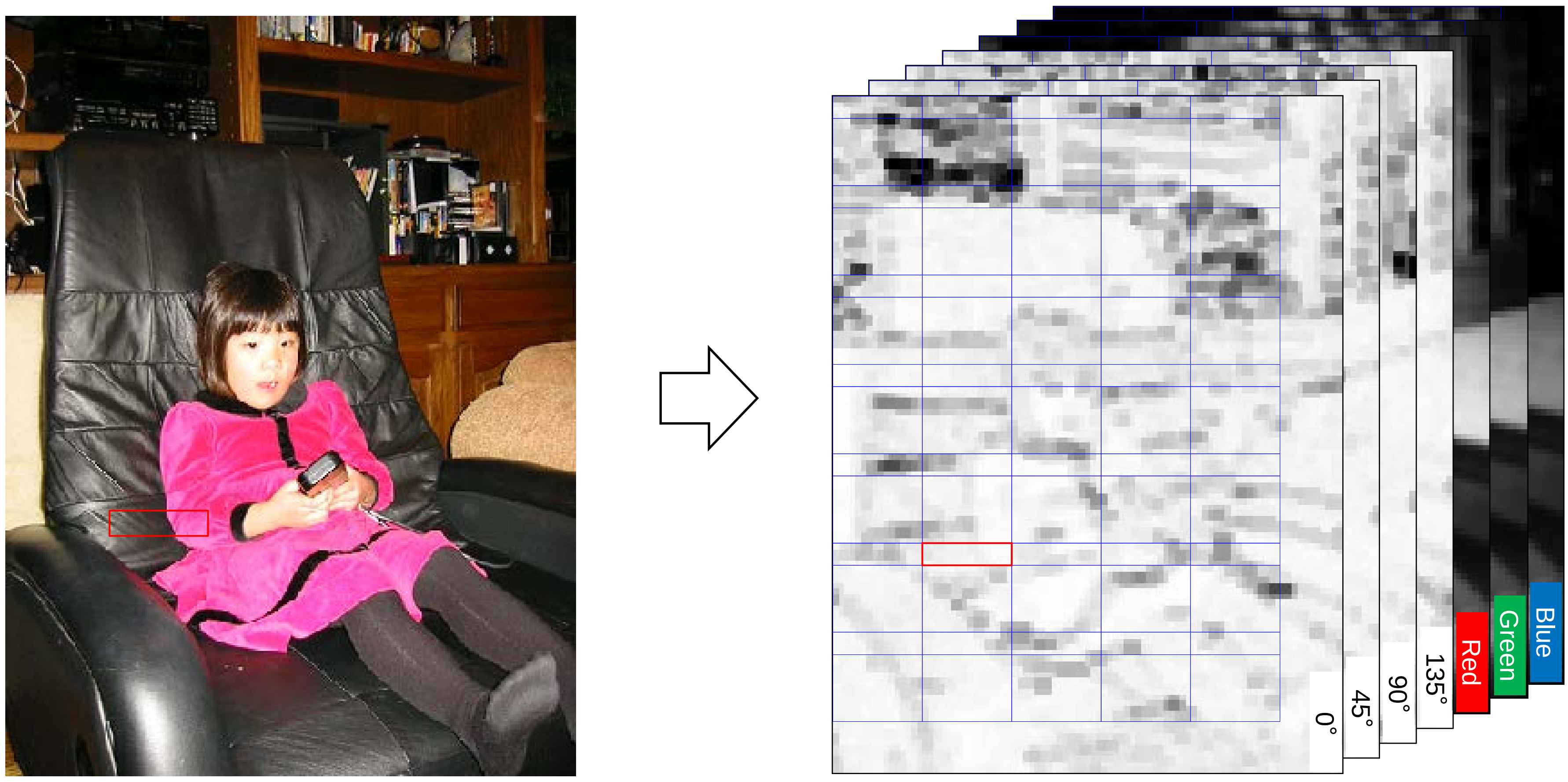

Supplement: S1 Fig — Representative images (A and B) of the standard set showing how feature candidates were cut out from the natural images. A natural image (left) was preprocessed to represent the image in a local orientation and color space consisting of 4 orientations and 3 colors (right). As indicated by a grid, feature candidates were cut out from the preprocessed image throughout 4 orientations and 3 colors. Seventy feature candidates of 16 × 8 type were cut out in A, and thirty feature candidates of 4 × 16 type and thirty-five feature candidates of 12 × 16 were cut out in B. As indicated in red boxes, we showed natural image fragments in the main text that correspond to the feature candidates in the right. (PDF) [file pone.0201192.s001.pdf]
