# Supplementary figures and images for: Searching for visual features that explain response variance of face neurons in inferior temporal cortex (part 2 of 4)
Source: PLoS One. 2018 Sep 20;13(9):e0201192. doi: 10.1371/journal.pone.0201192 (PMC6147465; doi:10.1371/journal.pone.0201192)

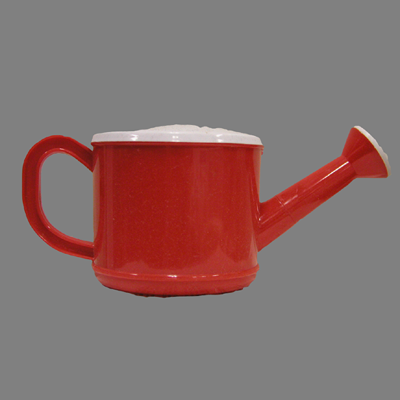

Supplement: S1 File — (ZIP) [file pone.0201192.s002.zip › S1/i13.bmp]

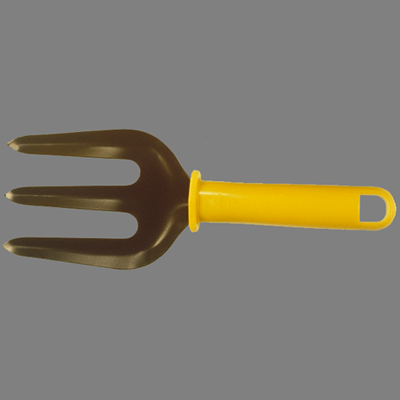

Supplement: S1 File — (ZIP) [file pone.0201192.s002.zip › S1/i14.bmp]

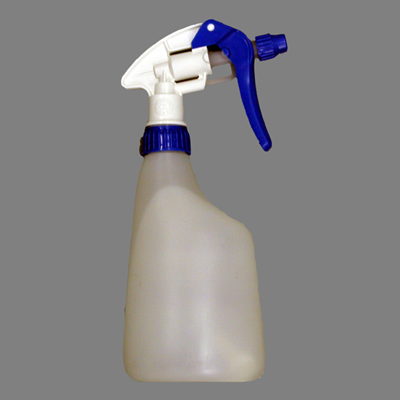

Supplement: S1 File — (ZIP) [file pone.0201192.s002.zip › S1/i15.bmp]

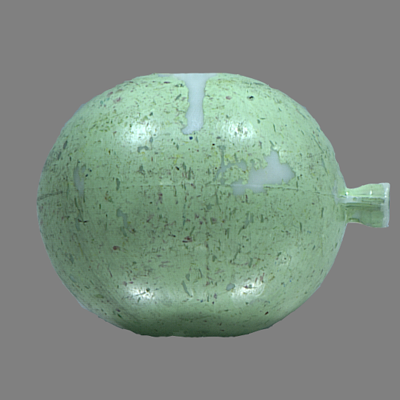

Supplement: S1 File — (ZIP) [file pone.0201192.s002.zip › S1/i16.bmp]

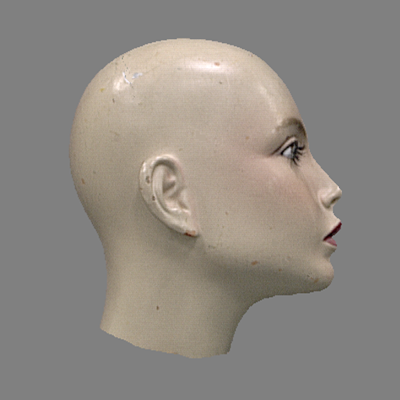

Supplement: S2 File — (ZIP) [file pone.0201192.s003.zip › S2/Fakeface1.png]

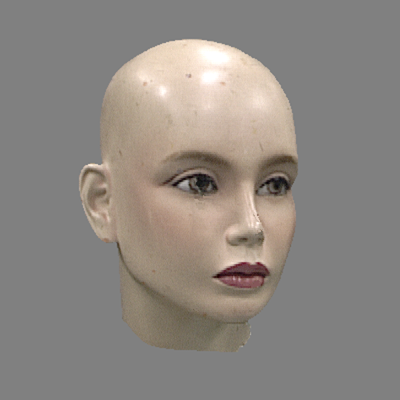

Supplement: S2 File — (ZIP) [file pone.0201192.s003.zip › S2/Fakeface2.png]

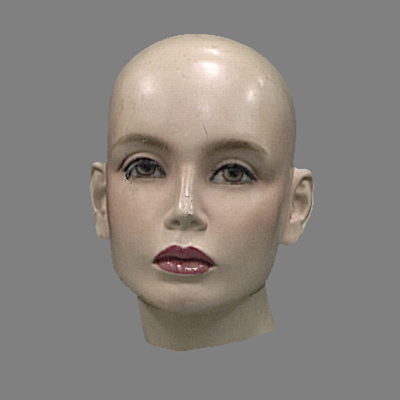

Supplement: S2 File — (ZIP) [file pone.0201192.s003.zip › S2/Fakeface3.png]

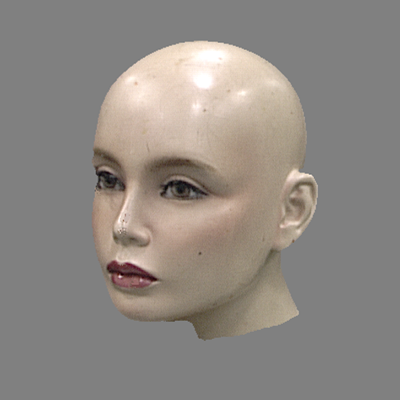

Supplement: S2 File — (ZIP) [file pone.0201192.s003.zip › S2/Fakeface4.png]

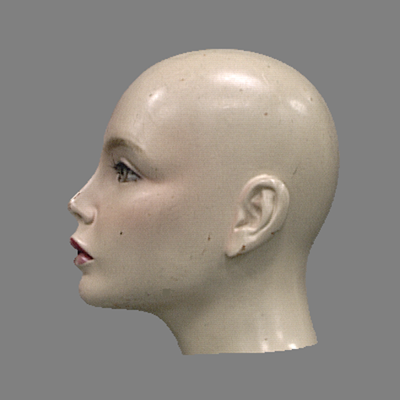

Supplement: S2 File — (ZIP) [file pone.0201192.s003.zip › S2/Fakeface5.png]

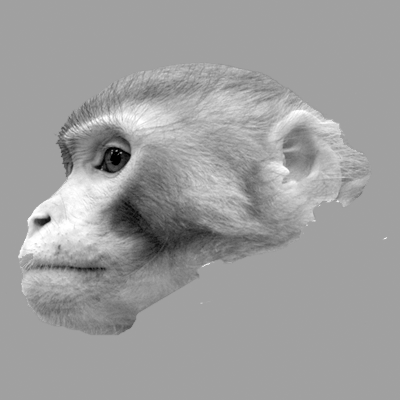

Supplement: S2 File — (ZIP) [file pone.0201192.s003.zip › S2/GrayMf022m1_1.png]

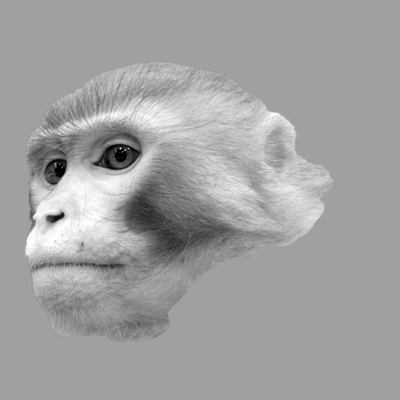

Supplement: S2 File — (ZIP) [file pone.0201192.s003.zip › S2/GrayMf022m1_2.png]

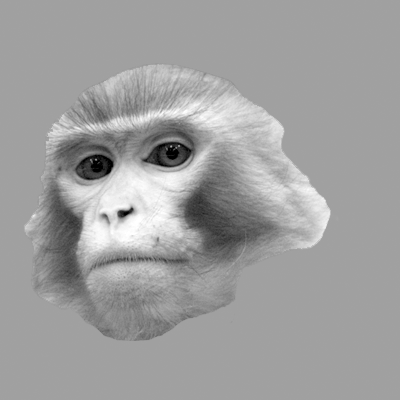

Supplement: S2 File — (ZIP) [file pone.0201192.s003.zip › S2/GrayMf022m1_3.png]

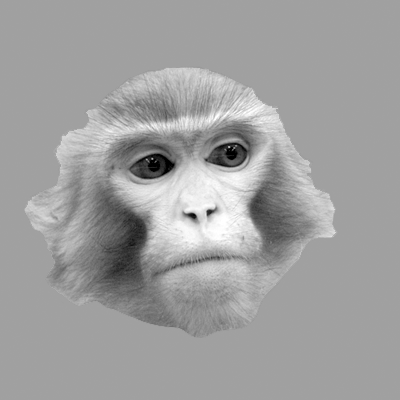

Supplement: S2 File — (ZIP) [file pone.0201192.s003.zip › S2/GrayMf022m1_4.png]

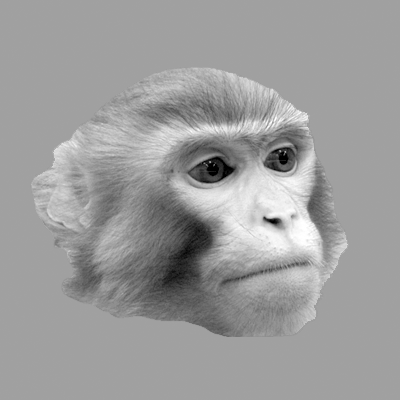

Supplement: S2 File — (ZIP) [file pone.0201192.s003.zip › S2/GrayMf022m1_5.png]

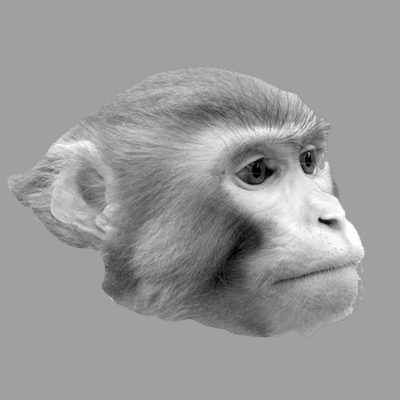

Supplement: S2 File — (ZIP) [file pone.0201192.s003.zip › S2/GrayMf022m1_6.png]

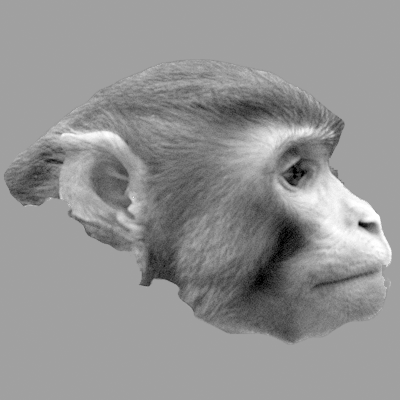

Supplement: S2 File — (ZIP) [file pone.0201192.s003.zip › S2/GrayMf022m1_7.png]

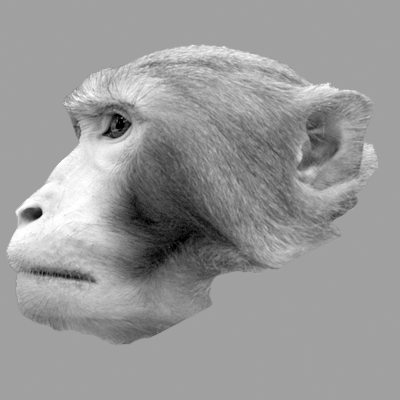

Supplement: S2 File — (ZIP) [file pone.0201192.s003.zip › S2/GrayMf023m2_1.png]

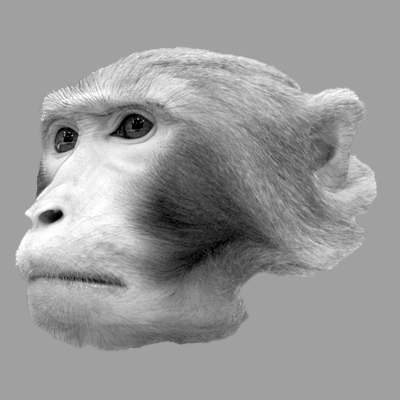

Supplement: S2 File — (ZIP) [file pone.0201192.s003.zip › S2/GrayMf023m2_2.png]

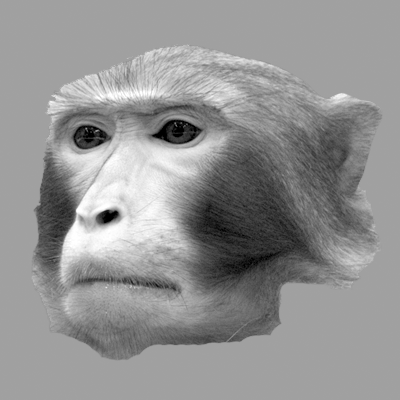

Supplement: S2 File — (ZIP) [file pone.0201192.s003.zip › S2/GrayMf023m2_3.png]

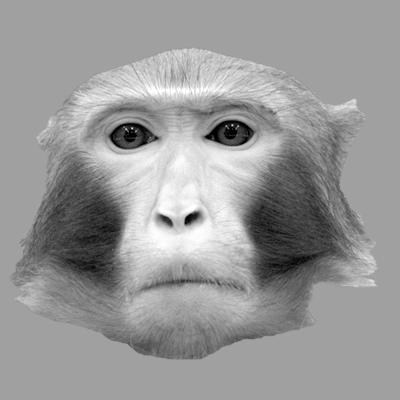

Supplement: S2 File — (ZIP) [file pone.0201192.s003.zip › S2/GrayMf023m2_4.png]

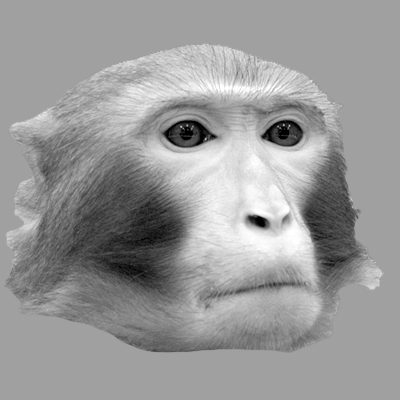

Supplement: S2 File — (ZIP) [file pone.0201192.s003.zip › S2/GrayMf023m2_5.png]

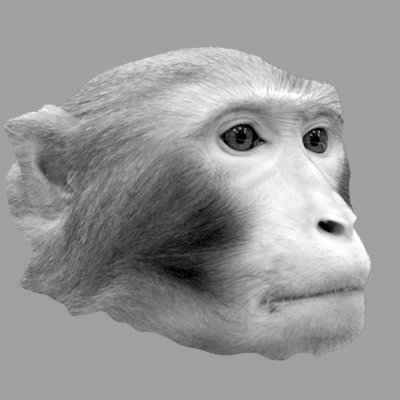

Supplement: S2 File — (ZIP) [file pone.0201192.s003.zip › S2/GrayMf023m2_6.png]

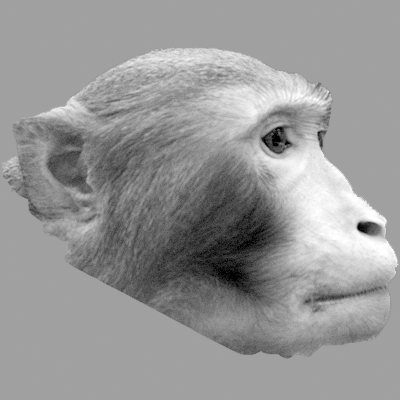

Supplement: S2 File — (ZIP) [file pone.0201192.s003.zip › S2/GrayMf023m2_7.png]

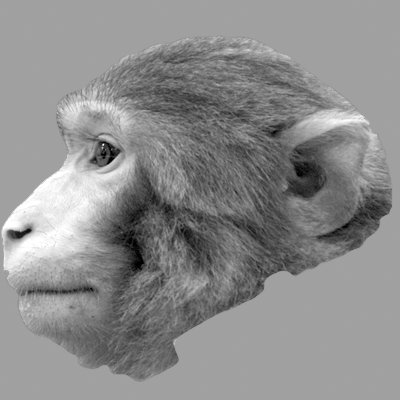

Supplement: S2 File — (ZIP) [file pone.0201192.s003.zip › S2/GrayMf024m3_1.png]

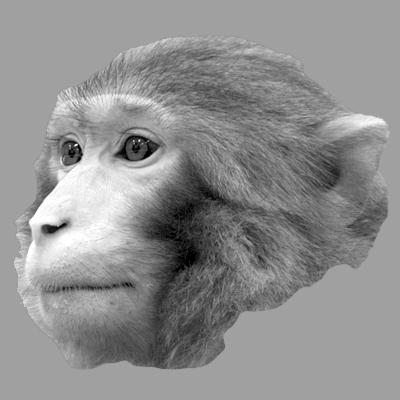

Supplement: S2 File — (ZIP) [file pone.0201192.s003.zip › S2/GrayMf024m3_2.png]

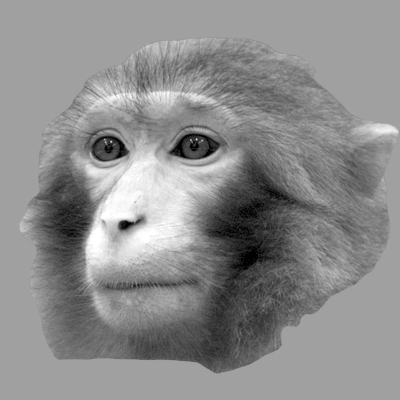

Supplement: S2 File — (ZIP) [file pone.0201192.s003.zip › S2/GrayMf024m3_3.png]

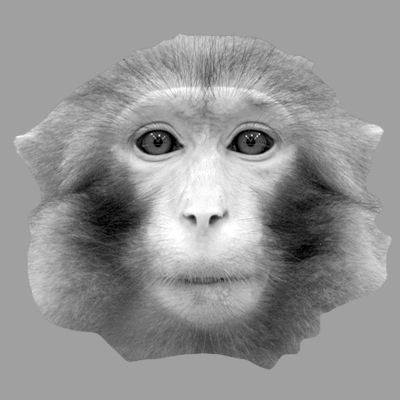

Supplement: S2 File — (ZIP) [file pone.0201192.s003.zip › S2/GrayMf024m3_4.png]

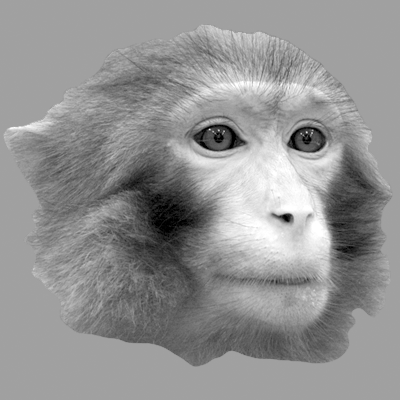

Supplement: S2 File — (ZIP) [file pone.0201192.s003.zip › S2/GrayMf024m3_5.png]

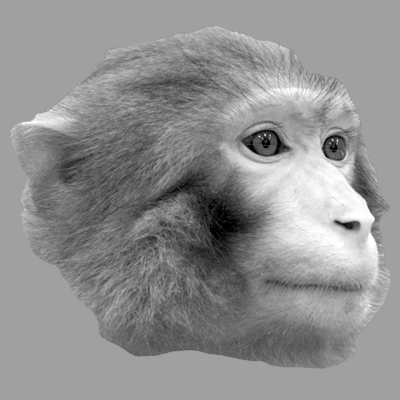

Supplement: S2 File — (ZIP) [file pone.0201192.s003.zip › S2/GrayMf024m3_6.png]

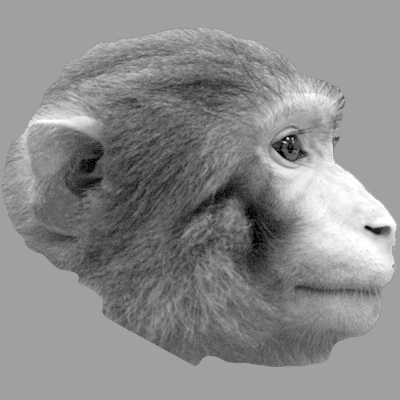

Supplement: S2 File — (ZIP) [file pone.0201192.s003.zip › S2/GrayMf024m3_7.png]

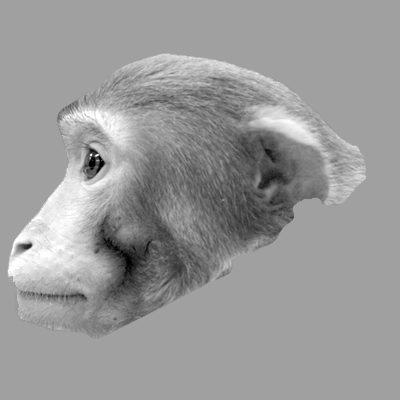

Supplement: S2 File — (ZIP) [file pone.0201192.s003.zip › S2/GrayMf025m4_1.png]

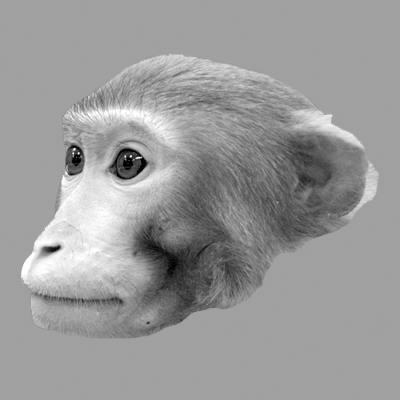

Supplement: S2 File — (ZIP) [file pone.0201192.s003.zip › S2/GrayMf025m4_2.png]

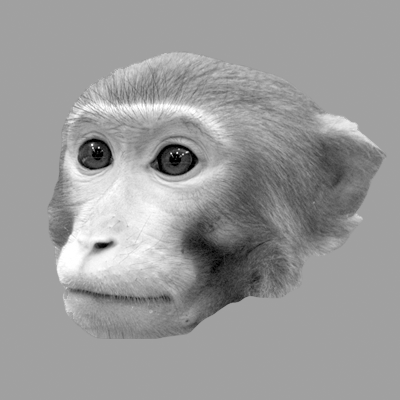

Supplement: S2 File — (ZIP) [file pone.0201192.s003.zip › S2/GrayMf025m4_3.png]

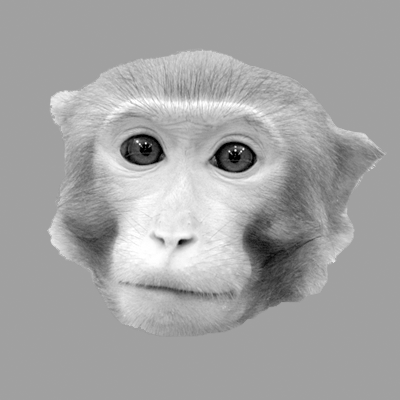

Supplement: S2 File — (ZIP) [file pone.0201192.s003.zip › S2/GrayMf025m4_4.png]

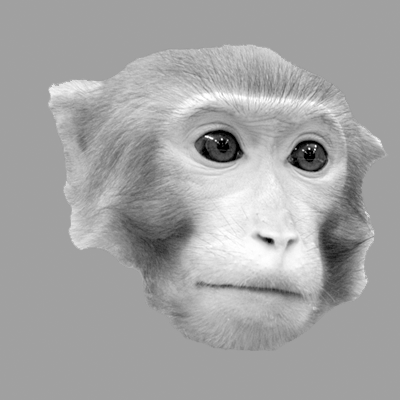

Supplement: S2 File — (ZIP) [file pone.0201192.s003.zip › S2/GrayMf025m4_5.png]

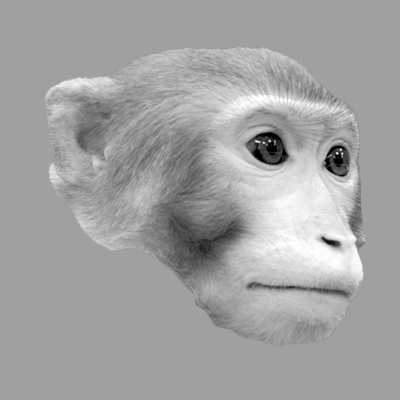

Supplement: S2 File — (ZIP) [file pone.0201192.s003.zip › S2/GrayMf025m4_6.png]

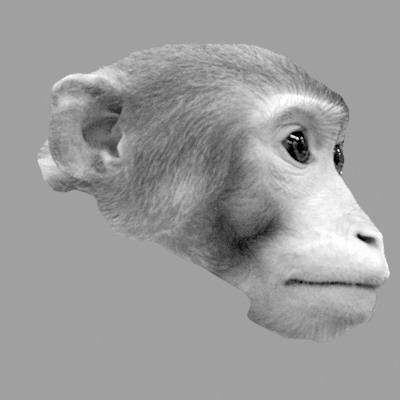

Supplement: S2 File — (ZIP) [file pone.0201192.s003.zip › S2/GrayMf025m4_7.png]

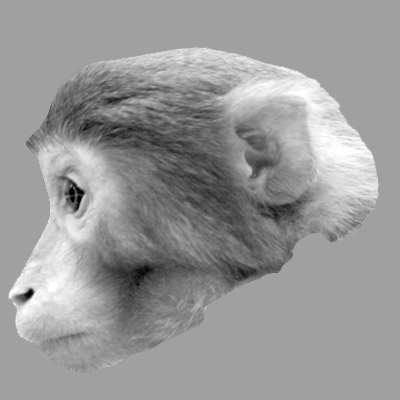

Supplement: S2 File — (ZIP) [file pone.0201192.s003.zip › S2/GrayMf026m5_1.png]

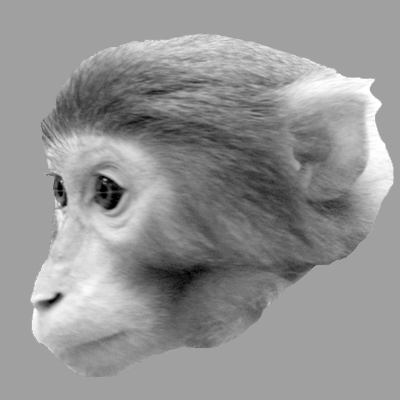

Supplement: S2 File — (ZIP) [file pone.0201192.s003.zip › S2/GrayMf026m5_2.png]

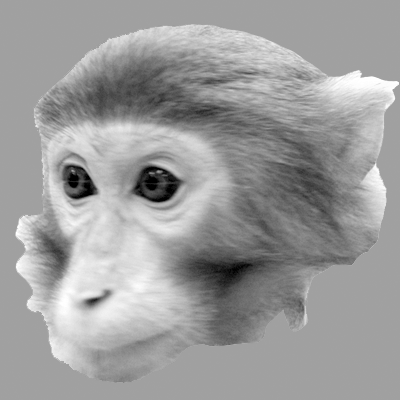

Supplement: S2 File — (ZIP) [file pone.0201192.s003.zip › S2/GrayMf026m5_3.png]

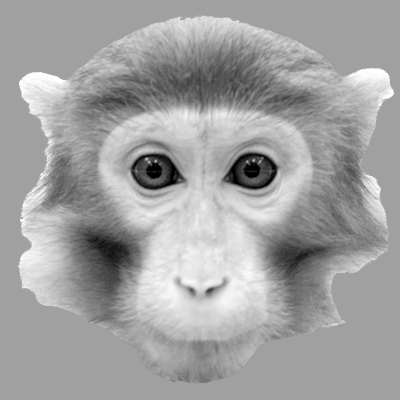

Supplement: S2 File — (ZIP) [file pone.0201192.s003.zip › S2/GrayMf026m5_4.png]

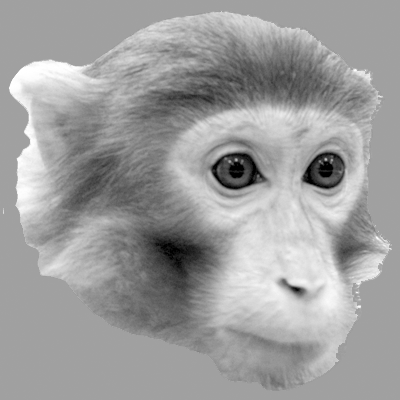

Supplement: S2 File — (ZIP) [file pone.0201192.s003.zip › S2/GrayMf026m5_5.png]

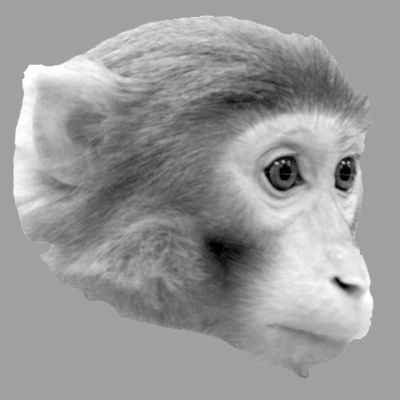

Supplement: S2 File — (ZIP) [file pone.0201192.s003.zip › S2/GrayMf026m5_6.png]

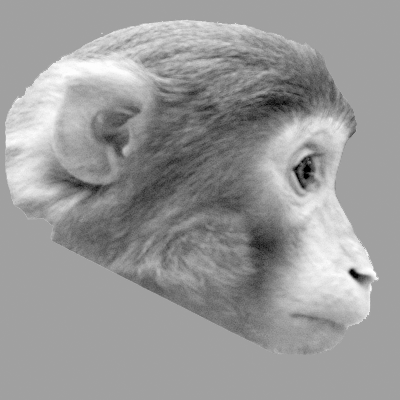

Supplement: S2 File — (ZIP) [file pone.0201192.s003.zip › S2/GrayMf026m5_7.png]

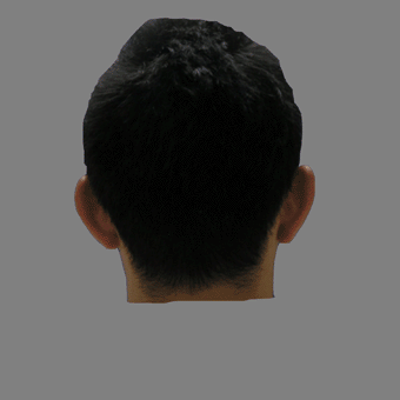

Supplement: S2 File — (ZIP) [file pone.0201192.s003.zip › S2/Hf001.png]

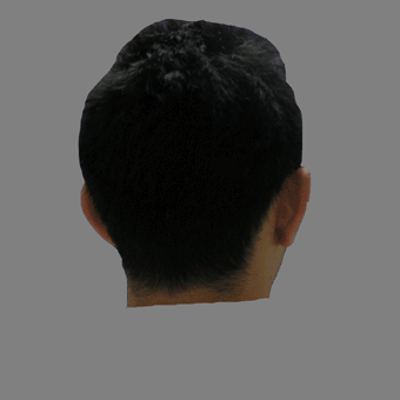

Supplement: S2 File — (ZIP) [file pone.0201192.s003.zip › S2/Hf003.png]

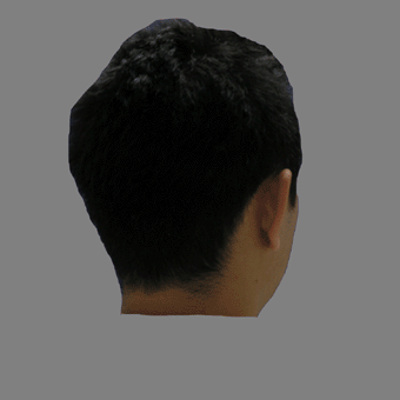

Supplement: S2 File — (ZIP) [file pone.0201192.s003.zip › S2/Hf005.png]

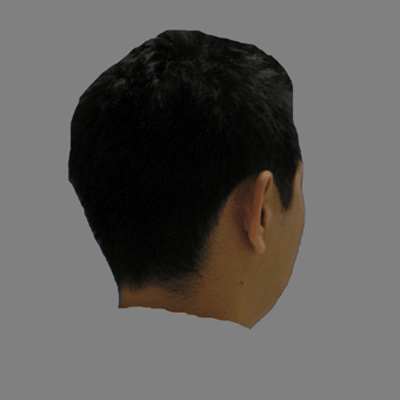

Supplement: S2 File — (ZIP) [file pone.0201192.s003.zip › S2/Hf007.png]

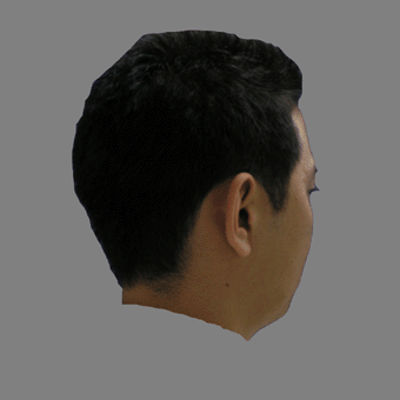

Supplement: S2 File — (ZIP) [file pone.0201192.s003.zip › S2/Hf009.png]

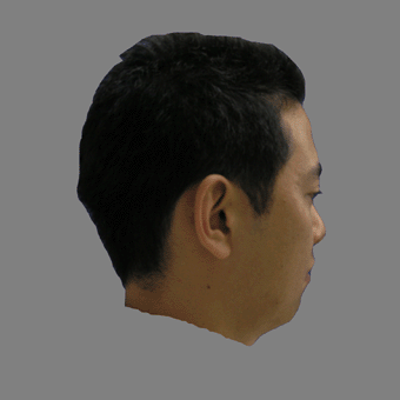

Supplement: S2 File — (ZIP) [file pone.0201192.s003.zip › S2/Hf011.png]

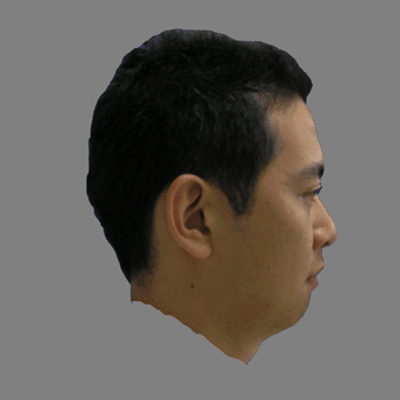

Supplement: S2 File — (ZIP) [file pone.0201192.s003.zip › S2/Hf013.png]

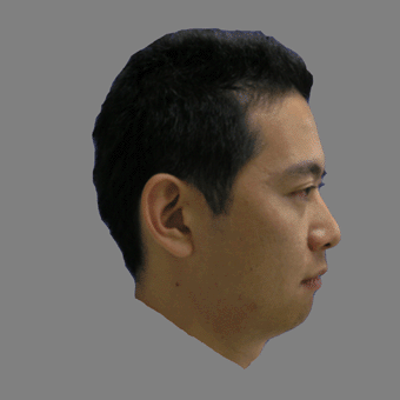

Supplement: S2 File — (ZIP) [file pone.0201192.s003.zip › S2/Hf015.png]

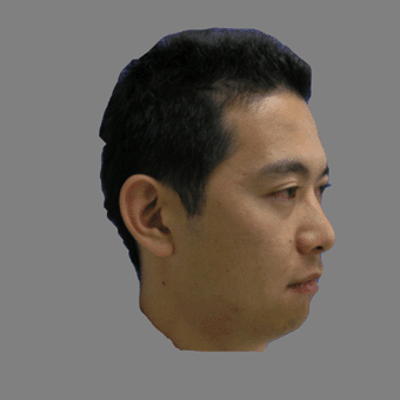

Supplement: S2 File — (ZIP) [file pone.0201192.s003.zip › S2/Hf017.png]

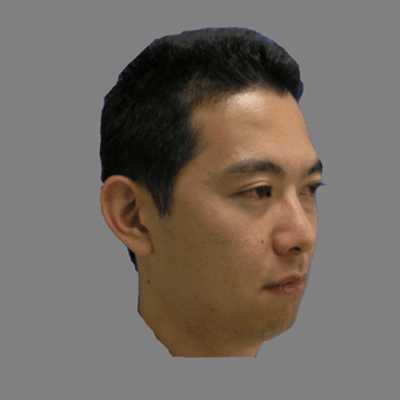

Supplement: S2 File — (ZIP) [file pone.0201192.s003.zip › S2/Hf019.png]

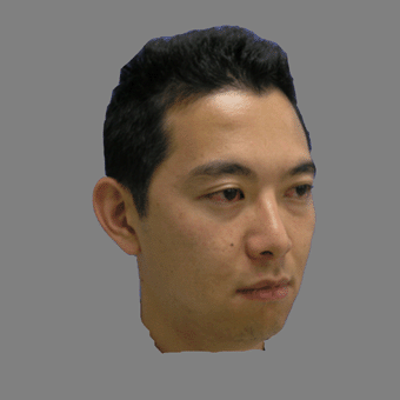

Supplement: S2 File — (ZIP) [file pone.0201192.s003.zip › S2/Hf021.png]

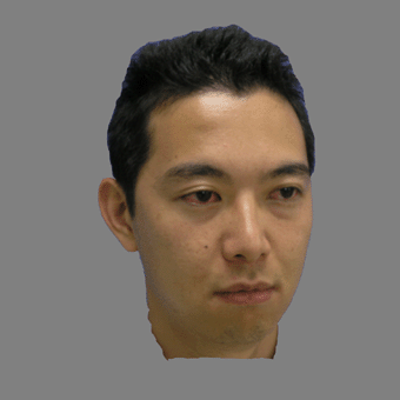

Supplement: S2 File — (ZIP) [file pone.0201192.s003.zip › S2/Hf023.png]

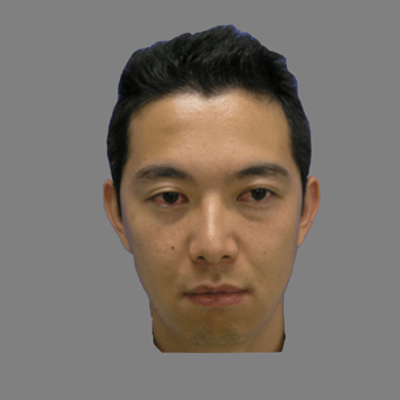

Supplement: S2 File — (ZIP) [file pone.0201192.s003.zip › S2/Hf025.png]

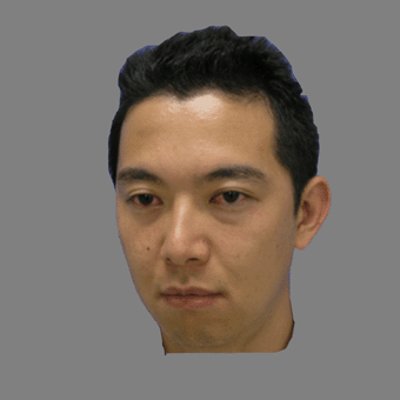

Supplement: S2 File — (ZIP) [file pone.0201192.s003.zip › S2/Hf027.png]

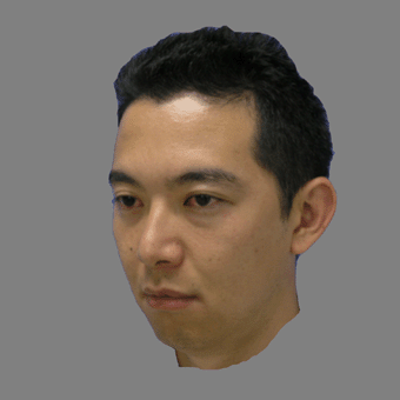

Supplement: S2 File — (ZIP) [file pone.0201192.s003.zip › S2/Hf029.png]

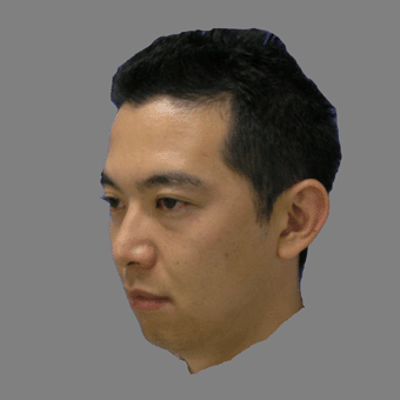

Supplement: S2 File — (ZIP) [file pone.0201192.s003.zip › S2/Hf031.png]

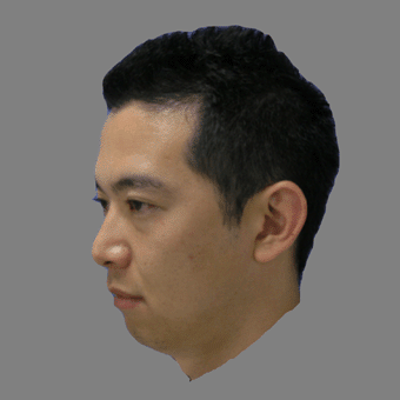

Supplement: S2 File — (ZIP) [file pone.0201192.s003.zip › S2/Hf033.png]

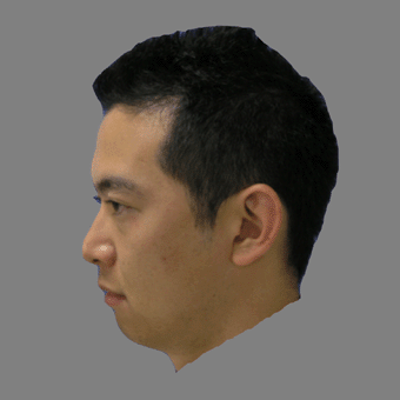

Supplement: S2 File — (ZIP) [file pone.0201192.s003.zip › S2/Hf035.png]

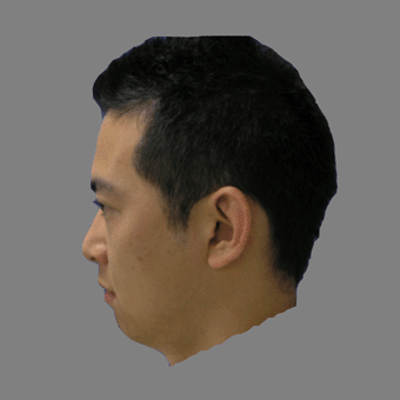

Supplement: S2 File — (ZIP) [file pone.0201192.s003.zip › S2/Hf037.png]

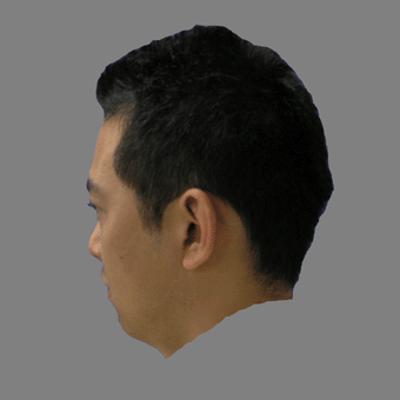

Supplement: S2 File — (ZIP) [file pone.0201192.s003.zip › S2/Hf039.png]

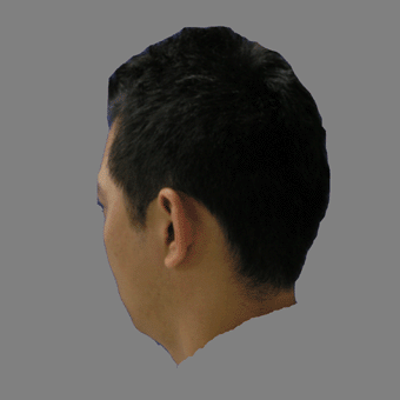

Supplement: S2 File — (ZIP) [file pone.0201192.s003.zip › S2/Hf041.png]

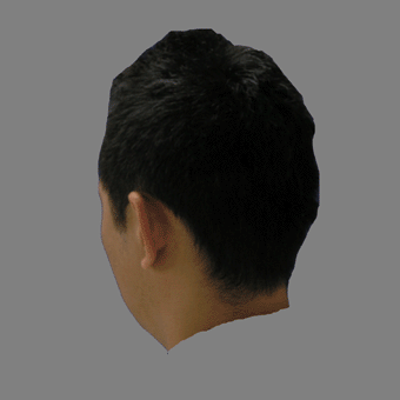

Supplement: S2 File — (ZIP) [file pone.0201192.s003.zip › S2/Hf043.png]

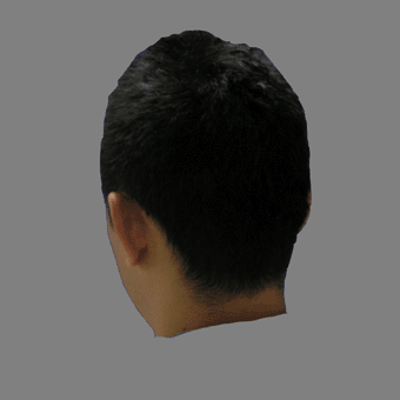

Supplement: S2 File — (ZIP) [file pone.0201192.s003.zip › S2/Hf045.png]

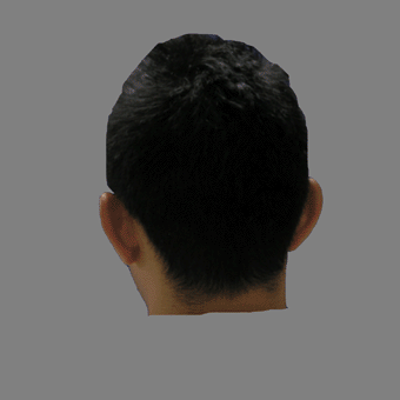

Supplement: S2 File — (ZIP) [file pone.0201192.s003.zip › S2/Hf047.png]

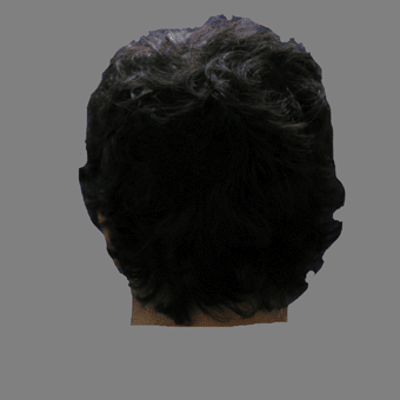

Supplement: S2 File — (ZIP) [file pone.0201192.s003.zip › S2/Hf049.png]

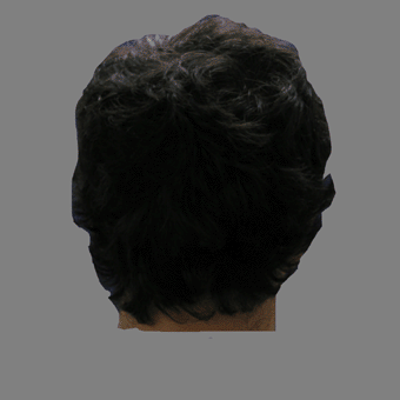

Supplement: S2 File — (ZIP) [file pone.0201192.s003.zip › S2/Hf051.png]

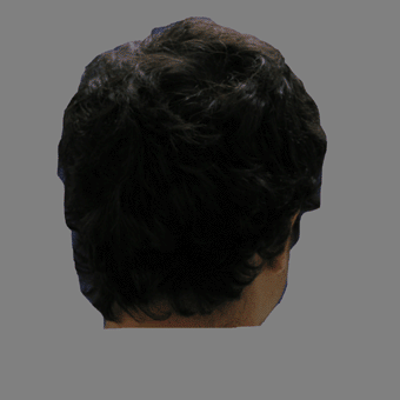

Supplement: S2 File — (ZIP) [file pone.0201192.s003.zip › S2/Hf053.png]

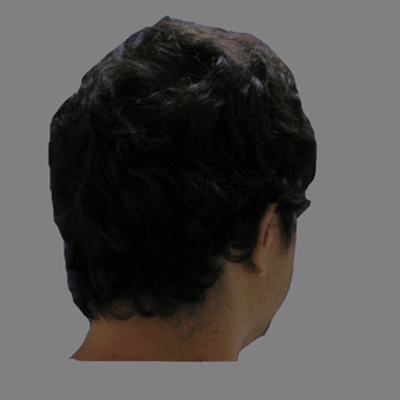

Supplement: S2 File — (ZIP) [file pone.0201192.s003.zip › S2/Hf055.png]

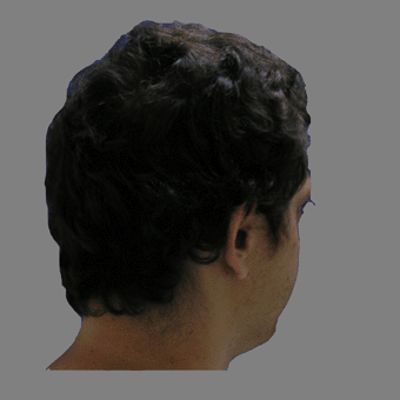

Supplement: S2 File — (ZIP) [file pone.0201192.s003.zip › S2/Hf057.png]

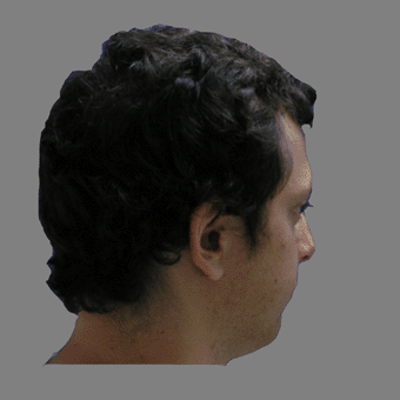

Supplement: S2 File — (ZIP) [file pone.0201192.s003.zip › S2/Hf059.png]

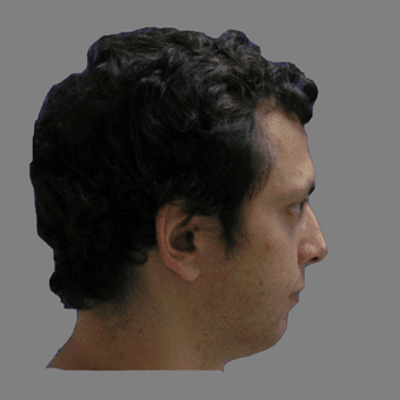

Supplement: S2 File — (ZIP) [file pone.0201192.s003.zip › S2/Hf061.png]

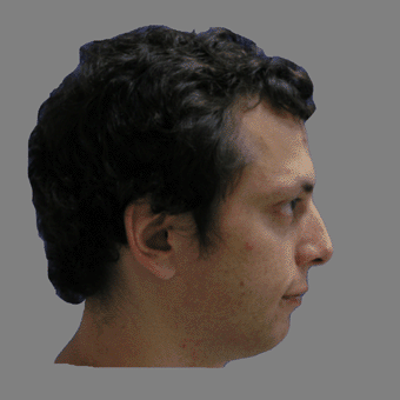

Supplement: S2 File — (ZIP) [file pone.0201192.s003.zip › S2/Hf063.png]

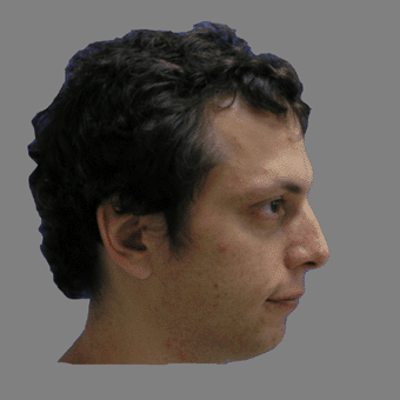

Supplement: S2 File — (ZIP) [file pone.0201192.s003.zip › S2/Hf065.png]

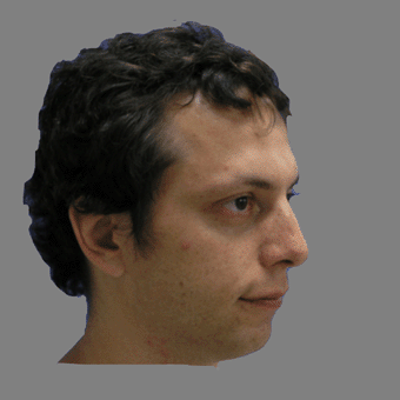

Supplement: S2 File — (ZIP) [file pone.0201192.s003.zip › S2/Hf067.png]

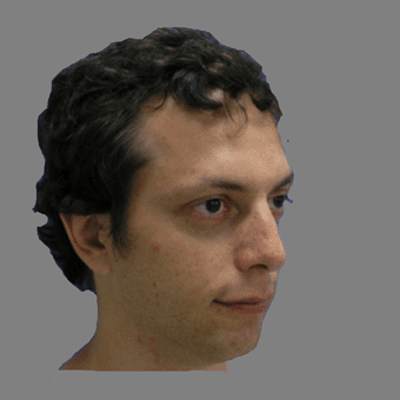

Supplement: S2 File — (ZIP) [file pone.0201192.s003.zip › S2/Hf069.png]

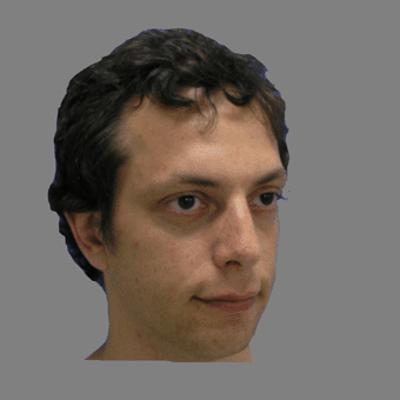

Supplement: S2 File — (ZIP) [file pone.0201192.s003.zip › S2/Hf071.png]

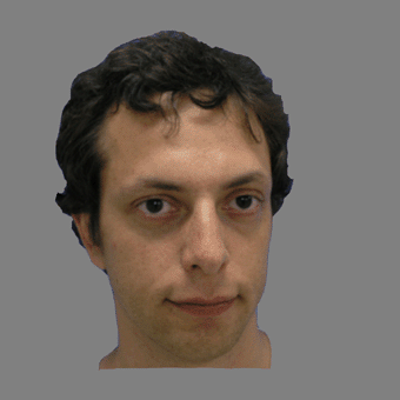

Supplement: S2 File — (ZIP) [file pone.0201192.s003.zip › S2/Hf073.png]

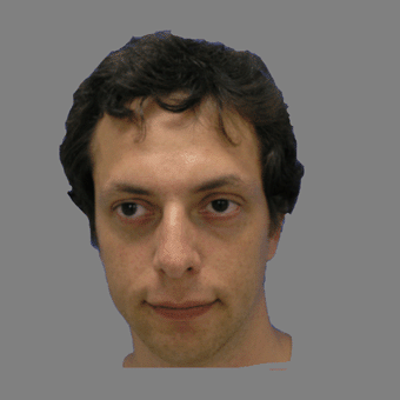

Supplement: S2 File — (ZIP) [file pone.0201192.s003.zip › S2/Hf075.png]

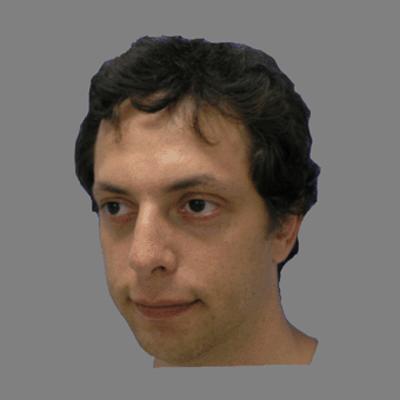

Supplement: S2 File — (ZIP) [file pone.0201192.s003.zip › S2/Hf077.png]

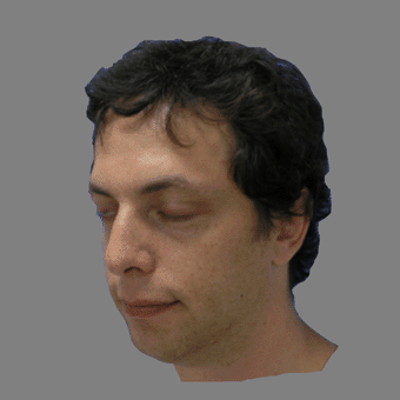

Supplement: S2 File — (ZIP) [file pone.0201192.s003.zip › S2/Hf079.png]

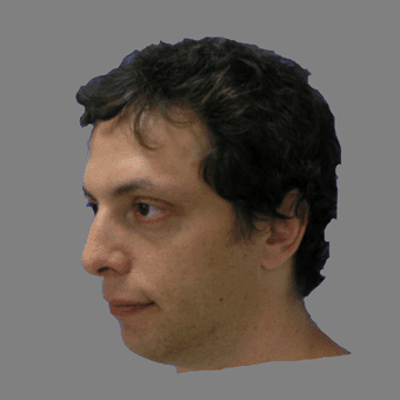

Supplement: S2 File — (ZIP) [file pone.0201192.s003.zip › S2/Hf081.png]

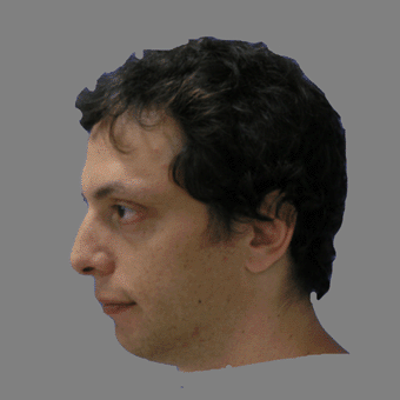

Supplement: S2 File — (ZIP) [file pone.0201192.s003.zip › S2/Hf083.png]

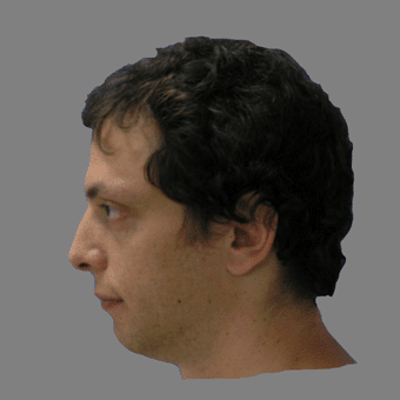

Supplement: S2 File — (ZIP) [file pone.0201192.s003.zip › S2/Hf085.png]

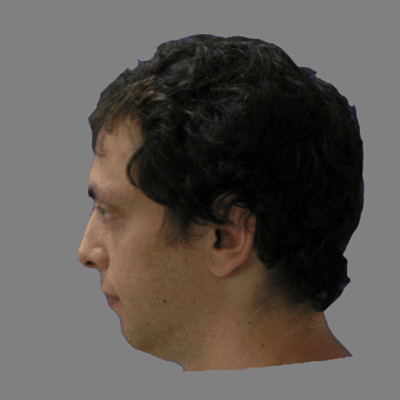

Supplement: S2 File — (ZIP) [file pone.0201192.s003.zip › S2/Hf087.png]

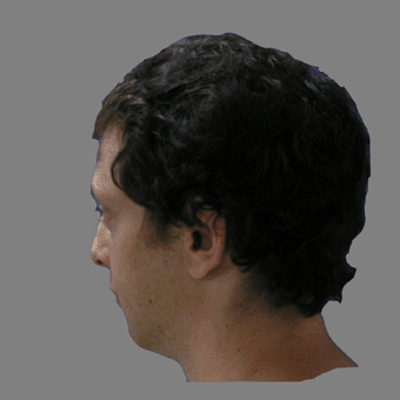

Supplement: S2 File — (ZIP) [file pone.0201192.s003.zip › S2/Hf089.png]

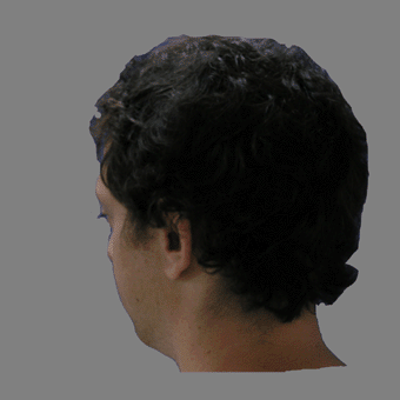

Supplement: S2 File — (ZIP) [file pone.0201192.s003.zip › S2/Hf091.png]

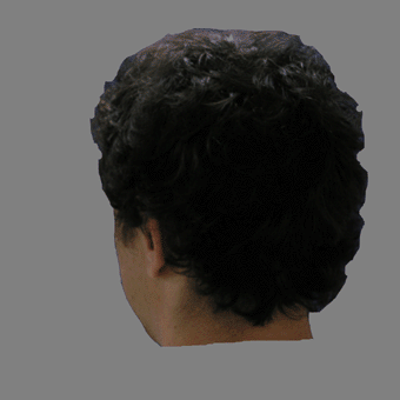

Supplement: S2 File — (ZIP) [file pone.0201192.s003.zip › S2/Hf093.png]

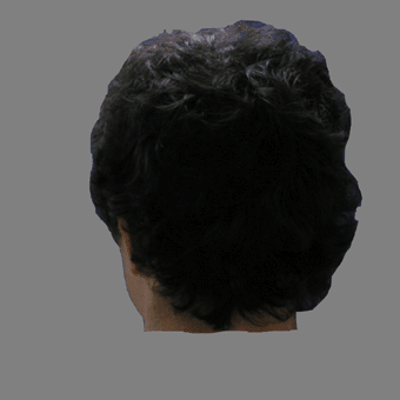

Supplement: S2 File — (ZIP) [file pone.0201192.s003.zip › S2/Hf095.png]

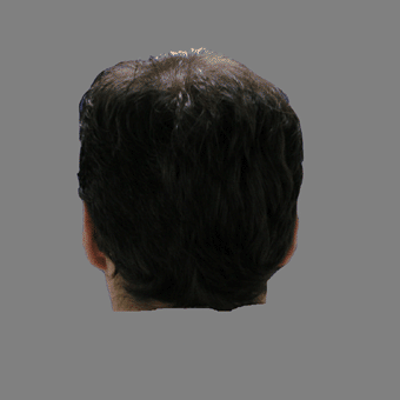

Supplement: S2 File — (ZIP) [file pone.0201192.s003.zip › S2/Hf097.png]

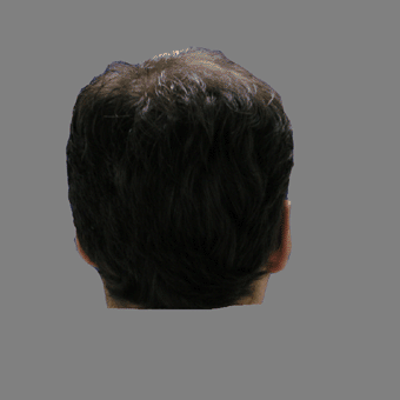

Supplement: S2 File — (ZIP) [file pone.0201192.s003.zip › S2/Hf099.png]

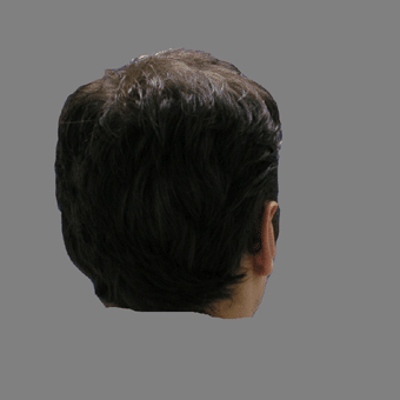

Supplement: S2 File — (ZIP) [file pone.0201192.s003.zip › S2/Hf101.png]

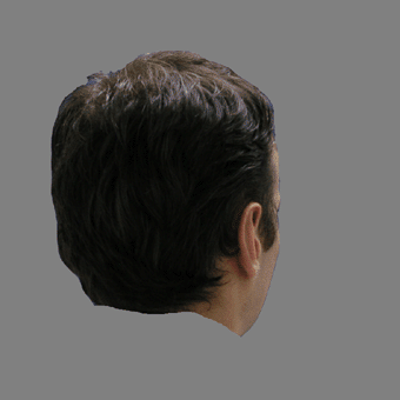

Supplement: S2 File — (ZIP) [file pone.0201192.s003.zip › S2/Hf103.png]

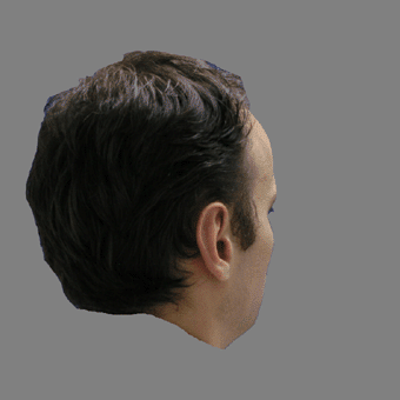

Supplement: S2 File — (ZIP) [file pone.0201192.s003.zip › S2/Hf105.png]

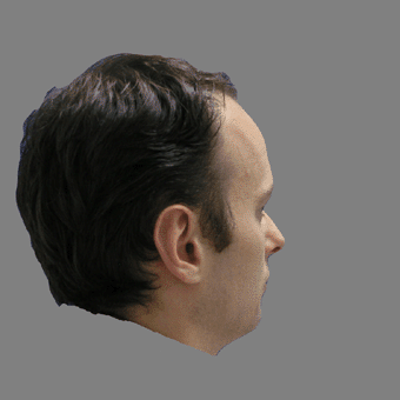

Supplement: S2 File — (ZIP) [file pone.0201192.s003.zip › S2/Hf107.png]

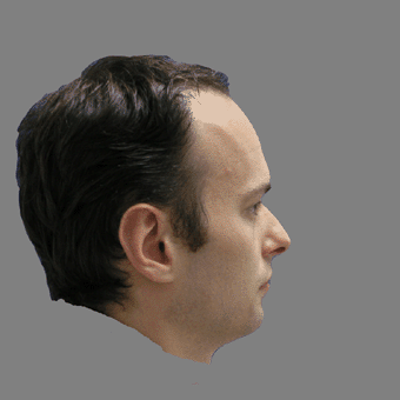

Supplement: S2 File — (ZIP) [file pone.0201192.s003.zip › S2/Hf109.png]

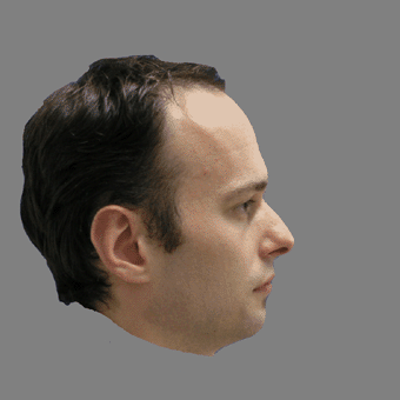

Supplement: S2 File — (ZIP) [file pone.0201192.s003.zip › S2/Hf111.png]
