# Supplementary figures and images for: Searching for visual features that explain response variance of face neurons in inferior temporal cortex (part 1 of 4)
Source: PLoS One. 2018 Sep 20;13(9):e0201192. doi: 10.1371/journal.pone.0201192 (PMC6147465; doi:10.1371/journal.pone.0201192)

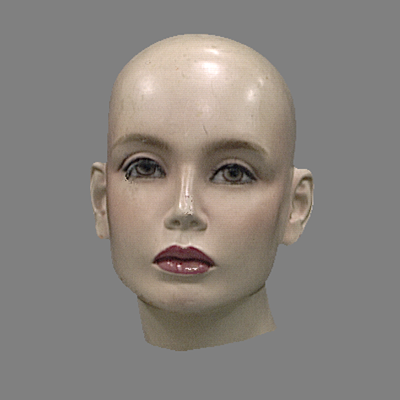

Supplement: S1 File — (ZIP) [file pone.0201192.s002.zip › S1/a01.bmp]

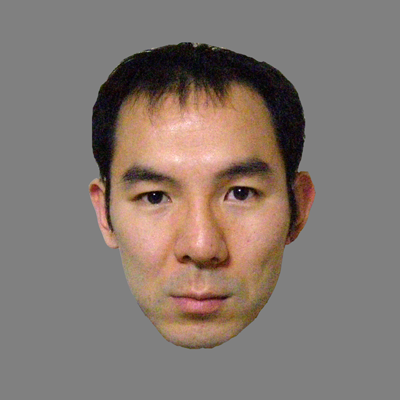

Supplement: S1 File — (ZIP) [file pone.0201192.s002.zip › S1/a02.bmp]

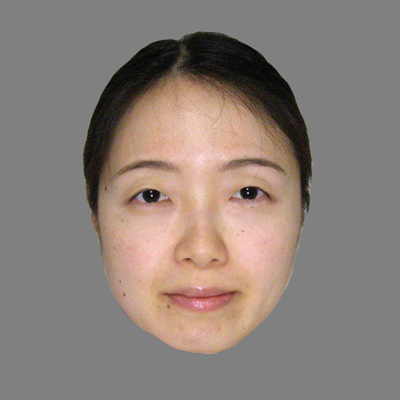

Supplement: S1 File — (ZIP) [file pone.0201192.s002.zip › S1/a03.bmp]

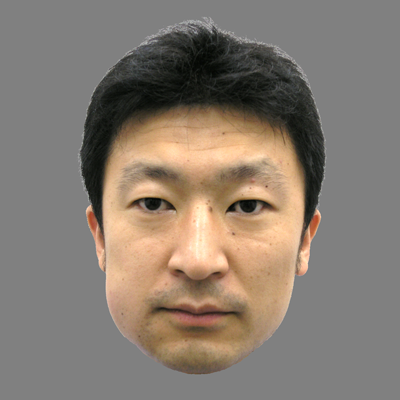

Supplement: S1 File — (ZIP) [file pone.0201192.s002.zip › S1/a04.bmp]

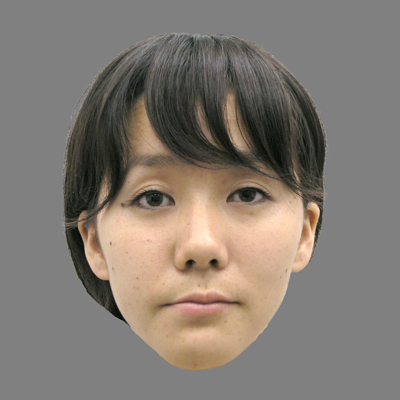

Supplement: S1 File — (ZIP) [file pone.0201192.s002.zip › S1/a05.bmp]

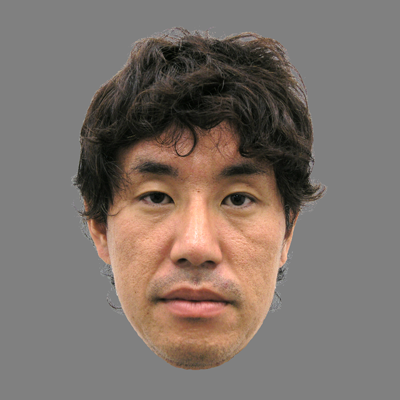

Supplement: S1 File — (ZIP) [file pone.0201192.s002.zip › S1/a06.bmp]

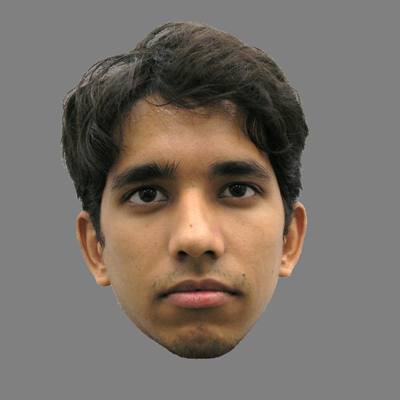

Supplement: S1 File — (ZIP) [file pone.0201192.s002.zip › S1/a07.bmp]

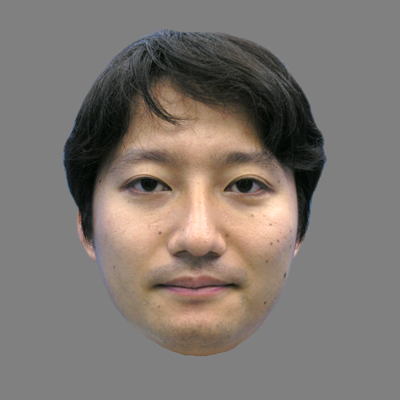

Supplement: S1 File — (ZIP) [file pone.0201192.s002.zip › S1/a08.bmp]

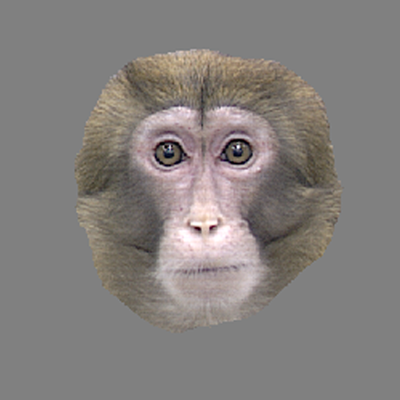

Supplement: S1 File — (ZIP) [file pone.0201192.s002.zip › S1/b01.bmp]

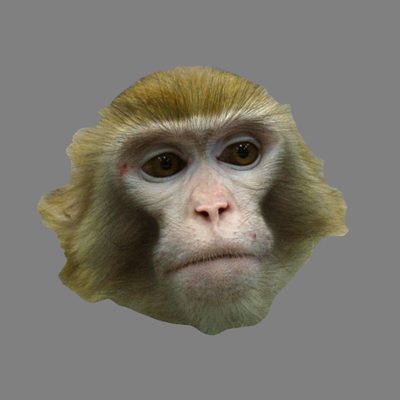

Supplement: S1 File — (ZIP) [file pone.0201192.s002.zip › S1/b02.bmp]

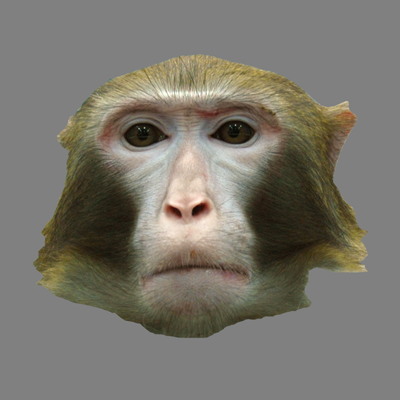

Supplement: S1 File — (ZIP) [file pone.0201192.s002.zip › S1/b03.bmp]

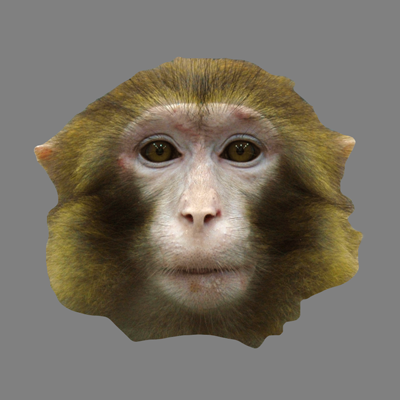

Supplement: S1 File — (ZIP) [file pone.0201192.s002.zip › S1/b04.bmp]

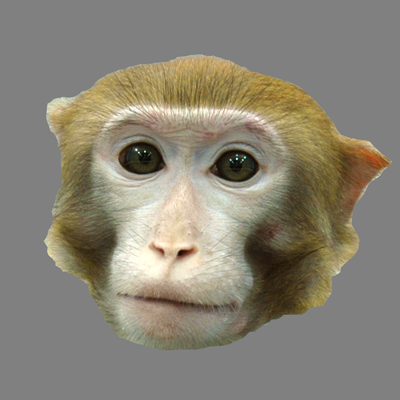

Supplement: S1 File — (ZIP) [file pone.0201192.s002.zip › S1/b05.bmp]

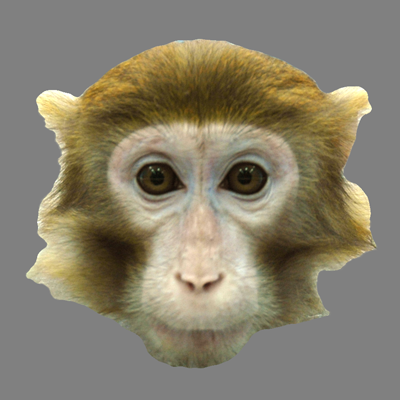

Supplement: S1 File — (ZIP) [file pone.0201192.s002.zip › S1/b06.bmp]

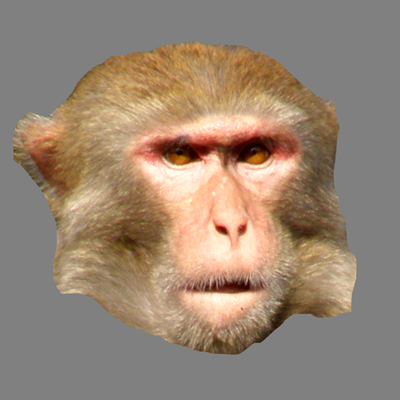

Supplement: S1 File — (ZIP) [file pone.0201192.s002.zip › S1/b07.bmp]

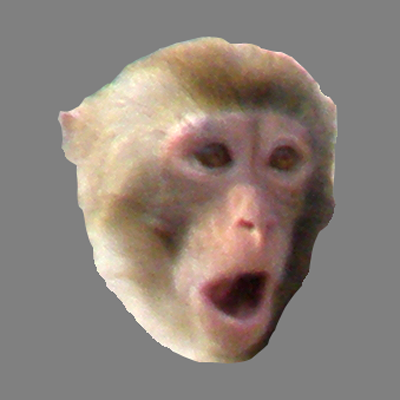

Supplement: S1 File — (ZIP) [file pone.0201192.s002.zip › S1/b08.bmp]

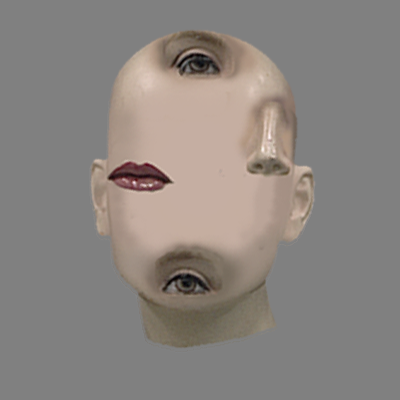

Supplement: S1 File — (ZIP) [file pone.0201192.s002.zip › S1/c01.bmp]

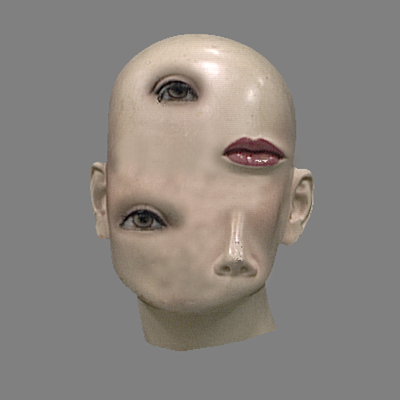

Supplement: S1 File — (ZIP) [file pone.0201192.s002.zip › S1/c02.bmp]

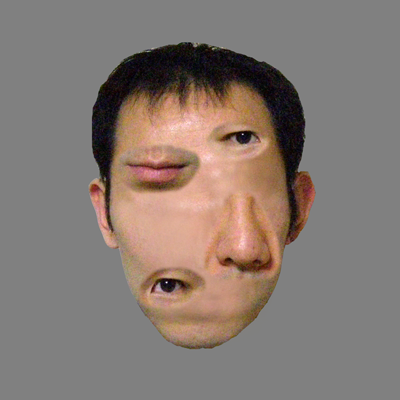

Supplement: S1 File — (ZIP) [file pone.0201192.s002.zip › S1/c03.bmp]

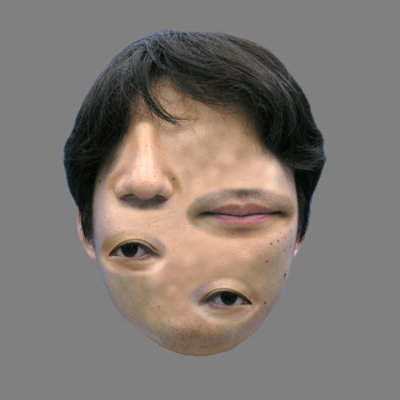

Supplement: S1 File — (ZIP) [file pone.0201192.s002.zip › S1/c04.bmp]

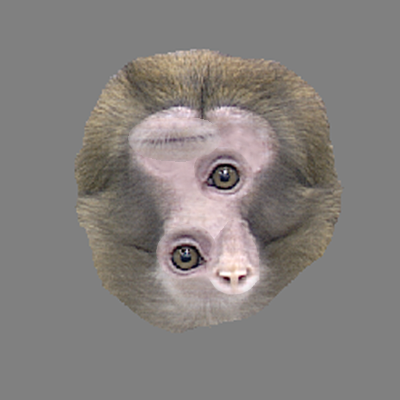

Supplement: S1 File — (ZIP) [file pone.0201192.s002.zip › S1/d01.bmp]

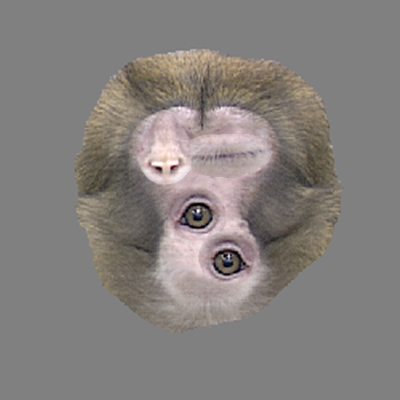

Supplement: S1 File — (ZIP) [file pone.0201192.s002.zip › S1/d02.bmp]

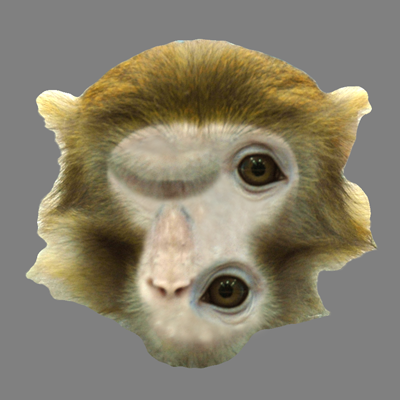

Supplement: S1 File — (ZIP) [file pone.0201192.s002.zip › S1/d03.bmp]

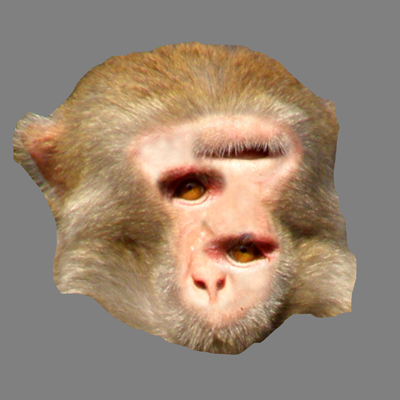

Supplement: S1 File — (ZIP) [file pone.0201192.s002.zip › S1/d04.bmp]

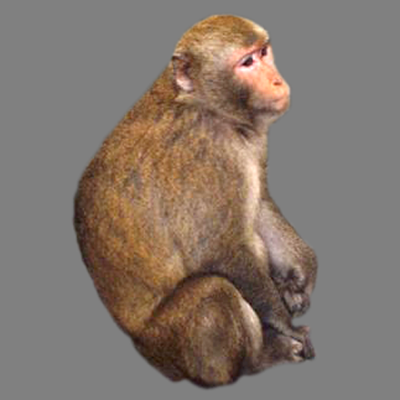

Supplement: S1 File — (ZIP) [file pone.0201192.s002.zip › S1/e01.bmp]

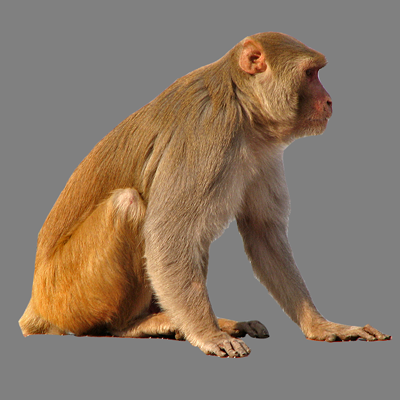

Supplement: S1 File — (ZIP) [file pone.0201192.s002.zip › S1/e02.bmp]

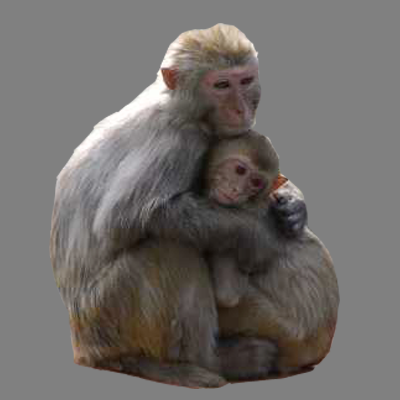

Supplement: S1 File — (ZIP) [file pone.0201192.s002.zip › S1/e03.bmp]

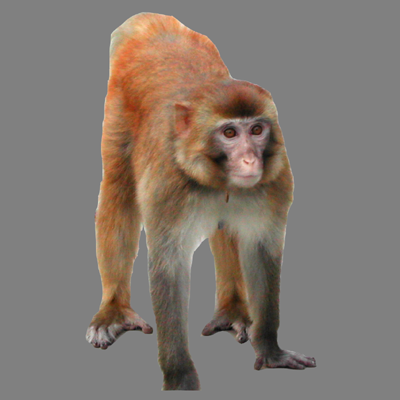

Supplement: S1 File — (ZIP) [file pone.0201192.s002.zip › S1/e04.bmp]

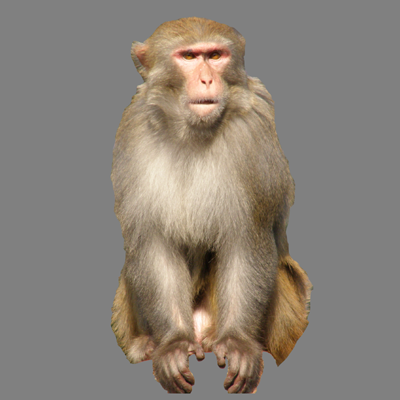

Supplement: S1 File — (ZIP) [file pone.0201192.s002.zip › S1/e05.bmp]

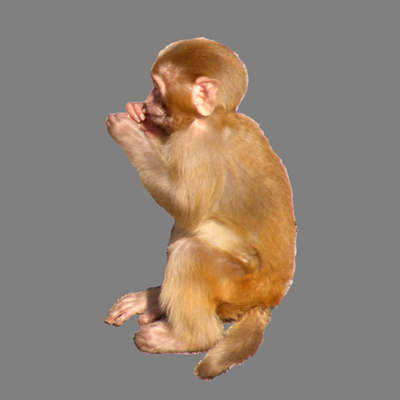

Supplement: S1 File — (ZIP) [file pone.0201192.s002.zip › S1/e06.bmp]

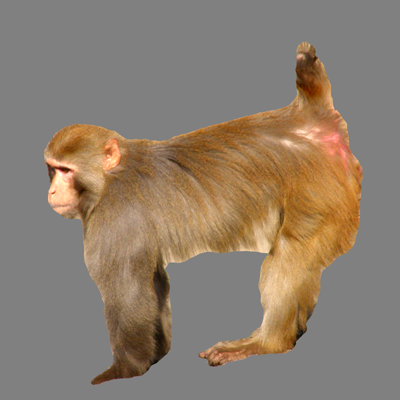

Supplement: S1 File — (ZIP) [file pone.0201192.s002.zip › S1/e07.bmp]

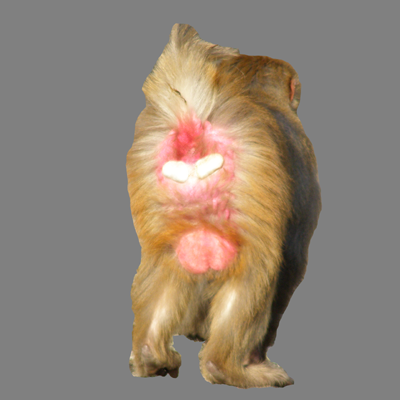

Supplement: S1 File — (ZIP) [file pone.0201192.s002.zip › S1/e08.bmp]

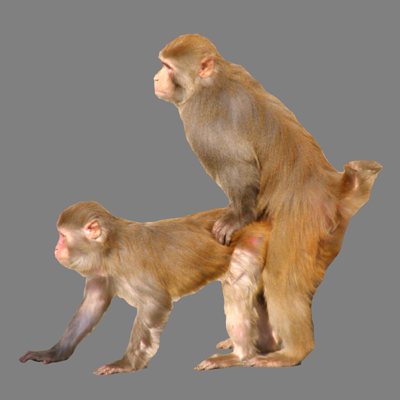

Supplement: S1 File — (ZIP) [file pone.0201192.s002.zip › S1/e09.bmp]

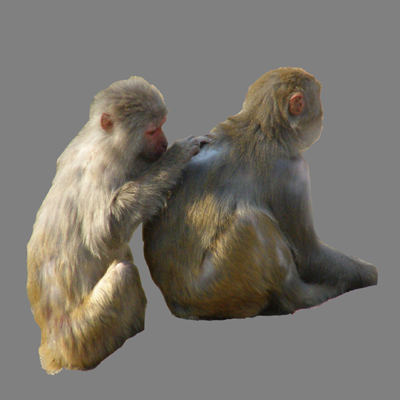

Supplement: S1 File — (ZIP) [file pone.0201192.s002.zip › S1/e10.bmp]

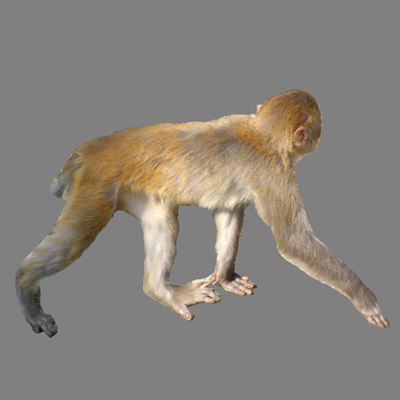

Supplement: S1 File — (ZIP) [file pone.0201192.s002.zip › S1/e11.bmp]

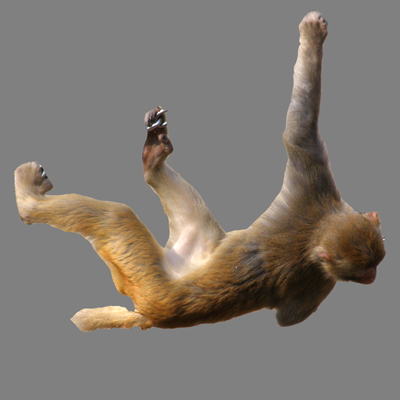

Supplement: S1 File — (ZIP) [file pone.0201192.s002.zip › S1/e12.bmp]

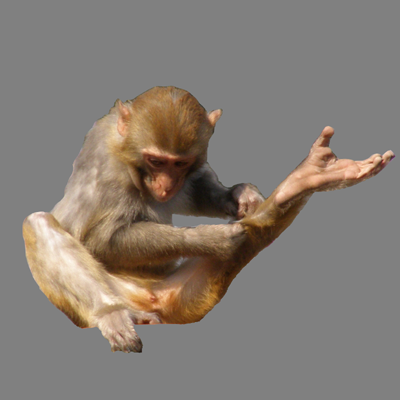

Supplement: S1 File — (ZIP) [file pone.0201192.s002.zip › S1/e13.bmp]

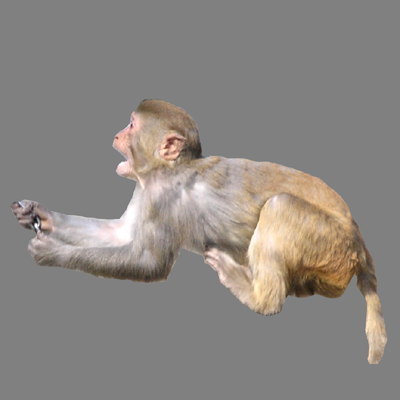

Supplement: S1 File — (ZIP) [file pone.0201192.s002.zip › S1/e14.bmp]

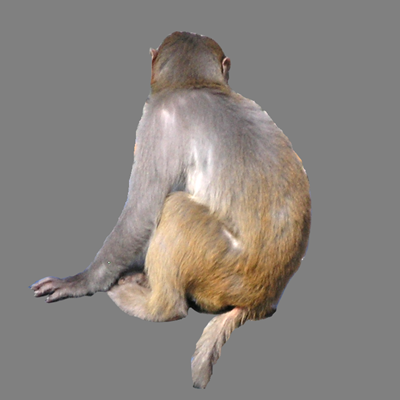

Supplement: S1 File — (ZIP) [file pone.0201192.s002.zip › S1/e15.bmp]

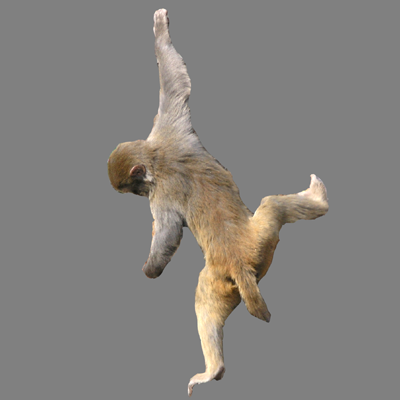

Supplement: S1 File — (ZIP) [file pone.0201192.s002.zip › S1/e16.bmp]

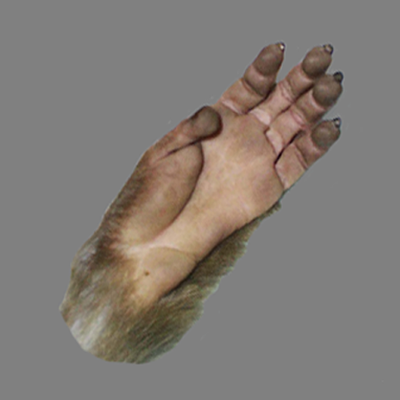

Supplement: S1 File — (ZIP) [file pone.0201192.s002.zip › S1/f01.bmp]

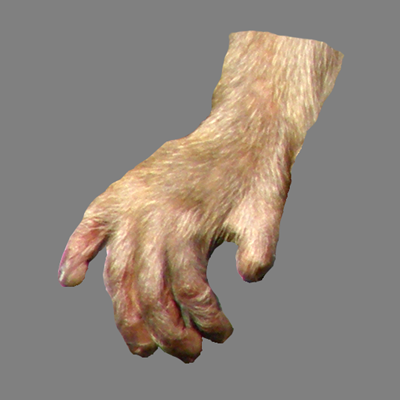

Supplement: S1 File — (ZIP) [file pone.0201192.s002.zip › S1/f02.bmp]

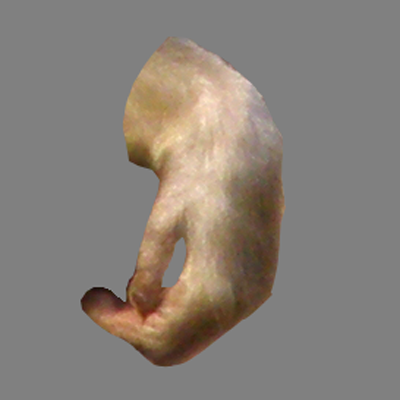

Supplement: S1 File — (ZIP) [file pone.0201192.s002.zip › S1/f03.bmp]

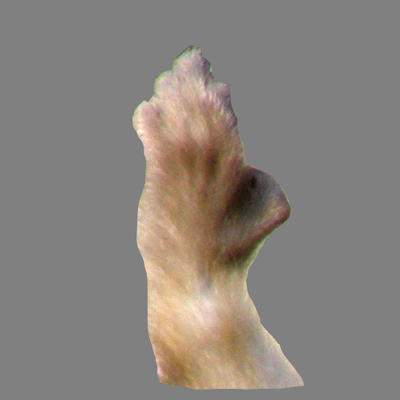

Supplement: S1 File — (ZIP) [file pone.0201192.s002.zip › S1/f04.bmp]

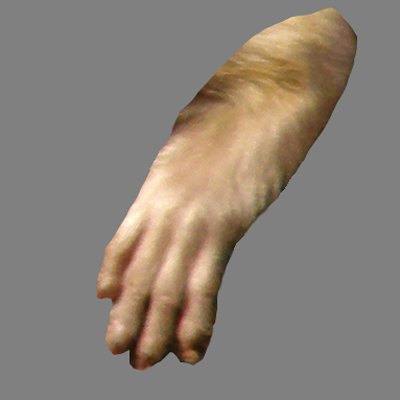

Supplement: S1 File — (ZIP) [file pone.0201192.s002.zip › S1/f05.bmp]

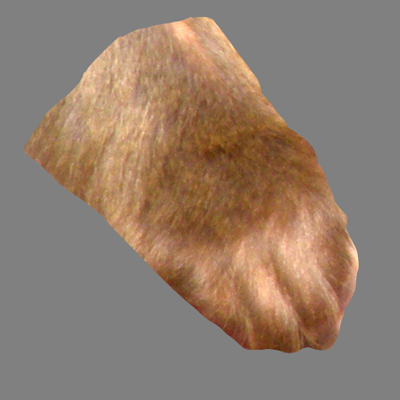

Supplement: S1 File — (ZIP) [file pone.0201192.s002.zip › S1/f06.bmp]

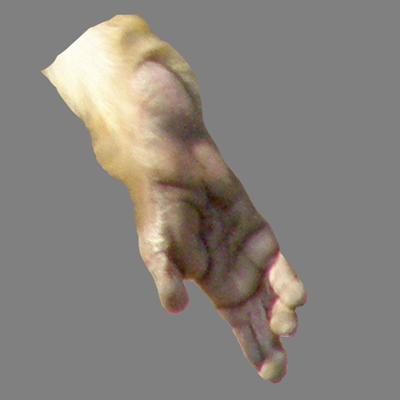

Supplement: S1 File — (ZIP) [file pone.0201192.s002.zip › S1/f07.bmp]

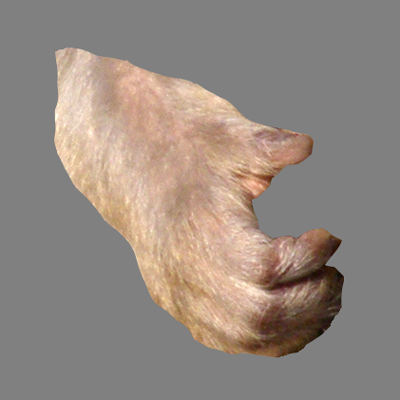

Supplement: S1 File — (ZIP) [file pone.0201192.s002.zip › S1/f08.bmp]

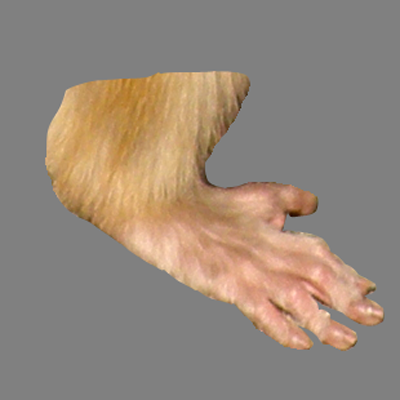

Supplement: S1 File — (ZIP) [file pone.0201192.s002.zip › S1/f09.bmp]

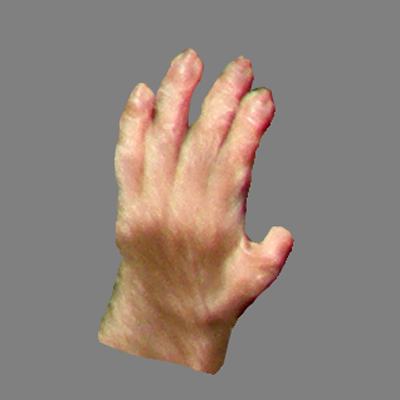

Supplement: S1 File — (ZIP) [file pone.0201192.s002.zip › S1/f10.bmp]

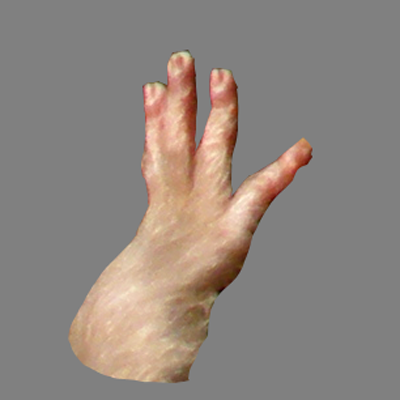

Supplement: S1 File — (ZIP) [file pone.0201192.s002.zip › S1/f11.bmp]

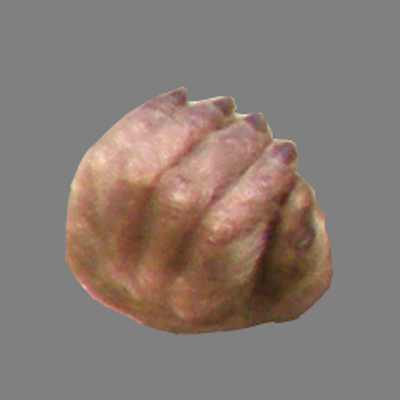

Supplement: S1 File — (ZIP) [file pone.0201192.s002.zip › S1/f12.bmp]

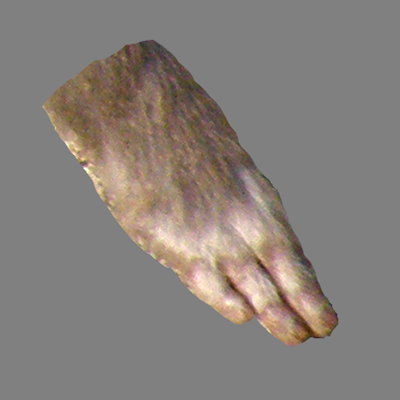

Supplement: S1 File — (ZIP) [file pone.0201192.s002.zip › S1/f13.bmp]

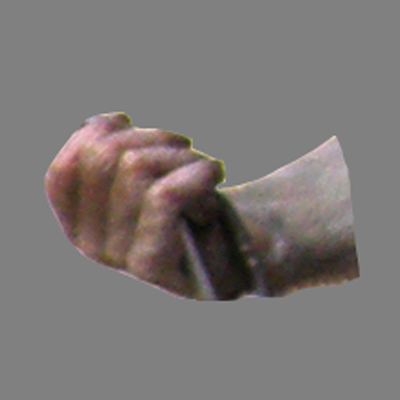

Supplement: S1 File — (ZIP) [file pone.0201192.s002.zip › S1/f14.bmp]

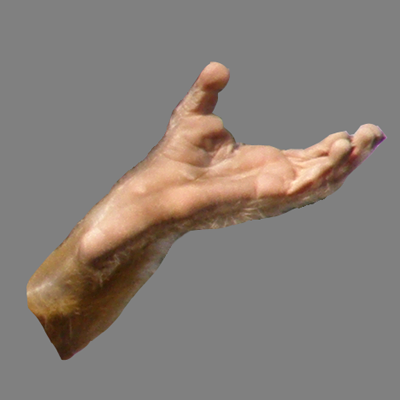

Supplement: S1 File — (ZIP) [file pone.0201192.s002.zip › S1/f15.bmp]

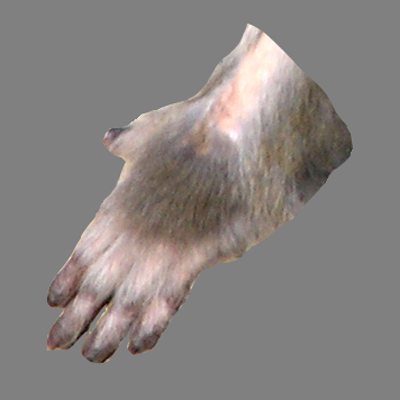

Supplement: S1 File — (ZIP) [file pone.0201192.s002.zip › S1/f16.bmp]

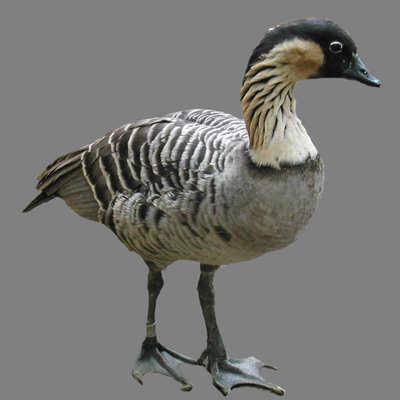

Supplement: S1 File — (ZIP) [file pone.0201192.s002.zip › S1/g01.bmp]

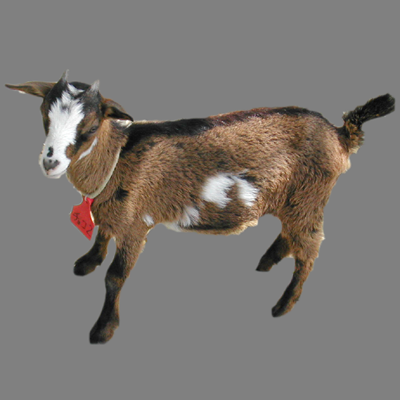

Supplement: S1 File — (ZIP) [file pone.0201192.s002.zip › S1/g02.bmp]

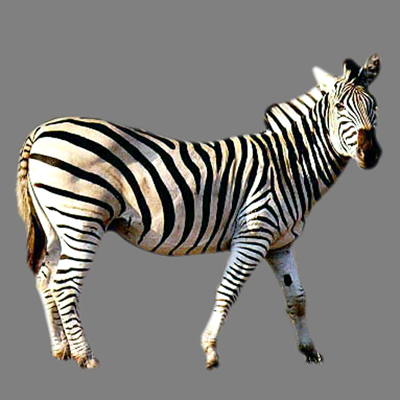

Supplement: S1 File — (ZIP) [file pone.0201192.s002.zip › S1/g03.bmp]

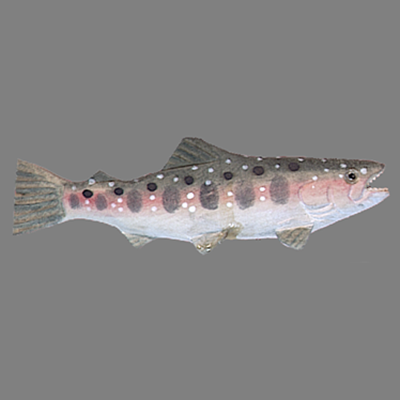

Supplement: S1 File — (ZIP) [file pone.0201192.s002.zip › S1/g04.bmp]

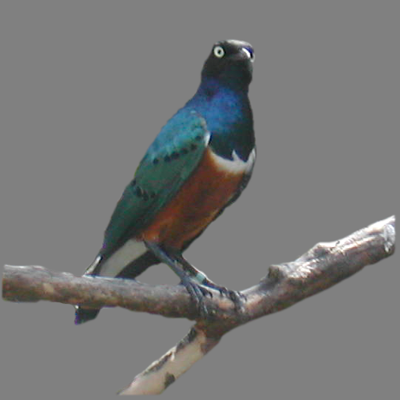

Supplement: S1 File — (ZIP) [file pone.0201192.s002.zip › S1/g05.bmp]

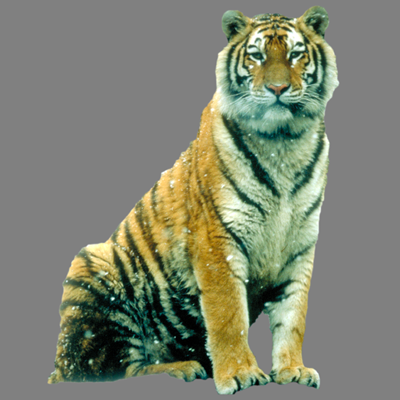

Supplement: S1 File — (ZIP) [file pone.0201192.s002.zip › S1/g06.bmp]

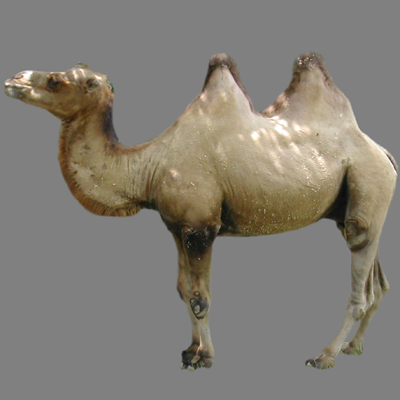

Supplement: S1 File — (ZIP) [file pone.0201192.s002.zip › S1/g07.bmp]

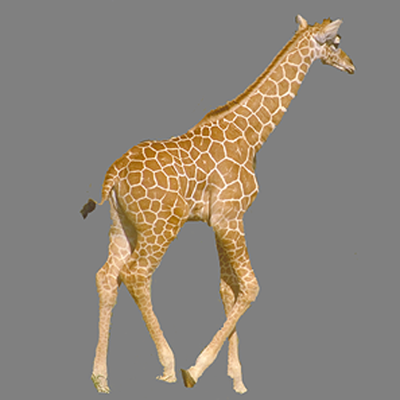

Supplement: S1 File — (ZIP) [file pone.0201192.s002.zip › S1/g08.bmp]

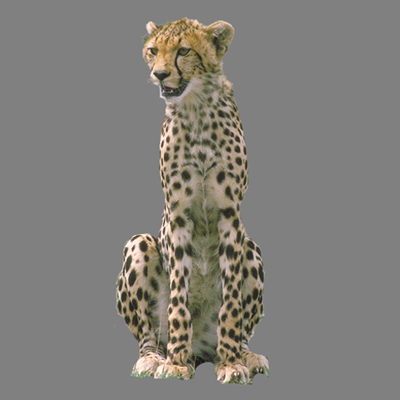

Supplement: S1 File — (ZIP) [file pone.0201192.s002.zip › S1/g09.bmp]

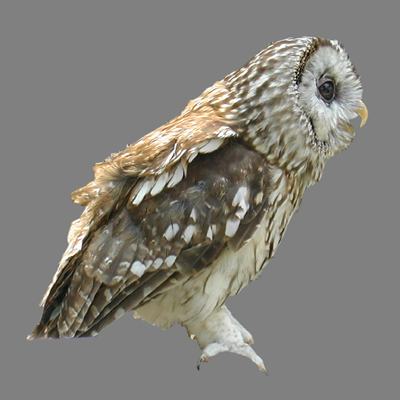

Supplement: S1 File — (ZIP) [file pone.0201192.s002.zip › S1/g10.bmp]

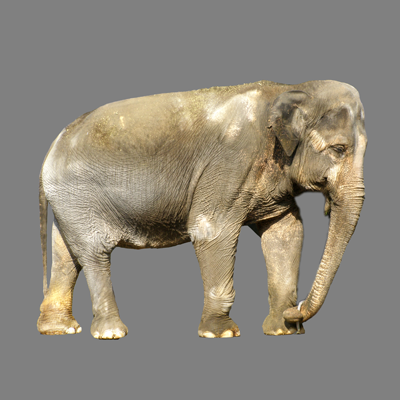

Supplement: S1 File — (ZIP) [file pone.0201192.s002.zip › S1/g11.bmp]

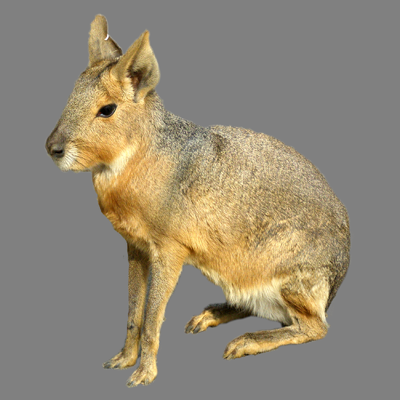

Supplement: S1 File — (ZIP) [file pone.0201192.s002.zip › S1/g12.bmp]

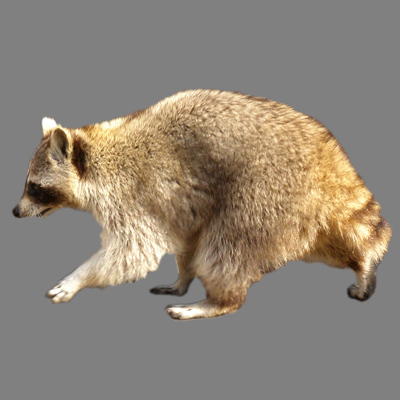

Supplement: S1 File — (ZIP) [file pone.0201192.s002.zip › S1/g13.bmp]

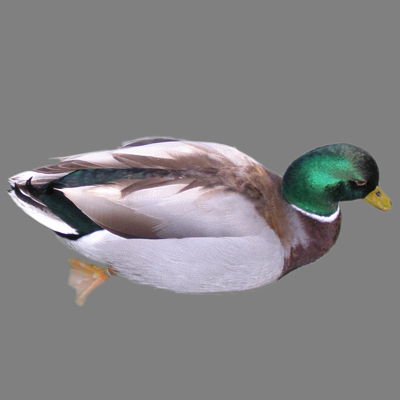

Supplement: S1 File — (ZIP) [file pone.0201192.s002.zip › S1/g14.bmp]

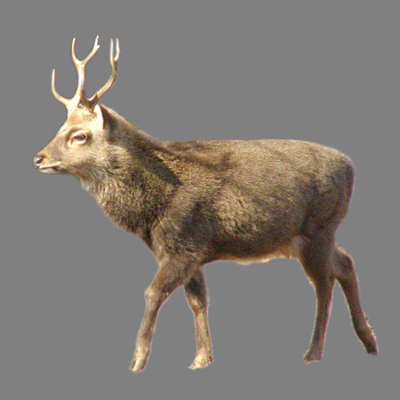

Supplement: S1 File — (ZIP) [file pone.0201192.s002.zip › S1/g15.bmp]

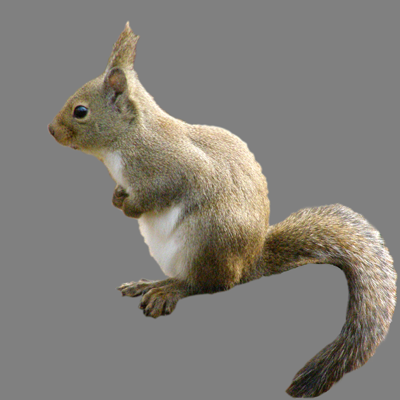

Supplement: S1 File — (ZIP) [file pone.0201192.s002.zip › S1/g16.bmp]

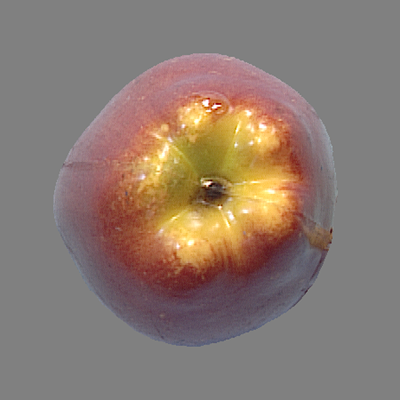

Supplement: S1 File — (ZIP) [file pone.0201192.s002.zip › S1/h01.bmp]

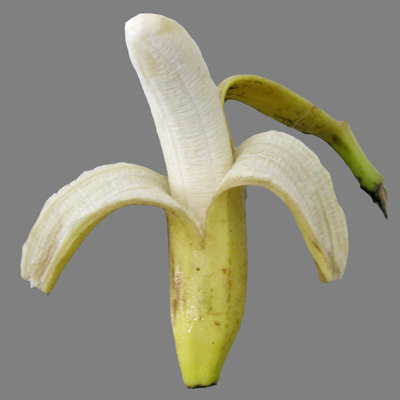

Supplement: S1 File — (ZIP) [file pone.0201192.s002.zip › S1/h02.bmp]

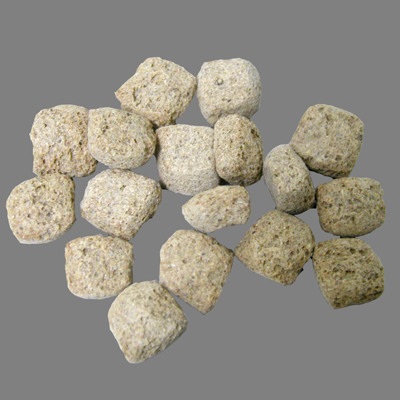

Supplement: S1 File — (ZIP) [file pone.0201192.s002.zip › S1/h03.bmp]

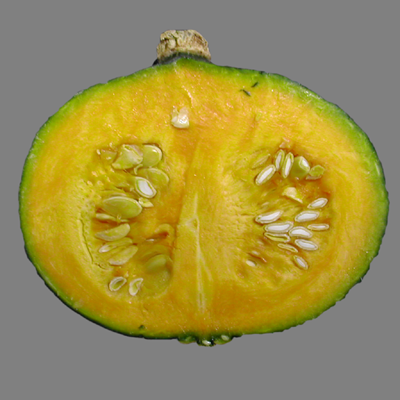

Supplement: S1 File — (ZIP) [file pone.0201192.s002.zip › S1/h04.bmp]

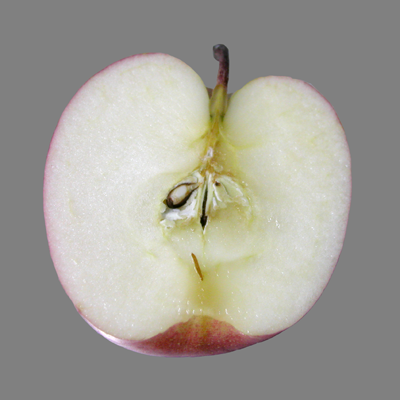

Supplement: S1 File — (ZIP) [file pone.0201192.s002.zip › S1/h05.bmp]

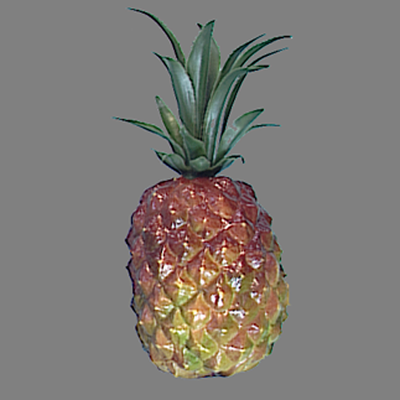

Supplement: S1 File — (ZIP) [file pone.0201192.s002.zip › S1/h06.bmp]

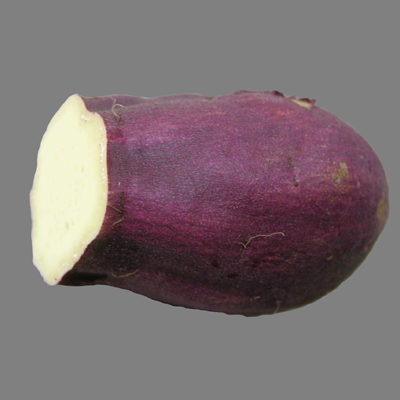

Supplement: S1 File — (ZIP) [file pone.0201192.s002.zip › S1/h07.bmp]

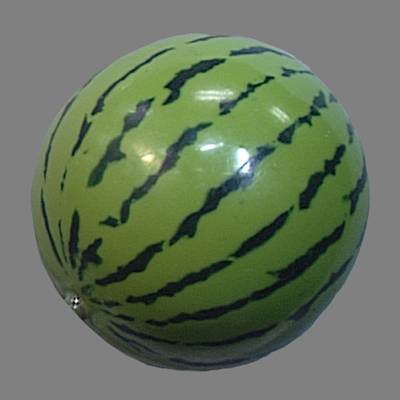

Supplement: S1 File — (ZIP) [file pone.0201192.s002.zip › S1/h08.bmp]

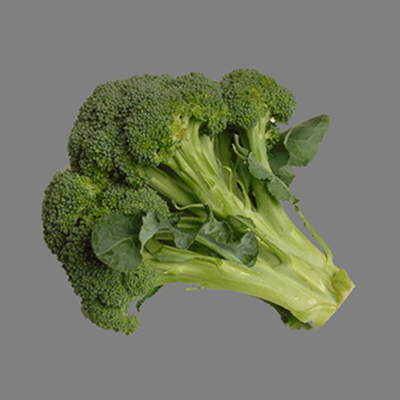

Supplement: S1 File — (ZIP) [file pone.0201192.s002.zip › S1/h09.bmp]

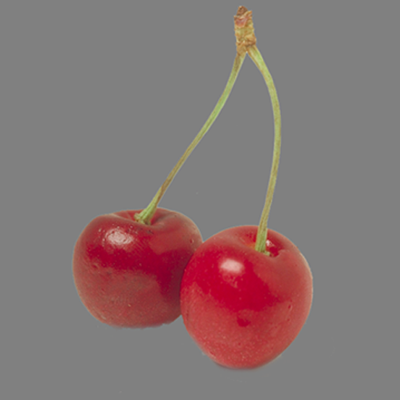

Supplement: S1 File — (ZIP) [file pone.0201192.s002.zip › S1/h10.bmp]

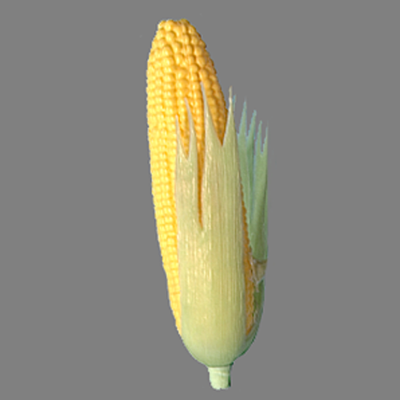

Supplement: S1 File — (ZIP) [file pone.0201192.s002.zip › S1/h11.bmp]

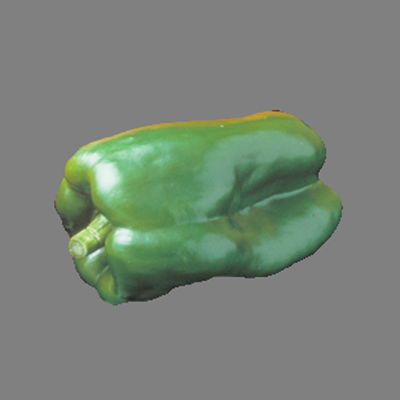

Supplement: S1 File — (ZIP) [file pone.0201192.s002.zip › S1/h12.bmp]

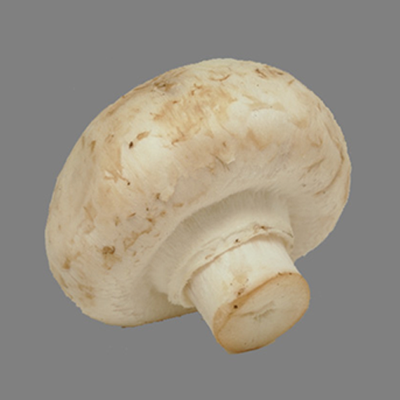

Supplement: S1 File — (ZIP) [file pone.0201192.s002.zip › S1/h13.bmp]

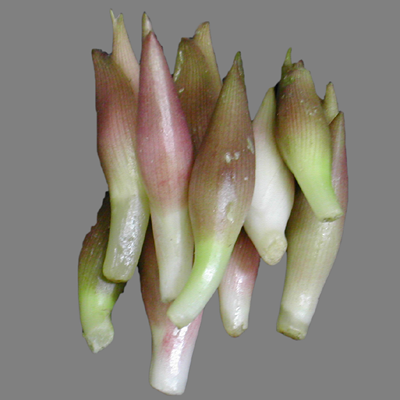

Supplement: S1 File — (ZIP) [file pone.0201192.s002.zip › S1/h14.bmp]

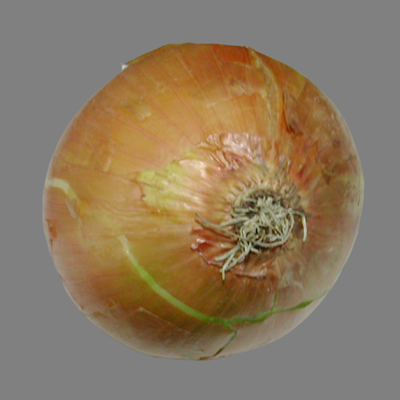

Supplement: S1 File — (ZIP) [file pone.0201192.s002.zip › S1/h15.bmp]

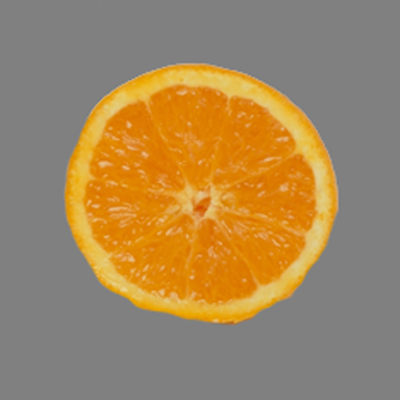

Supplement: S1 File — (ZIP) [file pone.0201192.s002.zip › S1/h16.bmp]

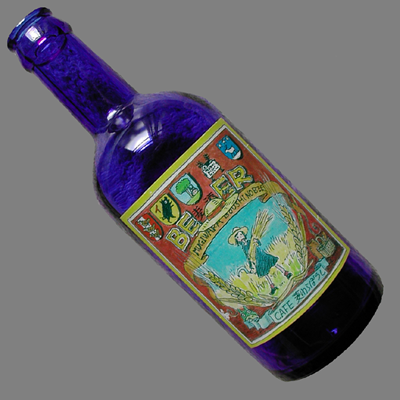

Supplement: S1 File — (ZIP) [file pone.0201192.s002.zip › S1/i01.bmp]

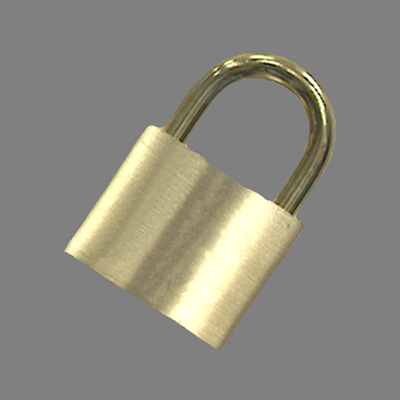

Supplement: S1 File — (ZIP) [file pone.0201192.s002.zip › S1/i02.bmp]

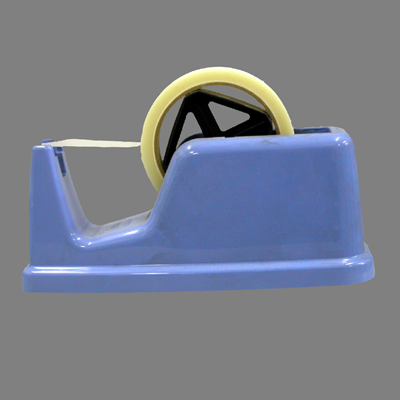

Supplement: S1 File — (ZIP) [file pone.0201192.s002.zip › S1/i03.bmp]

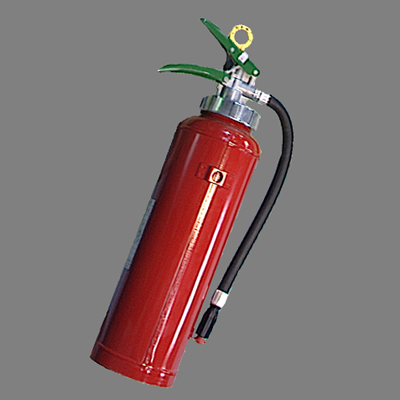

Supplement: S1 File — (ZIP) [file pone.0201192.s002.zip › S1/i04.bmp]

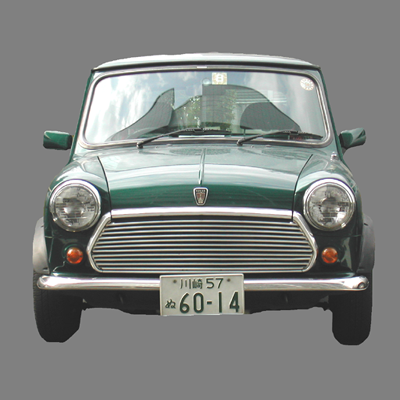

Supplement: S1 File — (ZIP) [file pone.0201192.s002.zip › S1/i05.bmp]

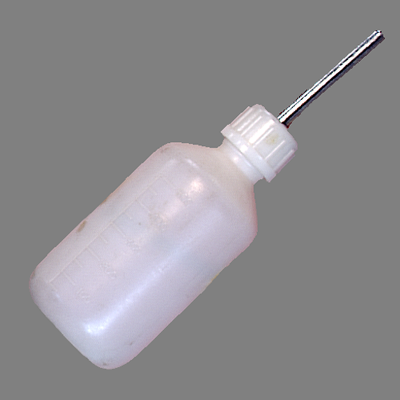

Supplement: S1 File — (ZIP) [file pone.0201192.s002.zip › S1/i06.bmp]

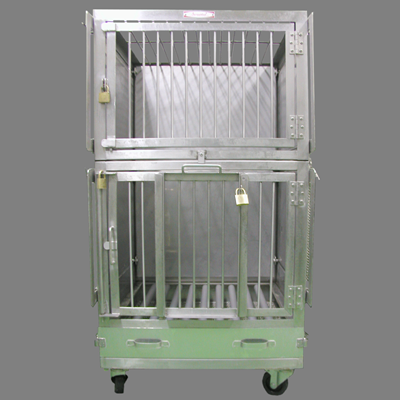

Supplement: S1 File — (ZIP) [file pone.0201192.s002.zip › S1/i07.bmp]

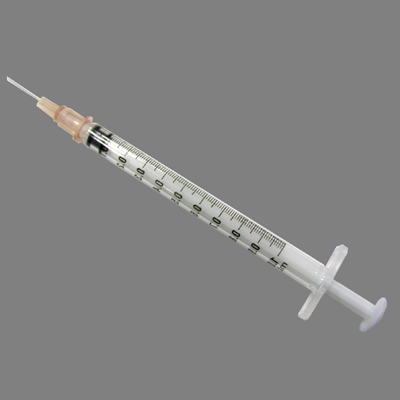

Supplement: S1 File — (ZIP) [file pone.0201192.s002.zip › S1/i08.bmp]

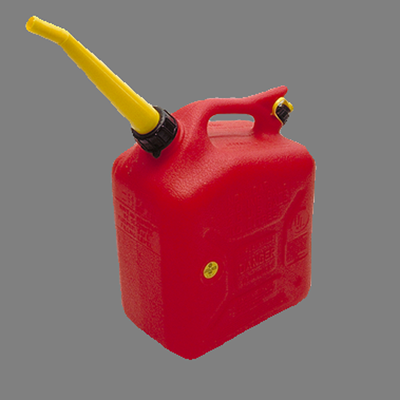

Supplement: S1 File — (ZIP) [file pone.0201192.s002.zip › S1/i09.bmp]

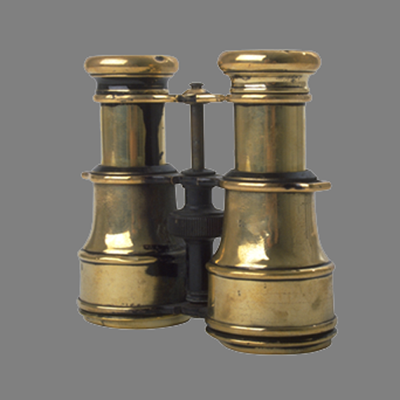

Supplement: S1 File — (ZIP) [file pone.0201192.s002.zip › S1/i10.bmp]

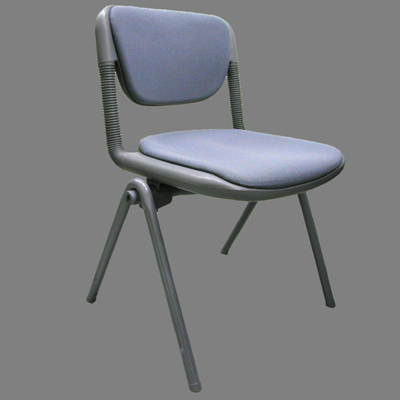

Supplement: S1 File — (ZIP) [file pone.0201192.s002.zip › S1/i11.bmp]

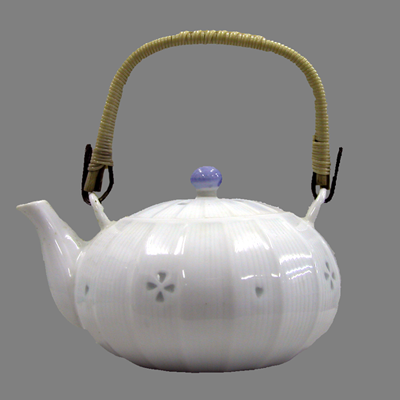

Supplement: S1 File — (ZIP) [file pone.0201192.s002.zip › S1/i12.bmp]
