# Supplementary figures and images for: Searching for visual features that explain response variance of face neurons in inferior temporal cortex (part 4 of 4)
Source: PLoS One. 2018 Sep 20;13(9):e0201192. doi: 10.1371/journal.pone.0201192 (PMC6147465; doi:10.1371/journal.pone.0201192)

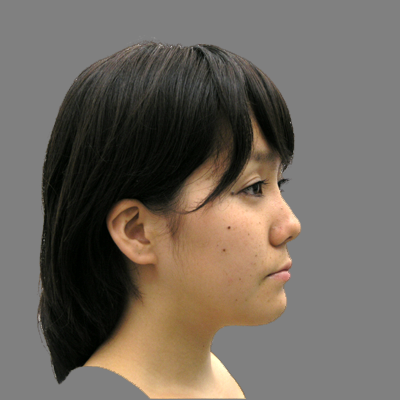

Supplement: S2 File — (ZIP) [file pone.0201192.s003.zip › S2/HH08_03.png]

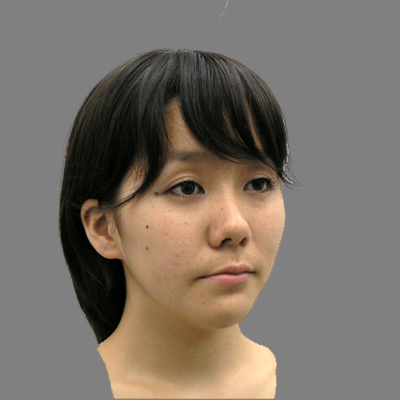

Supplement: S2 File — (ZIP) [file pone.0201192.s003.zip › S2/HH08_05.png]

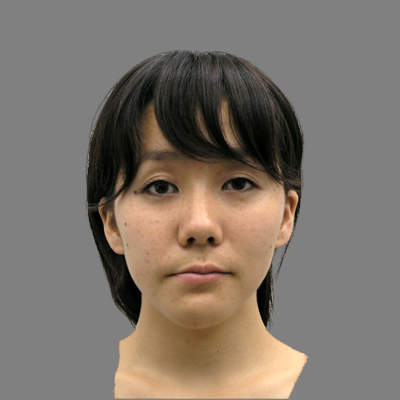

Supplement: S2 File — (ZIP) [file pone.0201192.s003.zip › S2/HH08_06.png]

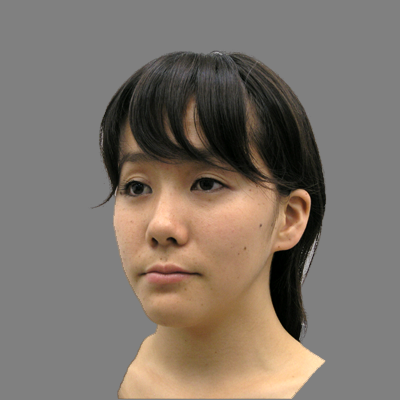

Supplement: S2 File — (ZIP) [file pone.0201192.s003.zip › S2/HH08_07.png]
