# Supplementary figures and images for: Searching for visual features that explain response variance of face neurons in inferior temporal cortex (part 3 of 4)
Source: PLoS One. 2018 Sep 20;13(9):e0201192. doi: 10.1371/journal.pone.0201192 (PMC6147465; doi:10.1371/journal.pone.0201192)

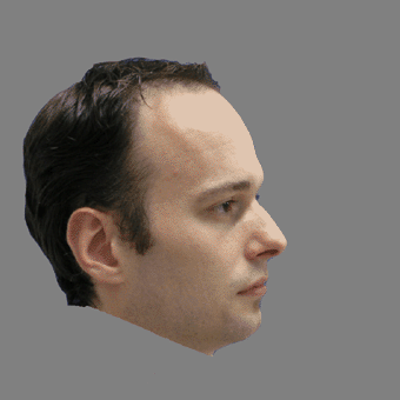

Supplement: S2 File — (ZIP) [file pone.0201192.s003.zip › S2/Hf113.png]

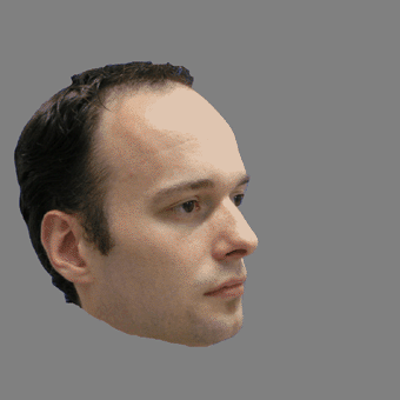

Supplement: S2 File — (ZIP) [file pone.0201192.s003.zip › S2/Hf115.png]

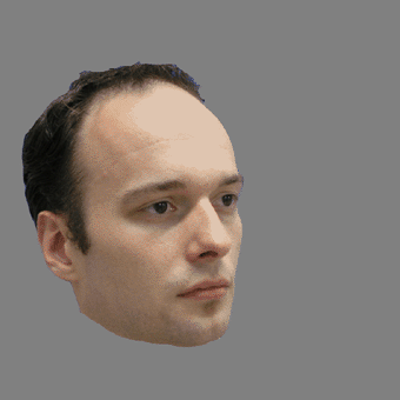

Supplement: S2 File — (ZIP) [file pone.0201192.s003.zip › S2/Hf117.png]

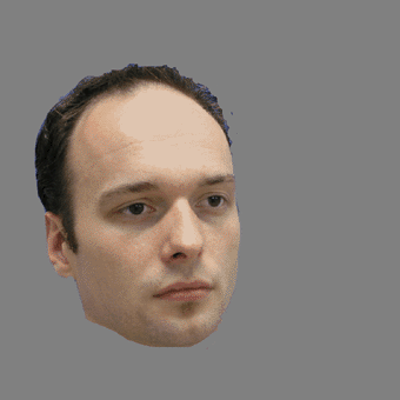

Supplement: S2 File — (ZIP) [file pone.0201192.s003.zip › S2/Hf119.png]

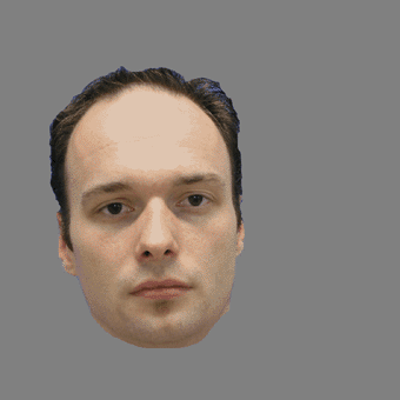

Supplement: S2 File — (ZIP) [file pone.0201192.s003.zip › S2/Hf121.png]

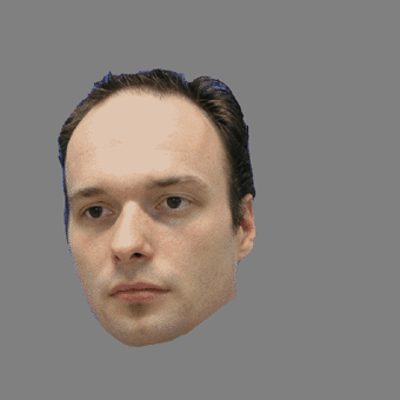

Supplement: S2 File — (ZIP) [file pone.0201192.s003.zip › S2/Hf123.png]

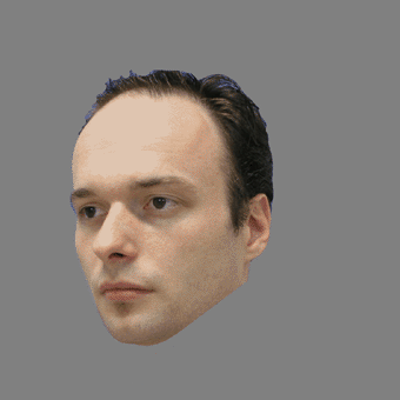

Supplement: S2 File — (ZIP) [file pone.0201192.s003.zip › S2/Hf125.png]

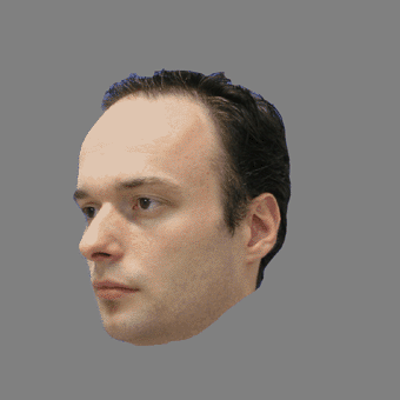

Supplement: S2 File — (ZIP) [file pone.0201192.s003.zip › S2/Hf127.png]

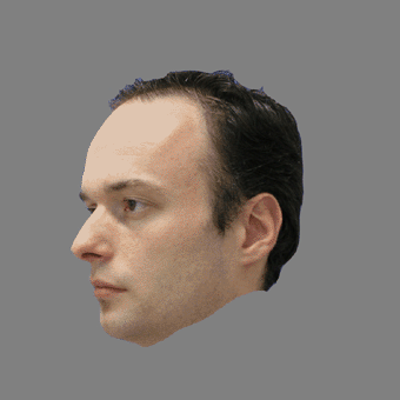

Supplement: S2 File — (ZIP) [file pone.0201192.s003.zip › S2/Hf129.png]

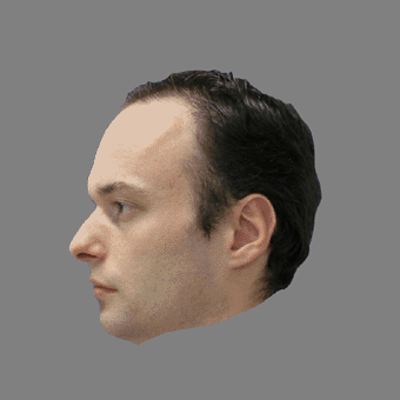

Supplement: S2 File — (ZIP) [file pone.0201192.s003.zip › S2/Hf131.png]

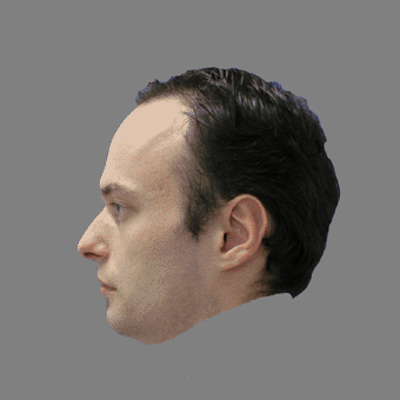

Supplement: S2 File — (ZIP) [file pone.0201192.s003.zip › S2/Hf133.png]

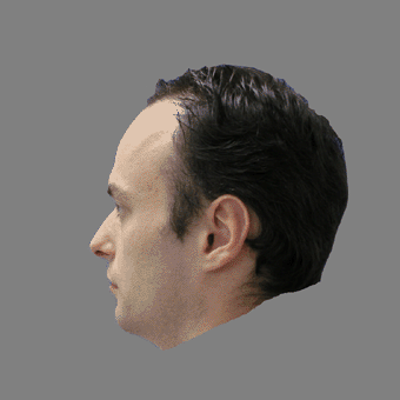

Supplement: S2 File — (ZIP) [file pone.0201192.s003.zip › S2/Hf135.png]

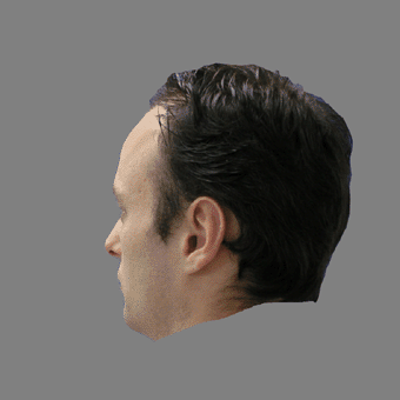

Supplement: S2 File — (ZIP) [file pone.0201192.s003.zip › S2/Hf137.png]

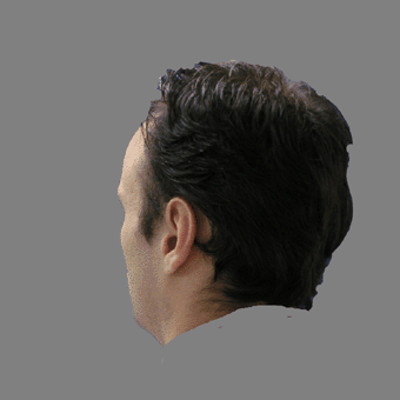

Supplement: S2 File — (ZIP) [file pone.0201192.s003.zip › S2/Hf139.png]

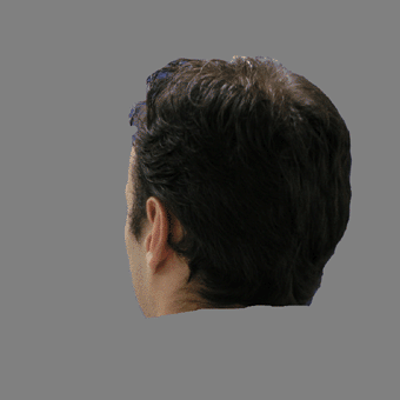

Supplement: S2 File — (ZIP) [file pone.0201192.s003.zip › S2/Hf141.png]

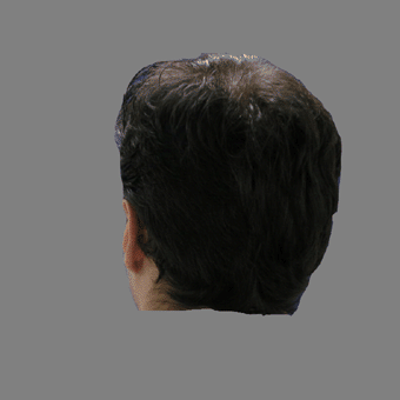

Supplement: S2 File — (ZIP) [file pone.0201192.s003.zip › S2/Hf143.png]

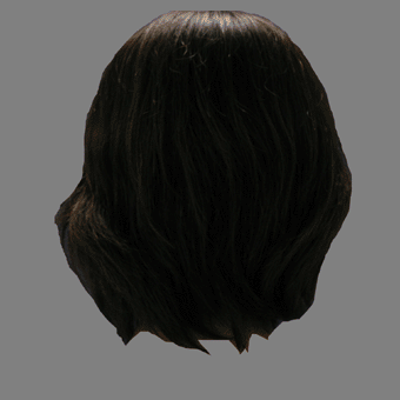

Supplement: S2 File — (ZIP) [file pone.0201192.s003.zip › S2/Hf145.png]

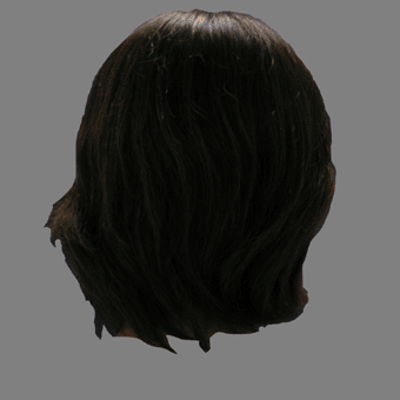

Supplement: S2 File — (ZIP) [file pone.0201192.s003.zip › S2/Hf147.png]

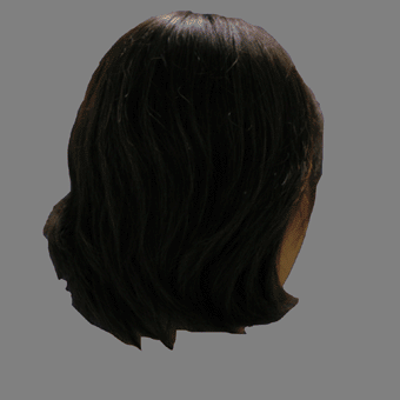

Supplement: S2 File — (ZIP) [file pone.0201192.s003.zip › S2/Hf149.png]

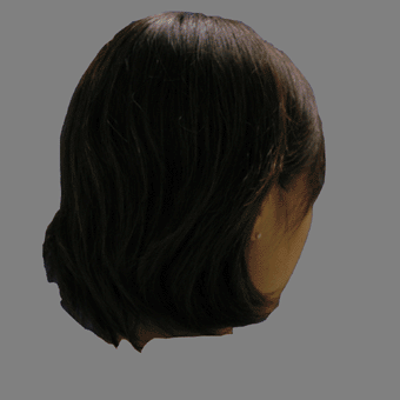

Supplement: S2 File — (ZIP) [file pone.0201192.s003.zip › S2/Hf151.png]

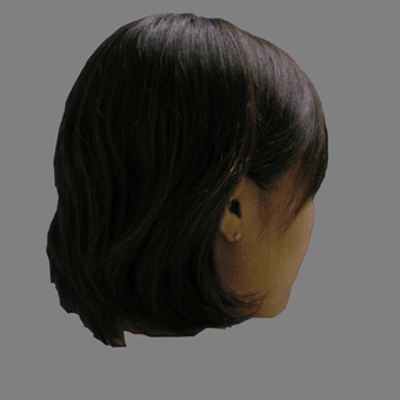

Supplement: S2 File — (ZIP) [file pone.0201192.s003.zip › S2/Hf153.png]

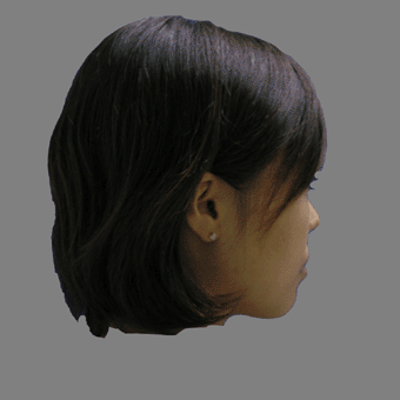

Supplement: S2 File — (ZIP) [file pone.0201192.s003.zip › S2/Hf155.png]

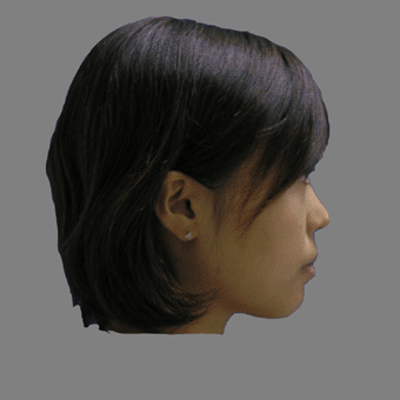

Supplement: S2 File — (ZIP) [file pone.0201192.s003.zip › S2/Hf157.png]

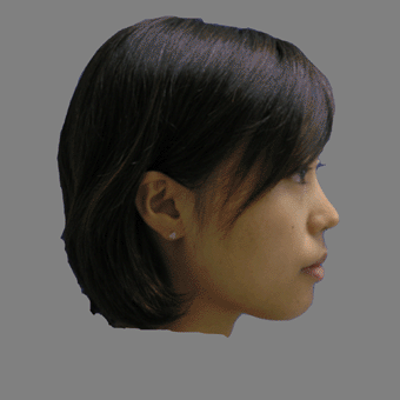

Supplement: S2 File — (ZIP) [file pone.0201192.s003.zip › S2/Hf159.png]

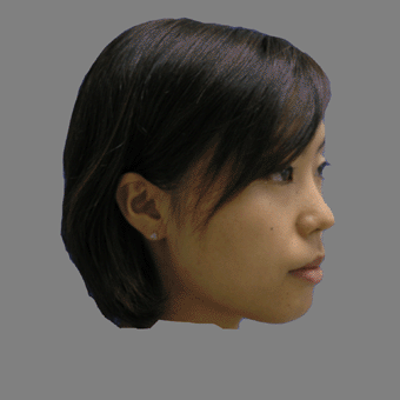

Supplement: S2 File — (ZIP) [file pone.0201192.s003.zip › S2/Hf161.png]

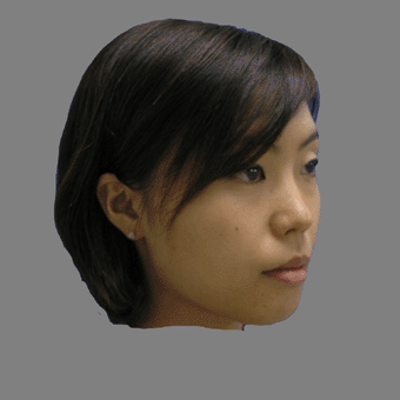

Supplement: S2 File — (ZIP) [file pone.0201192.s003.zip › S2/Hf163.png]

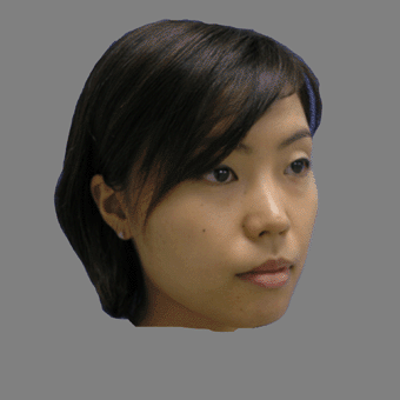

Supplement: S2 File — (ZIP) [file pone.0201192.s003.zip › S2/Hf165.png]

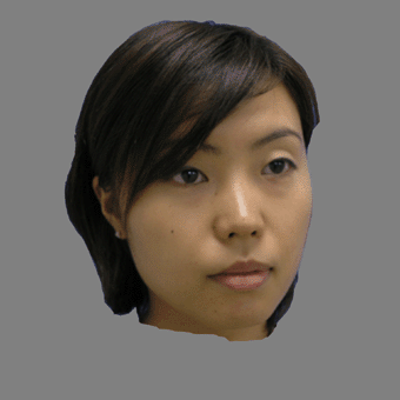

Supplement: S2 File — (ZIP) [file pone.0201192.s003.zip › S2/Hf167.png]

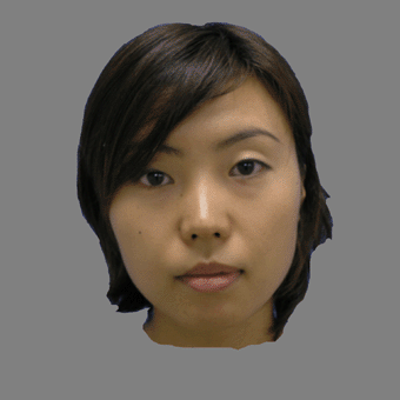

Supplement: S2 File — (ZIP) [file pone.0201192.s003.zip › S2/Hf169.png]

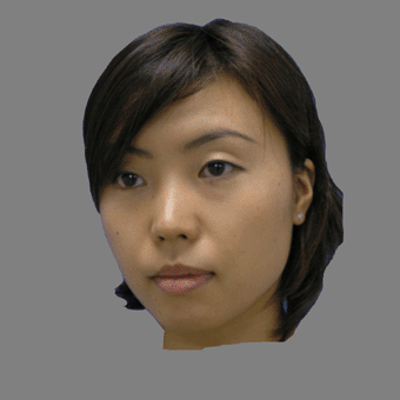

Supplement: S2 File — (ZIP) [file pone.0201192.s003.zip › S2/Hf171.png]

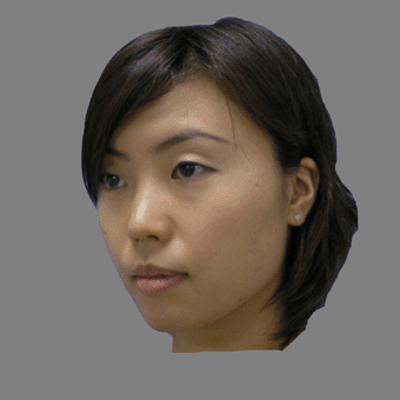

Supplement: S2 File — (ZIP) [file pone.0201192.s003.zip › S2/Hf173.png]

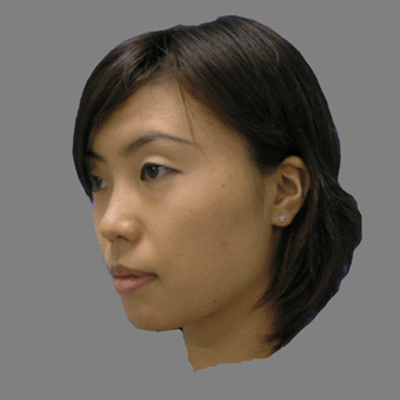

Supplement: S2 File — (ZIP) [file pone.0201192.s003.zip › S2/Hf175.png]

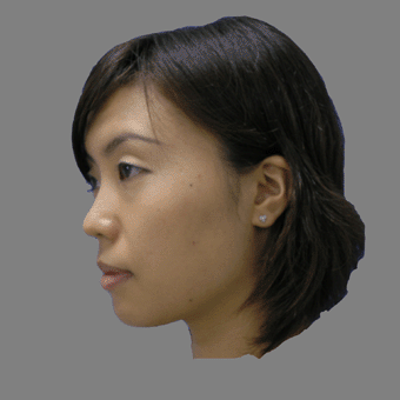

Supplement: S2 File — (ZIP) [file pone.0201192.s003.zip › S2/Hf177.png]

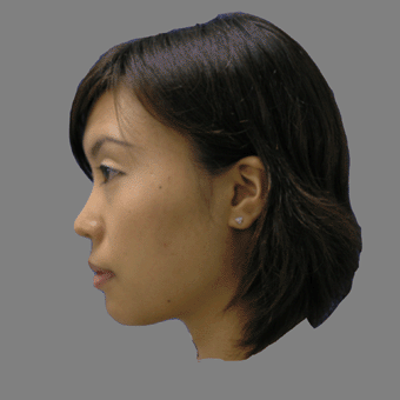

Supplement: S2 File — (ZIP) [file pone.0201192.s003.zip › S2/Hf179.png]

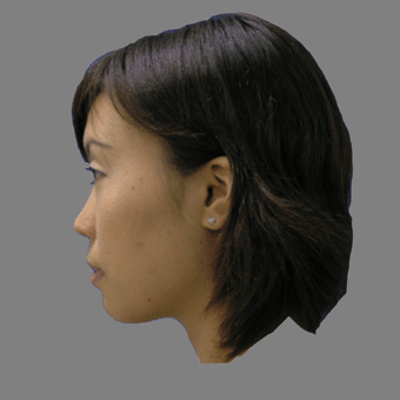

Supplement: S2 File — (ZIP) [file pone.0201192.s003.zip › S2/Hf181.png]

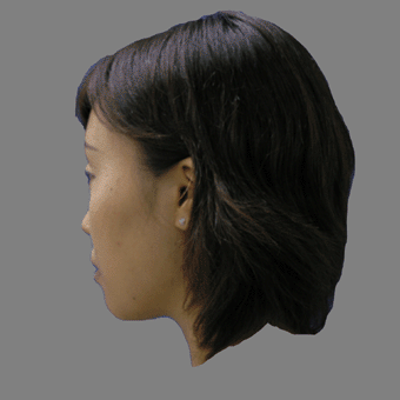

Supplement: S2 File — (ZIP) [file pone.0201192.s003.zip › S2/Hf183.png]

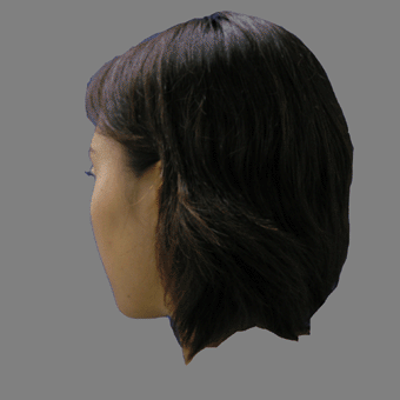

Supplement: S2 File — (ZIP) [file pone.0201192.s003.zip › S2/Hf185.png]

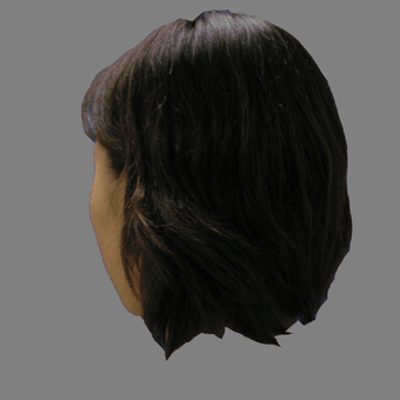

Supplement: S2 File — (ZIP) [file pone.0201192.s003.zip › S2/Hf187.png]

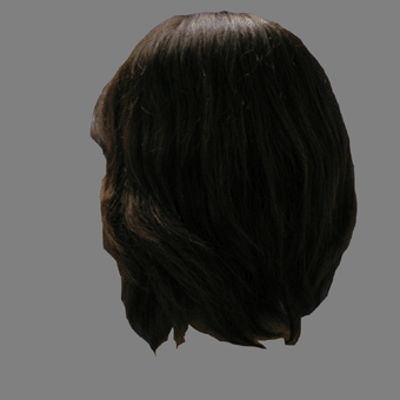

Supplement: S2 File — (ZIP) [file pone.0201192.s003.zip › S2/Hf189.png]

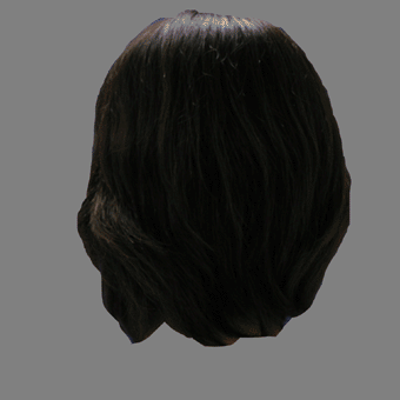

Supplement: S2 File — (ZIP) [file pone.0201192.s003.zip › S2/Hf191.png]

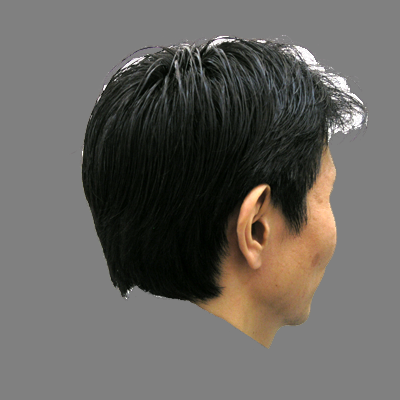

Supplement: S2 File — (ZIP) [file pone.0201192.s003.zip › S2/HH01_01.png]

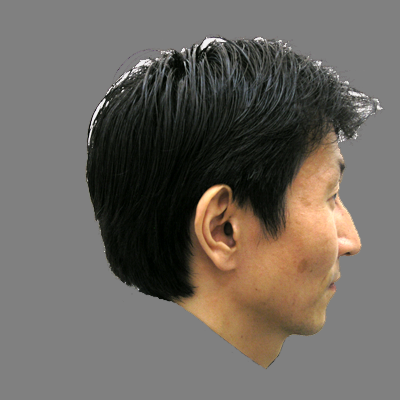

Supplement: S2 File — (ZIP) [file pone.0201192.s003.zip › S2/HH01_02.png]

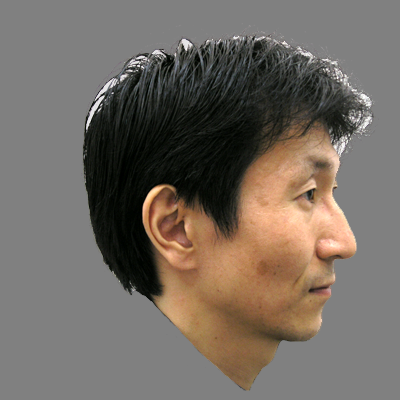

Supplement: S2 File — (ZIP) [file pone.0201192.s003.zip › S2/HH01_03.png]

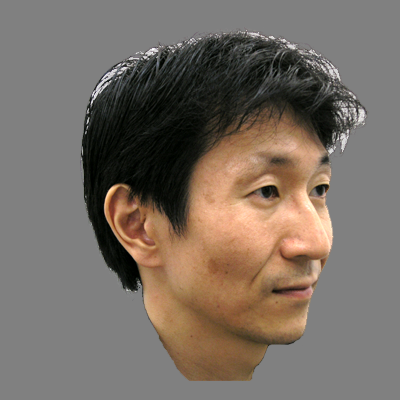

Supplement: S2 File — (ZIP) [file pone.0201192.s003.zip › S2/HH01_04.png]

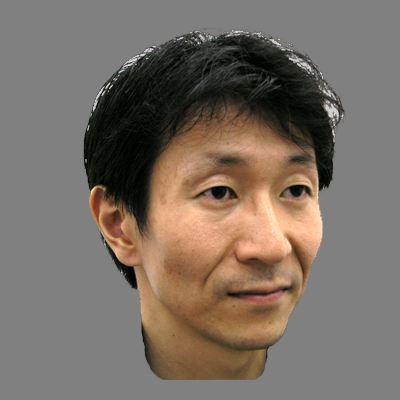

Supplement: S2 File — (ZIP) [file pone.0201192.s003.zip › S2/HH01_05.png]

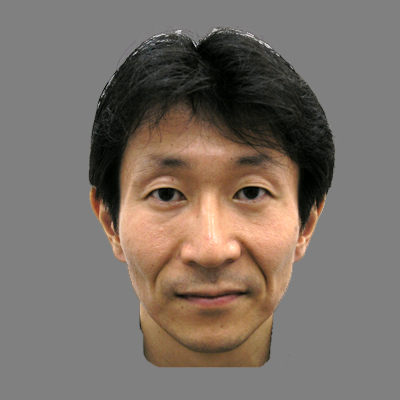

Supplement: S2 File — (ZIP) [file pone.0201192.s003.zip › S2/HH01_06.png]

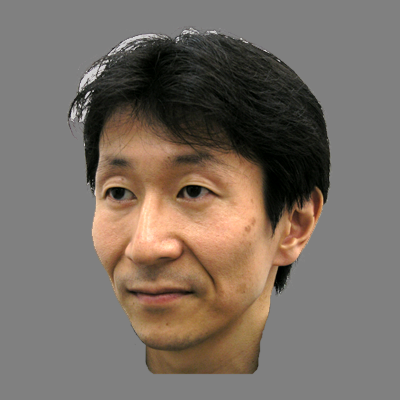

Supplement: S2 File — (ZIP) [file pone.0201192.s003.zip › S2/HH01_07.png]

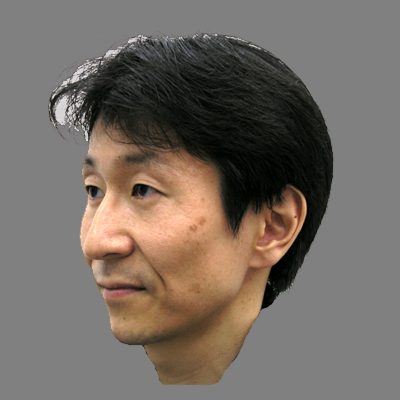

Supplement: S2 File — (ZIP) [file pone.0201192.s003.zip › S2/HH01_08.png]

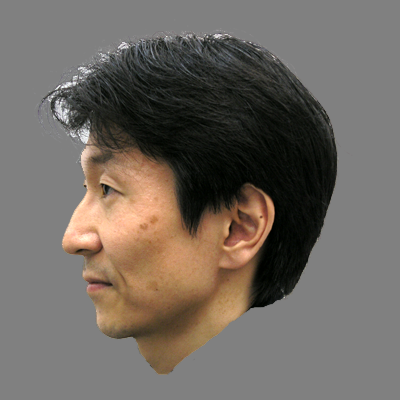

Supplement: S2 File — (ZIP) [file pone.0201192.s003.zip › S2/HH01_09.png]

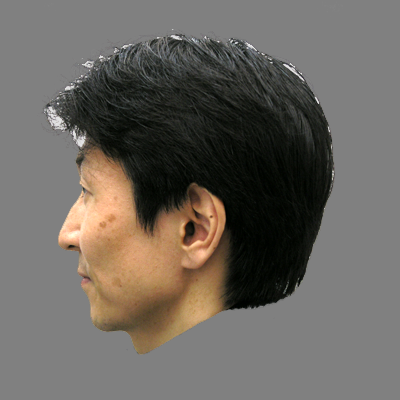

Supplement: S2 File — (ZIP) [file pone.0201192.s003.zip › S2/HH01_10.png]

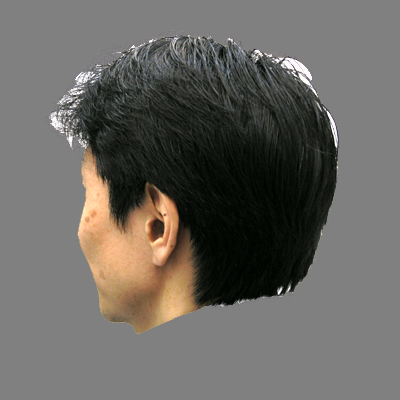

Supplement: S2 File — (ZIP) [file pone.0201192.s003.zip › S2/HH01_11.png]

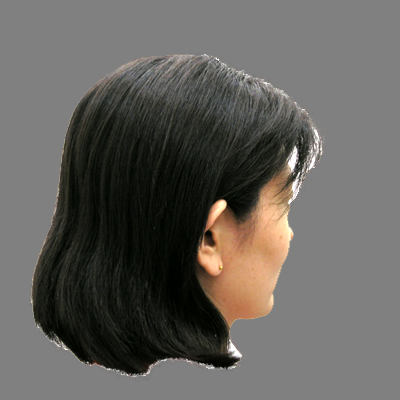

Supplement: S2 File — (ZIP) [file pone.0201192.s003.zip › S2/HH02_01.png]

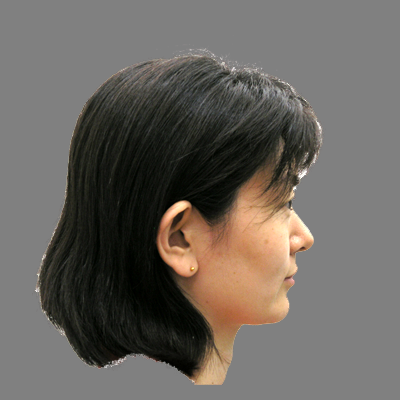

Supplement: S2 File — (ZIP) [file pone.0201192.s003.zip › S2/HH02_02.png]

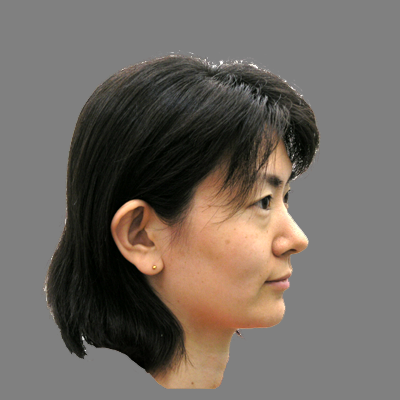

Supplement: S2 File — (ZIP) [file pone.0201192.s003.zip › S2/HH02_03.png]

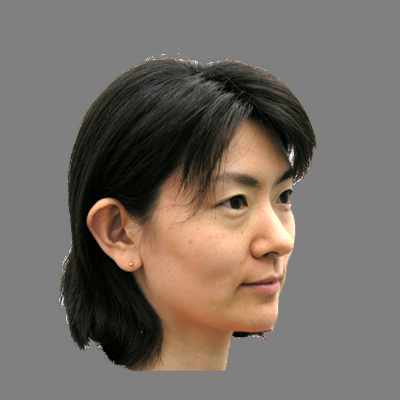

Supplement: S2 File — (ZIP) [file pone.0201192.s003.zip › S2/HH02_04.png]

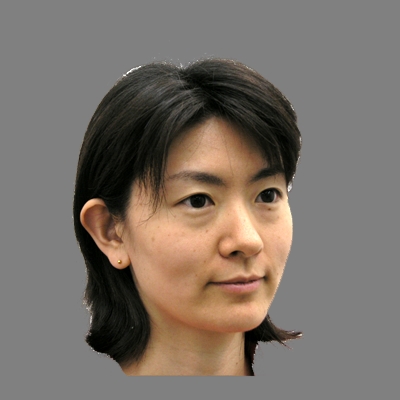

Supplement: S2 File — (ZIP) [file pone.0201192.s003.zip › S2/HH02_05.png]

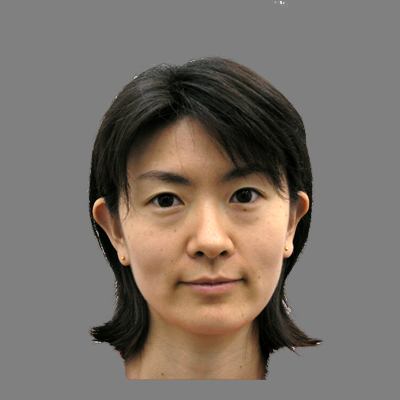

Supplement: S2 File — (ZIP) [file pone.0201192.s003.zip › S2/HH02_06.png]

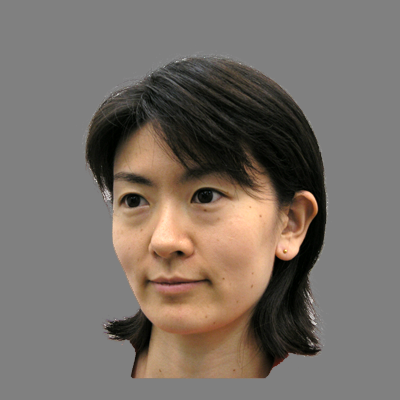

Supplement: S2 File — (ZIP) [file pone.0201192.s003.zip › S2/HH02_07.png]

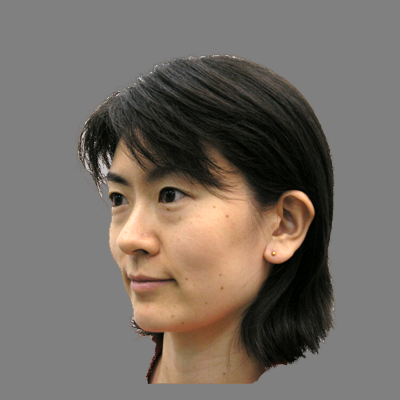

Supplement: S2 File — (ZIP) [file pone.0201192.s003.zip › S2/HH02_08.png]

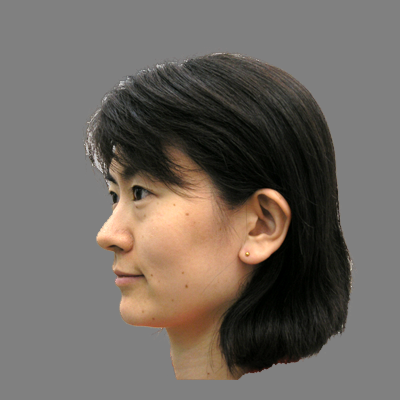

Supplement: S2 File — (ZIP) [file pone.0201192.s003.zip › S2/HH02_09.png]

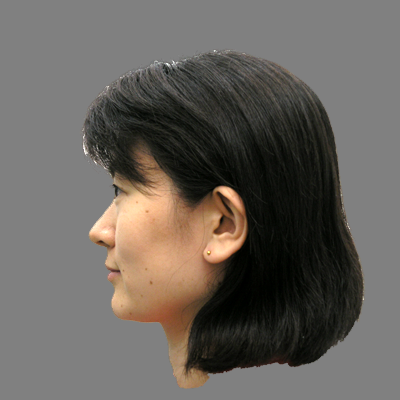

Supplement: S2 File — (ZIP) [file pone.0201192.s003.zip › S2/HH02_10.png]

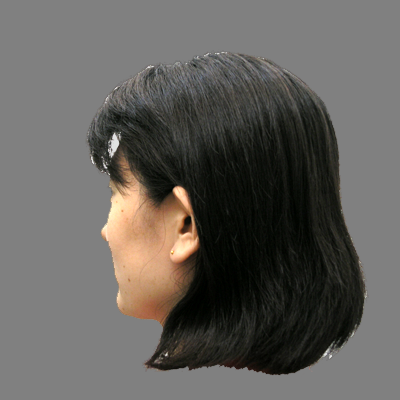

Supplement: S2 File — (ZIP) [file pone.0201192.s003.zip › S2/HH02_11.png]

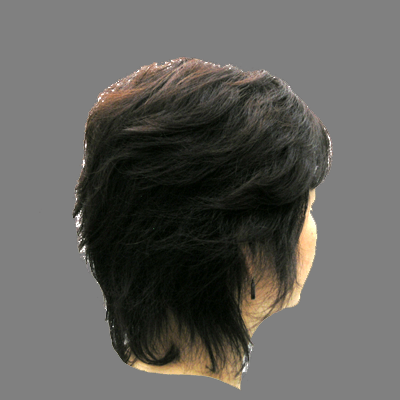

Supplement: S2 File — (ZIP) [file pone.0201192.s003.zip › S2/HH03_01.png]

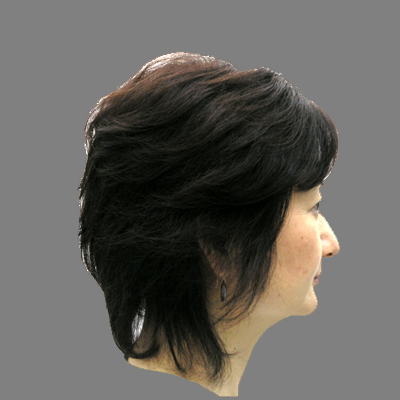

Supplement: S2 File — (ZIP) [file pone.0201192.s003.zip › S2/HH03_02.png]

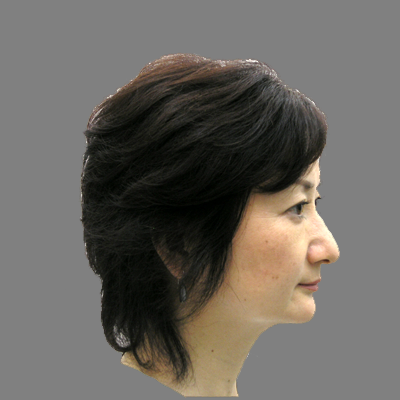

Supplement: S2 File — (ZIP) [file pone.0201192.s003.zip › S2/HH03_03.png]

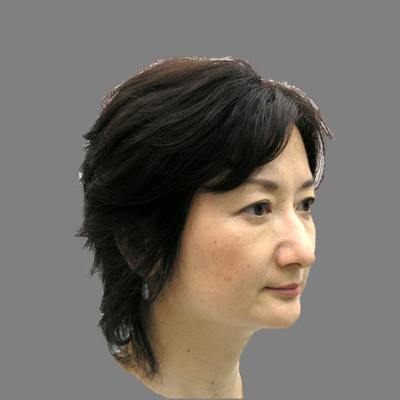

Supplement: S2 File — (ZIP) [file pone.0201192.s003.zip › S2/HH03_04.png]

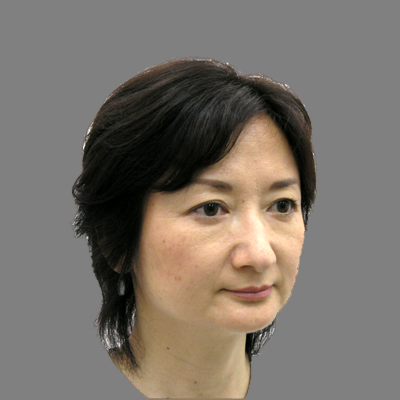

Supplement: S2 File — (ZIP) [file pone.0201192.s003.zip › S2/HH03_05.png]

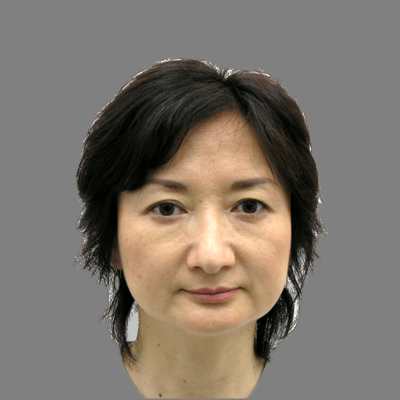

Supplement: S2 File — (ZIP) [file pone.0201192.s003.zip › S2/HH03_06.png]

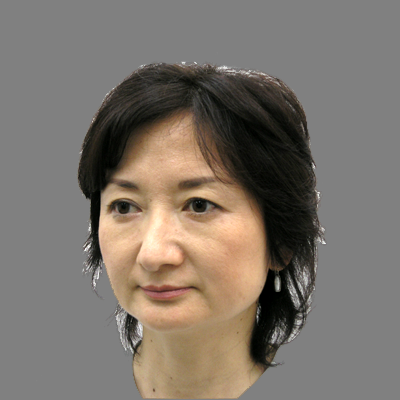

Supplement: S2 File — (ZIP) [file pone.0201192.s003.zip › S2/HH03_07.png]

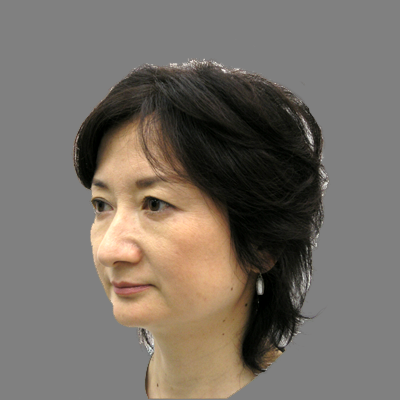

Supplement: S2 File — (ZIP) [file pone.0201192.s003.zip › S2/HH03_08.png]

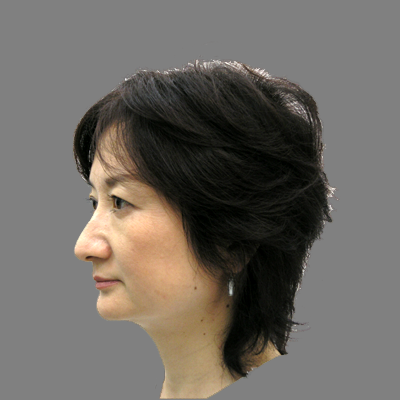

Supplement: S2 File — (ZIP) [file pone.0201192.s003.zip › S2/HH03_09.png]

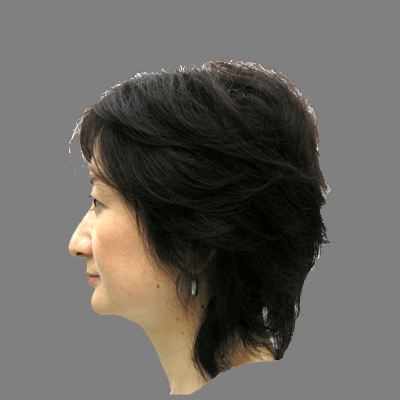

Supplement: S2 File — (ZIP) [file pone.0201192.s003.zip › S2/HH03_10.png]

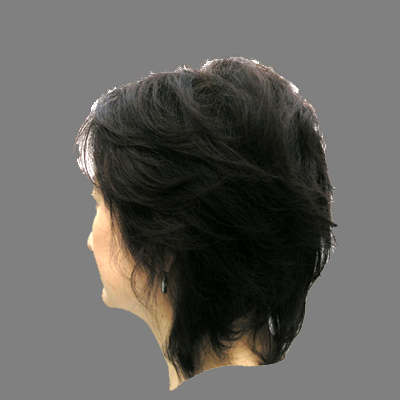

Supplement: S2 File — (ZIP) [file pone.0201192.s003.zip › S2/HH03_11.png]

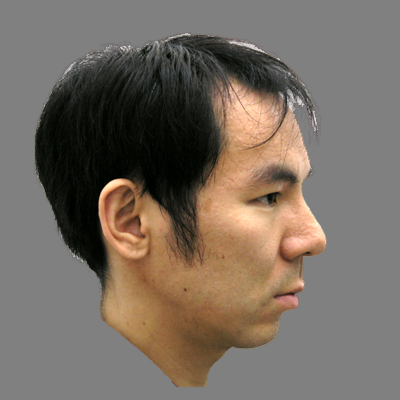

Supplement: S2 File — (ZIP) [file pone.0201192.s003.zip › S2/HH04_03.png]

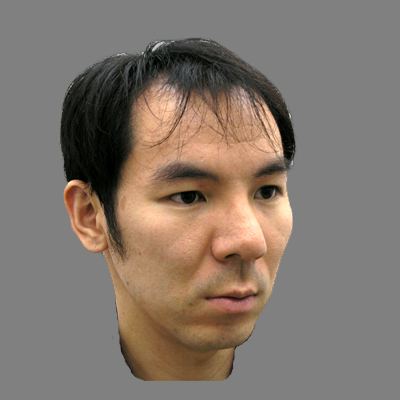

Supplement: S2 File — (ZIP) [file pone.0201192.s003.zip › S2/HH04_05.png]

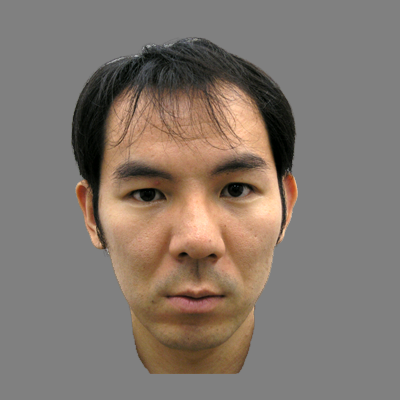

Supplement: S2 File — (ZIP) [file pone.0201192.s003.zip › S2/HH04_06.png]

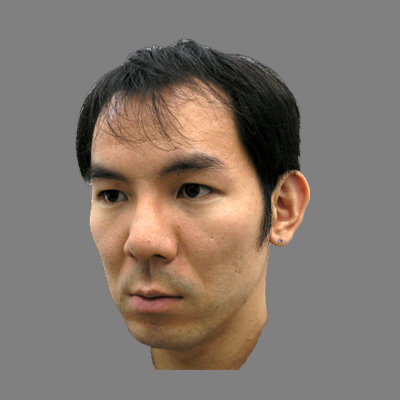

Supplement: S2 File — (ZIP) [file pone.0201192.s003.zip › S2/HH04_07.png]

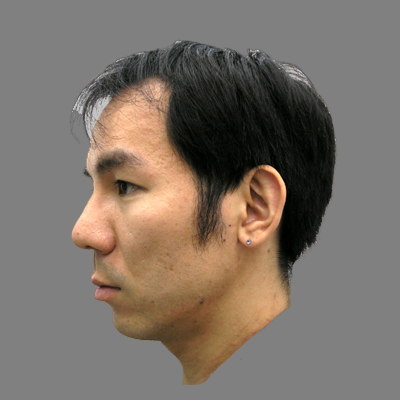

Supplement: S2 File — (ZIP) [file pone.0201192.s003.zip › S2/HH04_09.png]

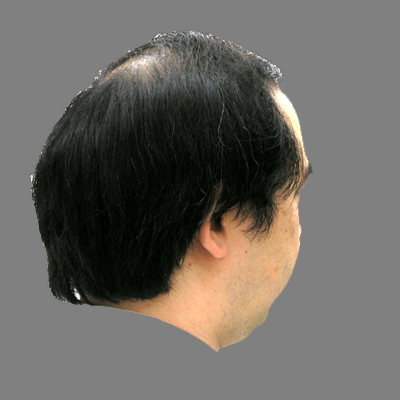

Supplement: S2 File — (ZIP) [file pone.0201192.s003.zip › S2/HH05_01.png]

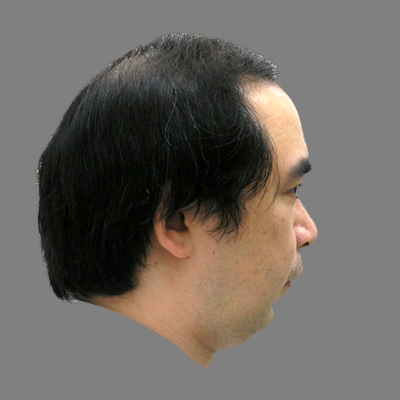

Supplement: S2 File — (ZIP) [file pone.0201192.s003.zip › S2/HH05_02.png]

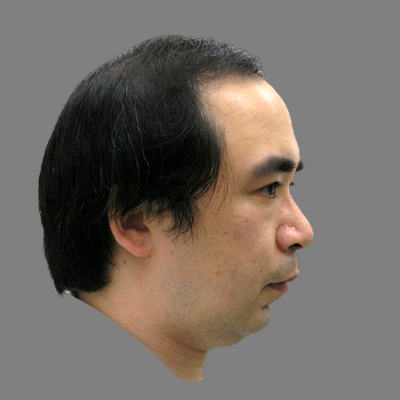

Supplement: S2 File — (ZIP) [file pone.0201192.s003.zip › S2/HH05_03.png]

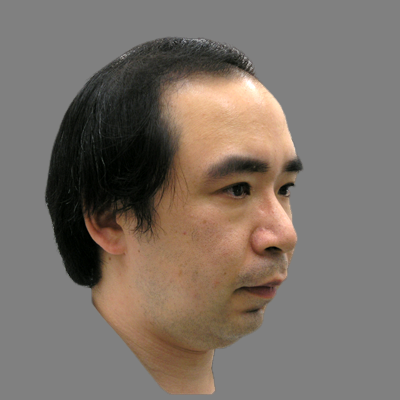

Supplement: S2 File — (ZIP) [file pone.0201192.s003.zip › S2/HH05_04.png]

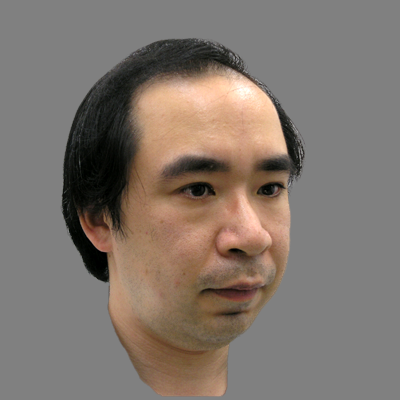

Supplement: S2 File — (ZIP) [file pone.0201192.s003.zip › S2/HH05_05.png]

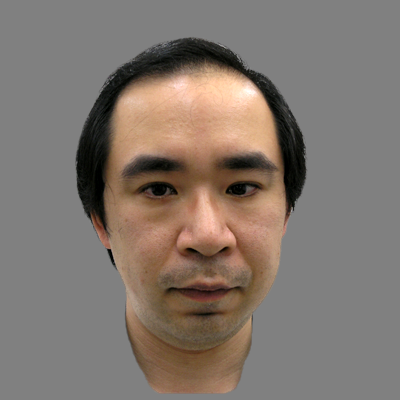

Supplement: S2 File — (ZIP) [file pone.0201192.s003.zip › S2/HH05_06.png]

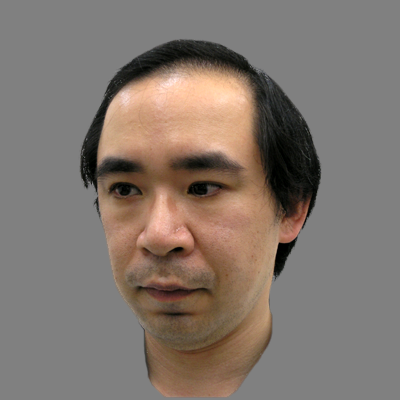

Supplement: S2 File — (ZIP) [file pone.0201192.s003.zip › S2/HH05_07.png]

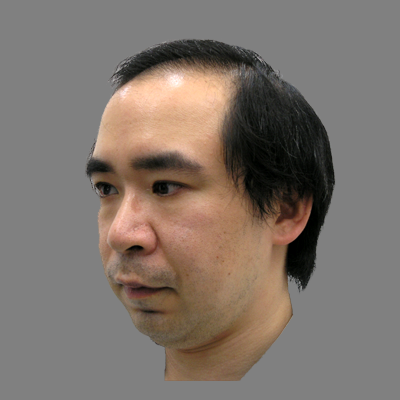

Supplement: S2 File — (ZIP) [file pone.0201192.s003.zip › S2/HH05_08.png]

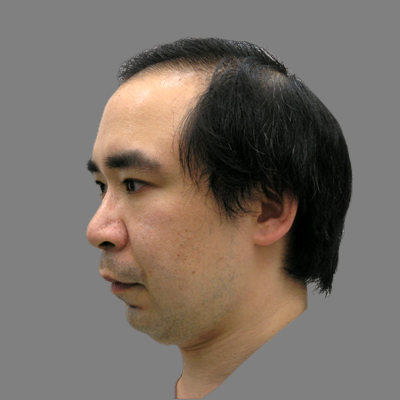

Supplement: S2 File — (ZIP) [file pone.0201192.s003.zip › S2/HH05_09.png]

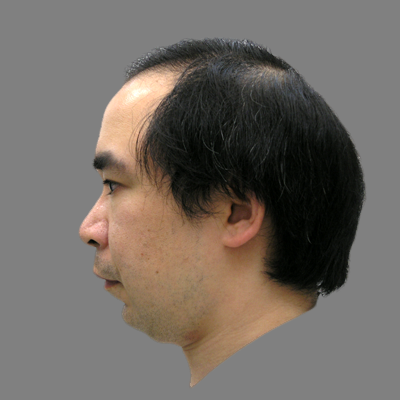

Supplement: S2 File — (ZIP) [file pone.0201192.s003.zip › S2/HH05_10.png]

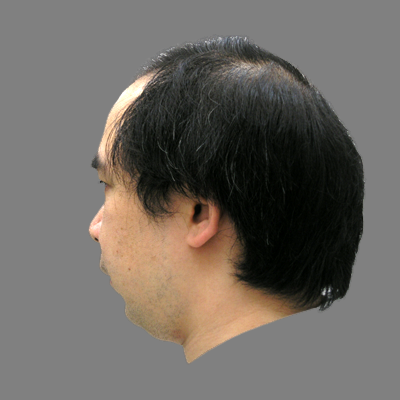

Supplement: S2 File — (ZIP) [file pone.0201192.s003.zip › S2/HH05_11.png]

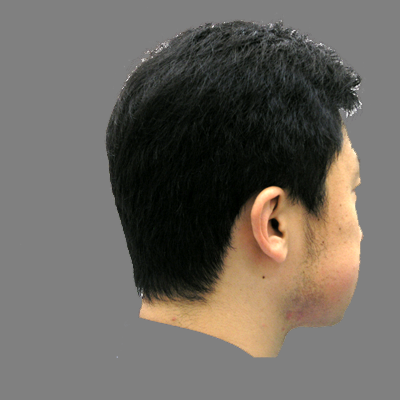

Supplement: S2 File — (ZIP) [file pone.0201192.s003.zip › S2/HH07_01.png]

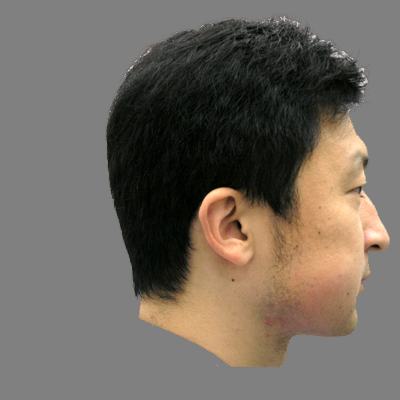

Supplement: S2 File — (ZIP) [file pone.0201192.s003.zip › S2/HH07_02.png]

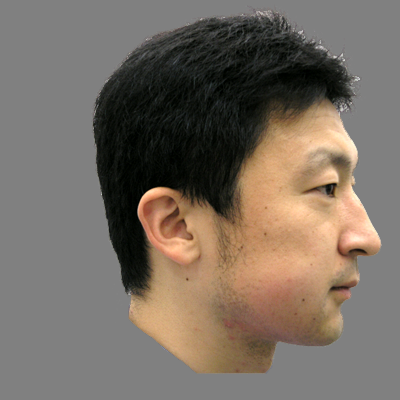

Supplement: S2 File — (ZIP) [file pone.0201192.s003.zip › S2/HH07_03.png]

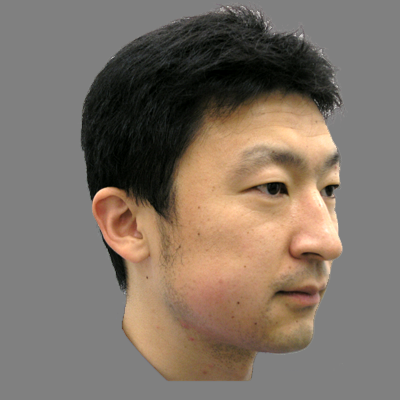

Supplement: S2 File — (ZIP) [file pone.0201192.s003.zip › S2/HH07_04.png]

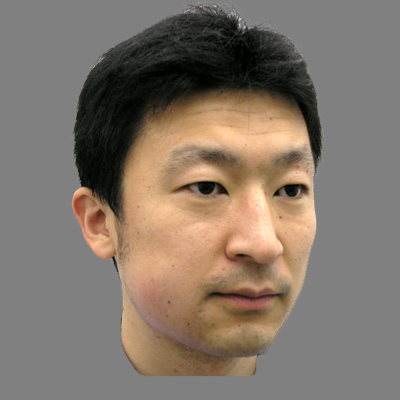

Supplement: S2 File — (ZIP) [file pone.0201192.s003.zip › S2/HH07_05.png]

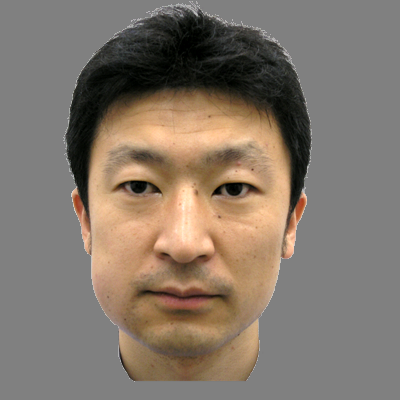

Supplement: S2 File — (ZIP) [file pone.0201192.s003.zip › S2/HH07_06.png]

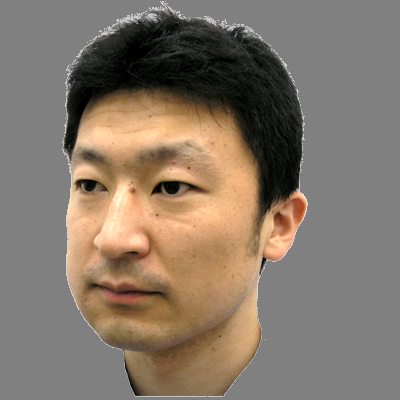

Supplement: S2 File — (ZIP) [file pone.0201192.s003.zip › S2/HH07_07.png]

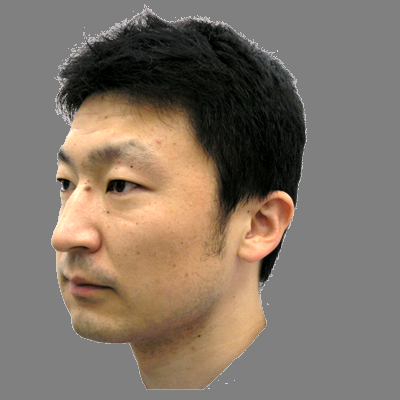

Supplement: S2 File — (ZIP) [file pone.0201192.s003.zip › S2/HH07_08.png]

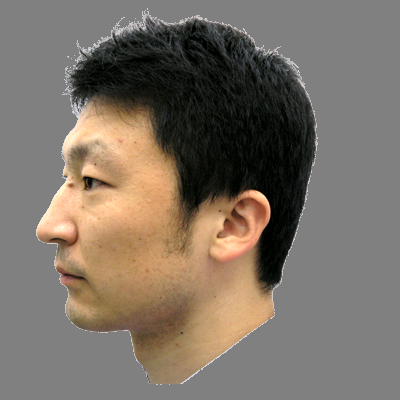

Supplement: S2 File — (ZIP) [file pone.0201192.s003.zip › S2/HH07_09.png]

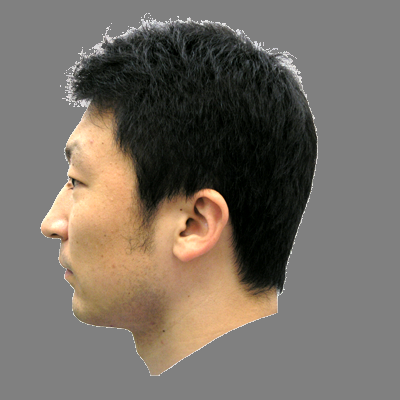

Supplement: S2 File — (ZIP) [file pone.0201192.s003.zip › S2/HH07_10.png]

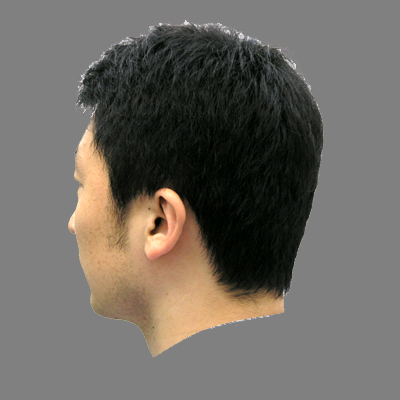

Supplement: S2 File — (ZIP) [file pone.0201192.s003.zip › S2/HH07_11.png]
